# The promise of deep urine proteomics for diagnosis of cancer, neurologic, and metabolic diseases

**DOI:** 10.1371/journal.pone.0354808

**Published:** 2026-07-30

**Authors:** Bogdan Budnik, Hossein Amirkhani, Klaus Weinberger, Saharnaz Nedjat, Mohammad H. Forouzanfar, Ashkan Afshin

**Affiliations:** Novelna Inc., Palo Alto, California, United States of America; Aarhus University, DENMARK

## Abstract

**Introduction:**

Urine offers a noninvasive and low-cost source of disease biomarkers, yet most proteomic studies have targeted single conditions. Using deep proteomic profiling and machine learning, we evaluated whether urinary protein signatures distinguish early-stage cancer, neurologic, and metabolic diseases from healthy controls.

**Methods:**

This case–control diagnostic accuracy study analyzed urine samples from the Ukraine Association of Biobank (UAB), collected during routine medical check-ups. The study included 22 patients each with kidney, bladder, melanoma, prostate, ovarian, endometrial, and cervical cancers; 22 with multiple sclerosis (MS); 22 with metabolic dysfunction–associated steatohepatitis (MASH); and 66 healthy controls, yielding 264 samples analyzed in September 2023. Proteomic assays were performed using the Olink Explore 3072 platform, with laboratory personnel blinded to disease status. Urine proteomes were profiled to identify disease-specific protein signatures. The primary outcome was diagnostic accuracy of multiprotein urine panels for each disease compared with healthy controls, expressed as the area under the receiver operating characteristic curve (AUC), along with sensitivity and specificity calculated at a prespecified threshold.

**Results:**

Expected sex‑specific differences (KLK3, MSMB higher in males; KLK8, KLK13 higher in females) supported assay validity. Three expression patterns were observed: (1) few strong, symmetric signals in melanoma and endometrial cancer; (2) asymmetry with many up‑regulated proteins in cervical, ovarian, and prostate cancers and in MS; and (3) broad up‑regulation in kidney and bladder cancers and in MASH. Multiprotein models outperformed single proteins, plateauing at five to seven. Maximum AUCs ranged from 0.88 (MS) to 0.98 0.97 (ovarian cancer), with AUCs ≥ 0.95 for seven of nine diseases. Some proteins (e.g., C9orf40, PPY) showed cross‑disease importance.

**Conclusions:**

Urine proteomics identified disease‑related signals across cancer and metabolic conditions and may enable accurate, noninvasive classification using multiprotein panels, but given the exploratory design and its limitations, the reported accuracies should be regarded as upper-bound estimates requiring prospective validation.

## 1. Introduction

Urine-based diagnostics are becoming increasingly popular as a non-invasive and cost-effective method of early detection across multiple organ systems [1–8]. In oncology, several urine-based assays have demonstrated clinical potential. For example, the genomic test UroSEEK detects mutations in 11 cancer-associated genes and has shown a sensitivity of 96% and specificity of 88% for early urothelial cancer [9]. Similarly, the EpiCheck assay targets 15 DNA methylation sites and achieves 68% sensitivity at 88% specificity [10]. While these nucleic acid–based tests exemplify important progress, they remain limited to specific genomic alterations within certain cancers. In contrast, urinary proteomics captures a much broader landscape of physiological and pathological processes, reflecting systemic molecular changes rather than isolated mutations. Recently, a 19-peptide urinary biomarker model demonstrated 87% sensitivity and 65% specificity, outperforming traditional markers and highlighting the promise of proteome-based diagnostics [11]. Together, these developments underscore the growing potential of urine as a versatile diagnostic medium and highlight the need for next-generation proteomic approaches to extend its clinical reach beyond organ-specific applications.

Although urine represents a highly informative and easily accessible biofluid, reflecting systemic metabolic and pathophysiologic states, its translation into routine diagnostic use remains limited by several challenges. While urine is often considered an extract and proxy of the serum, reliable quantification of proteins in urine for clinical testing is difficult [12]. The low abundance of many target biomarkers, the influence of renal physiology and urinary tract dynamics, and the high pre-analytical variability in sample collection, storage, and processing contribute to inconsistent results across studies and populations. Moreover, the identification of biomarkers that are both disease-specific and sufficiently sensitive for early detection has posed a persistent barrier to developing robust, widely deployable urine-based diagnostic tests [13].

Recent advancements in protein measurement technologies, particularly the Proximity Extension Assay (PEA), combined with powerful machine-learning algorithms, hold the transformative potential to reshape the landscape of urine-based diagnostics. These innovations enable the detection of low-abundance biomarkers in urine with unprecedented sensitivity and specificity, offering a more comprehensive and systems-level view of disease biology [14–20]. However, despite these technological advances, the application of deep urinary proteomics for multi-disease detection and cross-condition pattern recognition remains largely unexplored. Rather than focusing on single biomarkers, recent approaches allow the identification of multi-protein signatures that define disease-specific molecular patterns in urine. This shift toward multi-protein profiling offers the potential to develop broader and more reliable diagnostic panels for early disease detection. In this exploratory study, we address this gap by applying large-scale urinary proteomic profiling and machine-learning analysis to characterize disease-specific and cross-disease protein signatures across several early-stage conditions, establishing a framework for noninvasive, multi-disease diagnostics.

## 2. Methods

### 2.1. Study design

This case‑control diagnostic accuracy study used urine samples from the Ukraine Association of Biobank (UAB). Clinical diagnoses were established before sample banking, and proteomic analyses were performed in September 2023 on stored specimens, blinded to disease status. Established in 2017, the UAB upholds high ethical standards and predominantly represents the Ukrainian population [21].

This analysis included 264 urine samples from the UAB, comprising 198 patients with early-stage, treatment-naïve disease and 66 healthy controls recruited during routine medical check-ups. Disease groups included kidney, bladder, prostate, ovarian, cervical, endometrial, and melanoma cancers (n = 22 each), multiple sclerosis (MS; n = 22), and metabolic dysfunction–associated steatohepatitis (MASH; n = 22). Cancer and MASH were biopsy confirmed, and MS was confirmed by a neurologist’s evaluation. The sample size was designed for signal discovery and to optimize use of the proteomic assay panel.

### 2.2. Samples

Urine samples, diluted fourfold to reduce salt content, were analyzed on the Olink Explore 3072 platform, which uses antibody-based detection with next-generation sequencing (NGS). The assay, organized into eight 384-plex panels, targeted 2,850 proteins and incorporated internal and external controls for variability reduction and normalization. Each sample was processed in a single run: overnight antibody–protein incubation, probe hybridization with extension and pre-amplification, and subsequent DNA indexing for library preparation. Protein abundances were reported as Normalized Protein eXpression (NPX) values on a log2 scale, with higher NPX values reflecting greater protein concentrations. Urine samples were not subjected to creatinine normalization prior to Olink assay; the Olink platform incorporates internal extension and amplification controls to reduce intra- and inter-run technical variability, but variation due to urine concentration, hydration status, or urinary tract inflammation cannot be excluded. Renal function measures and creatinine levels were not present in the accessible study data and could therefore not be incorporated into the analysis. All biospecimens were collected, processed, and stored by the UAB following standardized procedures in accordance with ESBB, ISBER, and NCI biobanking guidelines, with urine samples aliquoted and stored at −80 °C and detailed metadata—including storage temperature and freeze–thaw history—systematically recorded [21].

### 2.3. Protein Measurements

The protein levels in urine were measured using Olink Explore 3072 technology. A comprehensive list of the measured proteins, including their characteristics, is available in Table A in S1 File. All protein measurements for the samples were conducted in a single run. In summary, this technology relies on antibody-based detection to quantify the levels of target proteins. Antibodies, each conjugated with two complementary probes, were organized into eight distinct 384-plex panels. Within each panel, three control assays (interleukin-6 (IL6), interleukin-8 (CXCL8), and tumor necrosis factor (TNF)) were included for quality control purposes.

The assay process commenced with an overnight incubation to facilitate the binding of conjugated antibodies to the target proteins in the samples. This was followed by an extension and pre-amplification step, enabling the hybridization and extension of complementary probes. The extended DNA was subsequently amplified via PCR and indexed to prepare libraries, which were then subjected to sequencing on the Illumina NovaSeq platform. The resulting sequencing counts underwent a rigorous quality control and normalization procedure, incorporating internal controls to minimize intra-assay variability. These internal controls encompassed an incubation control with a non-human antigen, an extension control with a unique probe pair, and an amplification control with a double-stranded DNA sequence. Additionally, external controls, including a negative control (buffer sample) and plate controls (a plasma pool), were employed to establish the limit of detection and standardize measurements between plates. Finally, two known samples were used as controls to assess the precision of the measurements. Following quality control and normalization, the data was presented in NPX units, logarithmically transformed (log2 scale), with higher NPX values indicating higher protein levels. For quality purposes, proteins with more than 30% QC warnings, more than 30% assay warnings, or more than 50% missing frequency were excluded. Olink’s panel performance has been extensively validated for various analytical parameters, including sensitivity, dynamic range, specificity, precision, and scalability. The analytical measuring range was determined by establishing the lower limit of quantification (LLOQ) and upper limit of quantification (ULOQ) for each analyte, reported in pg/mL. Additionally, the potential occurrence of a high-dose hook effect, characterized by antigen excess in comparison to the reagent antibodies, leading to erroneously lower values, has been identified for all analytes (Table A in S1 File). All assays have undergone rigorous validation for precision (encompassing repeatability and reproducibility). Intra-assay variation (within a single run) was calculated by determining the mean coefficient of variation (CV) across 6 individual samples in each of 7 separate runs conducted during the validation studies. Likewise, inter-assay variation (between different runs) was computed as the mean CV across the same 6 individual samples, measured across 7 separate runs during the validation studies.

### 2.4. Statistical analysis

The analysis had two primary objectives: (1) to assess the bivariate relationship between disease and healthy samples using volcano plots of NPX averages and p-values, and (2) to identify a minimal protein set for disease classification. Given the limited sample size, a two -step approach was adopted to identify a minimal set of proteins capable of classifying samples within a stable model, ensuring general applicability.

In the first phase, random forest analysis was used for initial feature screening; in the second phase, LightGBM classifiers were trained on 100 bootstrap resamples to derive a stable minimal feature set, followed by additional LightGBM training to assess exploratory model stability using the selected biomarkers. The predicted probability for each sample was compared with a threshold to determine whether it was classified as healthy or diseased, with the threshold adjusted to achieve the desired level of specificity. Model performance was evaluated using the area under the receiver operating characteristic (ROC) curve (AUC) within an iterative leave-one-out validation process.

Feature importance scores derived from random forest analysis quantified each protein’s contribution to reducing classification impurity across disease–healthy comparisons. To evaluate cross-disease relevance, concatenated panels were reanalyzed across all disease groups, with normalized Gini-based importance scores reflecting each protein’s relative contribution to classification accuracy and the reduction of sample heterogeneity.

All preprocessing and modeling procedures were conducted in Python (version 3.9.13) using Scikit-learn (version 1.1.2), and supporting libraries including pandas (v1.4.4), numpy (v1.23.3), and statsmodels (v0.13.2) [22–25]. For data visualization, we used matplotlib (version 3.6.0), seaborn (version 0.12.0), and plotly (version 5.10.0) [26–28]. All protein-wise comparisons were two-sided. No correction for multiple comparisons was applied to the 2,850 protein-wise comparisons. The resulting p-values are used solely to rank and visualize proteins for downstream feature selection; they do not support inference regarding differential expression of any individual protein.

### 2.5. Pathway and functional enrichment analysis

Pathway and functional enrichment analyses were performed using the Reactome database (version 96) via over-representation analysis. For each disease-specific urinary protein panel, enrichment of Reactome pathways was assessed using a hypergeometric test comparing the observed number of proteins in each pathway to that expected by chance based on the human reference proteome. Resulting p-values were adjusted for multiple testing using the Benjamini–Hochberg false discovery rate (FDR) method, and pathways with FDR-adjusted p-values <0.05 were considered statistically significant. Analyses were restricted to Homo sapiens, and non-human identifiers were mapped to their human orthologs where applicable.

### 2.6. Patient and public involvement

Considering that this was an initial proof-of-concept study, patients and the public were not directly involved in its design. However, they will play a critical role in the dissemination of these preliminary results and in the subsequent validation phase of the research.

### 2.7. Ethics statement

This study analyzed archived, de-identified urine specimens obtained from the UAB. The UAB Institutional Review Board reviewed the study and determined that it was exempt from formal human-subjects review, as all biospecimens and associated data were fully anonymized prior to analysis.

The urine samples were originally collected by the UAB during routine medical check-ups, under their established ethical protocol that includes broad informed consent for future research use of de-identified materials. For this specific project, the requirement for additional individual informed consent was formally waived by the UAB IRB in accordance with institutional and national regulations.

No new participants were prospectively recruited for this research, and investigators had no access to personally identifiable information at any stage. The study adhered to the principles of the Declaration of Helsinki and all relevant ethical regulations for research involving human participants.

## 3. Results

Overall, 2,850 proteins were measured in 264 samples, including early‑stage kidney (11 male, 11 female), bladder (14 male, 8 female), melanoma (16 male, 6 female), prostate, ovarian, endometrial, and cervical cancers, MS (16 male, 6 female), and MASH (9 male, 13 female), plus 66 healthy individuals (33 male, 33 female).

### 3.1. Urine proteome landscape in healthy individuals

We utilized the PEA technology to detect a total of 3,072 proteins in urine samples and successfully identified 2,850 of these proteins. This subset of 2,850 proteins formed the basis for the exploration of novel biomarkers in urine. Notably, all proteins were directly assessed from urine samples with a fourfold dilution, primarily aimed at minimizing the presence of salt in the samples. No further dilution was necessary for the PEA technology analysis, suggesting that the concentrations of the detected proteins generally remained at lower levels across the entire spectrum of proteins examined.

**Fig 1:**
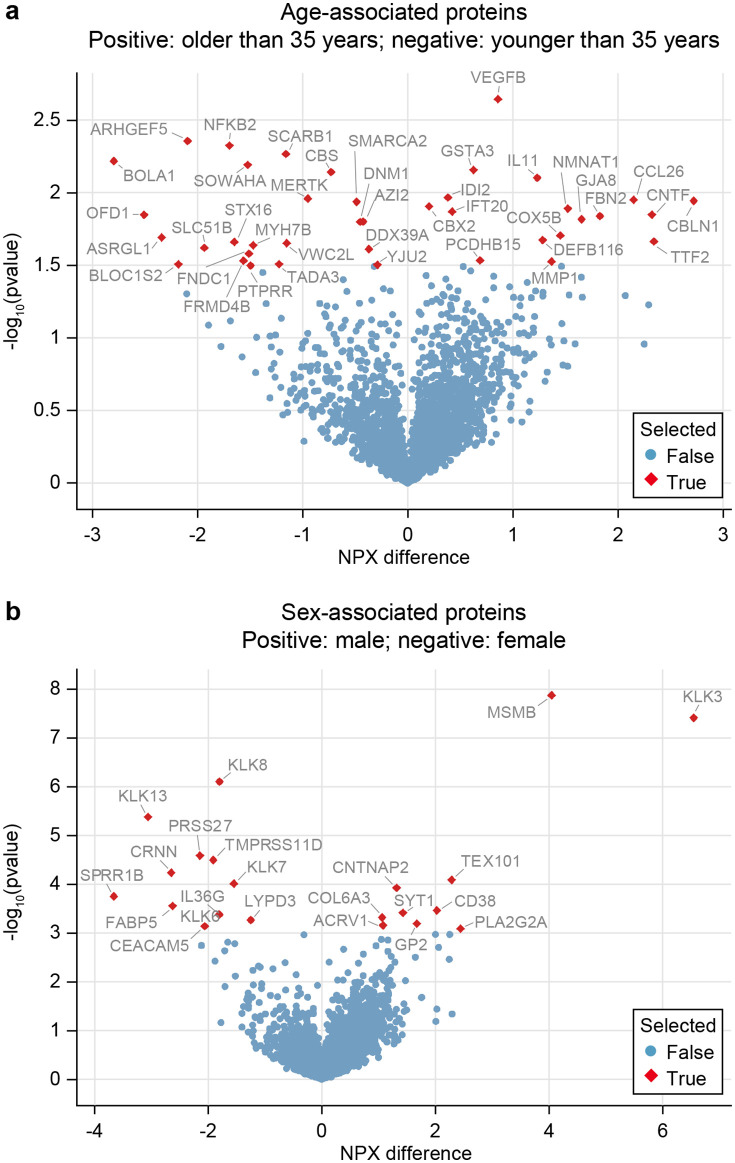
Variations in the urine proteome by age and sex. Volcano plots depicting differential protein abundances in healthy adults aged above and below 35 years (a), and between adult males and females (b). Red points indicate proteins with the lowest p-values; p-values are not corrected for multiple testing and are presented for visualization rather than formal inference.

Our analysis indicated that the concentrations of certain proteins did not display significant differences in relation to the age of the individuals. As illustrated in Fig 1A, specific proteins such as VEGFB and IL11 exhibited relatively higher urinary concentrations in older participants, whereas proteins including BOLA1, ARHGEF5, and NFKB2 showed lower concentrations. Only a limited number of proteins demonstrated p < 0.01, and the lowest observed p-value was 0.002, indicating overall subtle age-related variation in urinary protein concentrations.

Furthermore, urinary protein concentrations varied according to biological sex. Fig 1B depicts these differences in protein abundances between healthy males and females. Notably, Kallikreins were highly sensitive to sex differences: KLK3 and MSMB showed higher concentrations in male urine samples (both p < 1 × 10 ^−^ ⁷), while KLK8 and KLK13 were higher in female samples (both p < 1 × 10 ^−5^ ). In total, more than 20 proteins exhibited p < 0.001, suggesting a more pronounced proteomic contrast by sex than by age.

### 3.2. Urine proteome landscape across diseases

**Fig 2:**
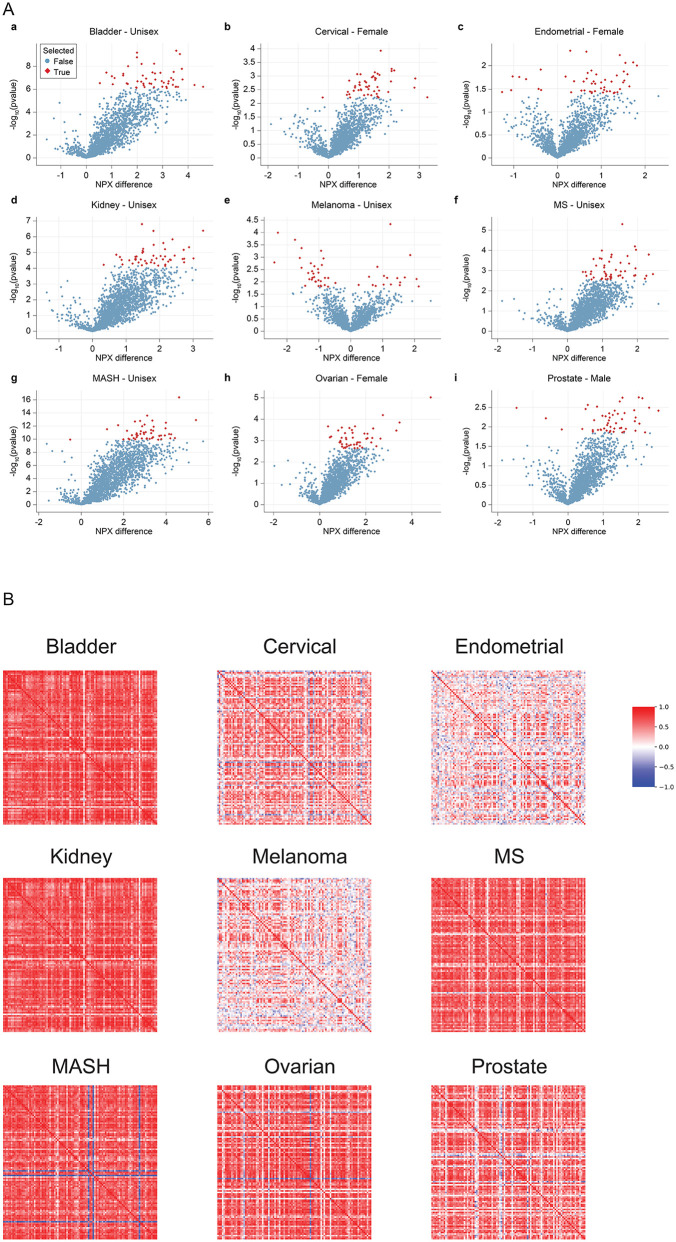
Disease-associated urinary protein signatures: volcano plots and correlation structure. 2A. Volcano plots display differential protein abundances for 2,850 proteins per disease (x-axis, NPX difference between urine samples from patients and healthy controls, where NPX is a log_2_-scaled relative abundance unit; y-axis, − log₁₀(p) of the difference between groups). They are exploratory, hypothesis-generating, and non-inferential. P values are unadjusted for multiple testing, and the plots are intended only for visualization and feature-selection purposes; they should not be interpreted as statistically confirmed evidence of differential protein expression. Red points represent the proteins with the lowest p-values. Conditions include bladder cancer (a), cervical cancer (b), endometrial cancer (c), kidney cancer (d), melanoma (e), multiple sclerosis (MS) (f), metabolic dysfunction–associated steatohepatitis (MASH) (g), ovarian cancer (h), and prostate cancer (i). 2B. Pairwise correlation heatmaps of protein abundances within each disease cohort, for the same conditions (a–i) as 2A.

The volcano plots illustrated in Fig 2A provide an overview of differential protein expression across the diseases examined. Because these volcano plots are based on unadjusted p-values, they are intended solely as exploratory, descriptive, and non-inferential visualizations for hypothesis generation. Most conditions exhibited an asymmetric distribution, with an overrepresentation of proteins elevated in patients compared with healthy controls, except for melanoma. Three characteristic patterns emerged. First, in melanoma and, to a lesser extent, endometrial cancer, the volcano plots were largely symmetrical, showing few large-magnitude signals. Second, in cervical, ovarian, and prostate cancers as well as in MS, an asymmetric pattern was observed, although most protein changes were of small magnitude. Third, kidney, bladder, and MASH displayed pronounced asymmetry, with increases in most proteins. These findings underscore the influence of renal excretion on urinary proteomic profiles, which has important implications for disease biomarker discovery. The correlation heatmaps in Fig 2B further illustrate consistent clustering patterns within each disease group, supporting the observed disease-associated urinary proteomic patterns.

Pairwise correlation matrices among the top 100 proteins with the lowest p-values revealed distinct disease-specific correlation structures in both patient and healthy control groups (Fig A in S1 File). Venn diagrams of over- and under-represented proteins across conditions (Fig B in S1 File) further illustrated the overlap and specificity of urinary proteomic signatures across disease groups.

### 3.3. Optimal disease-specific protein signature

**Fig 3:**
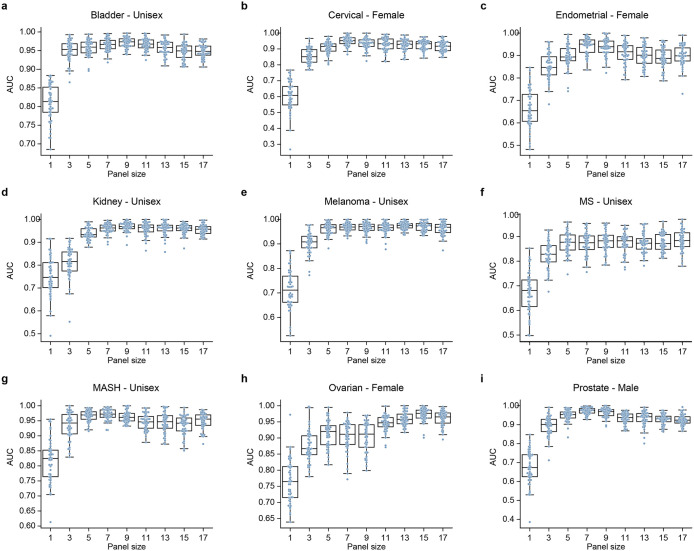
Relationship between the size of the protein panel and its diagnostic performance. Boxplots display the area under the receiver operating characteristic curve (AUC) across 100 bootstrap iterations for varying panel sizes. The x-axis represents the number of proteins in the panel, and the y-axis shows model performance (AUC). Conditions include bladder (a), cervical (b), endometrial (c), kidney (d), melanoma (e), multiple sclerosis (f), metabolic dysfunction–associated steatohepatitis (MASH) (g), ovarian (h), and prostate (i). Y-axis scales are disease-specific and optimized to show the plateau behavior of each panel; they are not intended for direct cross-disease AUC comparison. For standardized AUC values and 95% confidence intervals across all disease groups, see Table B in S1 File.

Across all disease groups, the classifier’s out-of-sample performance improved with the inclusion of additional proteins, reaching a plateau around five to seven proteins, suggesting a feasible basis for multiprotein urine tests. While this trend was generally consistent, performance saturation occurred more rapidly in certain conditions, potentially reflecting non-differential protein excretion patterns (Fig 3).

Optimal disease-specific performance, defined by AUC values exceeding 0.90, was typically achieved with panels containing at least seven proteins. Ovarian cancer represented an exception, requiring a 15-protein panel to reach maximal accuracy, whereas multiple sclerosis (MS) achieved a lower peak AUC of 0.88 with a five-protein panel, remaining stable up to 17 proteins.

**Fig 4:**
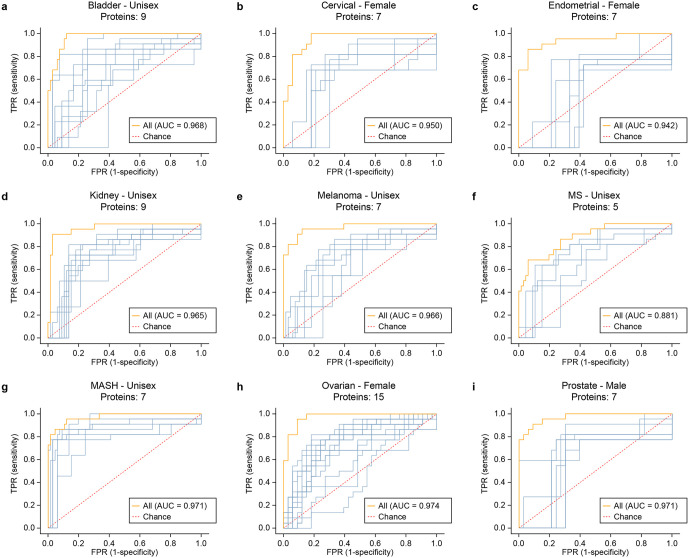
ROC curve performance of the selected multiprotein panels across disease groups. Receiver operating characteristic (ROC) curves depict classification performance for each disease model: bladder (a), cervical (b), endometrial (c), kidney (d), melanoma (e), multiple sclerosis (f), metabolic dysfunction–associated steatohepatitis (MASH) (g), ovarian (h), and prostate (i). Blue lines represent individual protein classifiers, and orange lines represent the combined multiprotein panel. The dashed red line indicates random classification.

The discriminative performance of each disease-specific multiprotein panel is illustrated in Fig 4, with ROC curves demonstrating strong classification accuracy across all nine disease groups. Across most diseases, the performance of the multiprotein panel exceeded that of any single protein, underscoring the additive contribution of each marker to overall model accuracy. The urinary panels achieved strong discrimination (AUC ≥ 0.95) for seven of nine diseases, with a measurable trade-off between sensitivity and specificity across conditions. The optimal panel sizes and corresponding AUC values for all disease groups are summarized in Table B in S1 File. For prostate cancer, MASH, and melanoma, relatively high sensitivity could be obtained at 99% specificity, whereas for cervical and endometrial cancers, high sensitivity required a substantial reduction in specificity. These findings confirm that disease-specific combinations of proteins provide robust yet variable discriminative performance across biological contexts.

**Fig 5:**
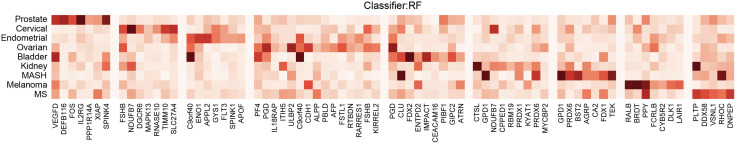
Distinctive significance of protein sets for disease detection. The heatmap displays normalized Gini impurity–derived feature importance values for each protein across disease-specific random forest classifiers. Each cell represents the relative contribution of a protein to model accuracy, with color intensity reflecting normalized Gini importance scores on a relative scale from 0 [white, no contribution] to the maximum observed value [dark red]. The figure highlights both disease-specific and cross-disease protein relevance among the optimized classification panels.

Fig 5 presents a heatmap visualization of the normalized Gini impurity–derived feature importance values across different disease models. Each cell represents the relative contribution of an individual protein to disease classification accuracy, with color intensity reflecting normalized Gini importance scores on a relative scale from 0 (white) to the maximum observed value (dark red). This visualization highlights the unique significance of protein sets in distinguishing specific diseases.

Most disease detection sets typically comprised seven proteins, except for the ovarian cancer panel, which required fifteen proteins to achieve an acceptable level of specificity. Proteins exhibiting the highest predictive specificity for their respective diseases were the most dominant contributors. Notably, several proteins demonstrated cross-disease relevance. For example, VEGFD, included in the prostate cancer panel, also showed high importance in bladder cancer. Similarly, C9orf40 displayed substantial importance for bladder cancer, even though it was not retained in the final classifier, and PPY contributed equivalently to the detection of both melanoma and MS. Collectively, these findings underscore that the diagnostic performance of each panel arises not from single dominant proteins but from a coordinated combination of disease-specific and cross-disease informative markers.

### 3.4. Pathway and functional enrichment analysis

Pathway and functional enrichment analyses demonstrated significant enrichment of biologically coherent pathways for eight of the nine disease panels, with top-ranked pathways achieving FDR-adjusted p-values ranging from ~10 ^−^ ⁴ to <0.05. For the melanoma panel, no Reactome pathway reached the conventional FDR < 0.05 threshold; the top-ranked pathways (O_2_/CO_2_ exchange in erythrocytes, p38MAPK, RAS and NOTCH1 signaling, FDR ≈ 0.06–0.07) are reported as suggestive rather than confirmatory.

Cancer-associated panels were enriched for signal transduction and receptor tyrosine kinase pathways, including STAT3 nuclear events downstream of ALK signaling and broader receptor tyrosine kinase signaling, as well as hormone-related pathways such as estrogen-dependent signaling, glycoprotein hormone pathways, and peptide hormone biosynthesis in hormonally driven cancers. Additional significantly enriched pathways included cell adhesion and epithelial integrity processes (e.g., CDH1 regulation, Type I classical cadherin regulation, and cell–cell communication), transcriptional regulation programs (including NPAS4- and MITF-related pathways), apoptosis pathways (e.g., SMAC/XIAP-mediated caspase activation and apoptosome-related processes), and oxidative stress and detoxification pathways mediated by NFE2L2. Immune and cytokine signaling pathways (including interleukin-4 and interleukin-13 signaling) and cellular response-to-stimuli processes were also represented. In the endometrial panel, the majority of FDR < 0.05 hits derived from a single FLT3 identifier producing multiple near-identical TKI-resistance pathways, and this cluster should therefore be interpreted as a single mechanistic axis rather than independent enrichments.

In multiple sclerosis, the strongest enrichment was observed in NR1H2/NR1H3 (LXR)-mediated signaling and HDL/plasma lipoprotein remodeling (top-pathway FDR ≈ 10 ^−^ ⁴), consistent with the established role of nuclear-receptor–regulated cholesterol homeostasis in myelin biology; immune and antiviral pathways including NF-κB and interferon response signaling were also represented at lower ranks (FDR ≈ 0.035–0.04).

In metabolic dysfunction–associated steatohepatitis, the top enrichments included reversible carbon dioxide hydration (FDR ≈ 0.003) and FOXO-mediated transcription of oxidative-stress, metabolic, and neuronal genes (FDR ≈ 0.014), alongside interferon-α/β signaling (FDR ≈ 0.037).

Additional enriched pathways represented across panels included transport and small-molecule metabolism processes (e.g., fatty acid transport in cervical cancer), reflecting systemic physiological contributions. O_2_/CO_2_ exchange in erythrocytes was also represented in MASH and melanoma, although these pathways did not reach the conventional FDR < 0.05 threshold in either panel (FDR ≈ 0.05–0.07) and are therefore noted as trending rather than significantly enriched. While broader categories such as signal transduction were observed across panels, pathway-level enrichment patterns revealed distinct mechanistic profiles. Collectively, these findings support the biological relevance of the identified urinary protein panels and their alignment with established molecular pathways.

## 4. Discussion

This study provides a comprehensive characterization of the urinary proteome across nine early-stage diseases. It demonstrates that diagnostically informative protein signatures can be identified through deep urinary proteome profiling combined with machine-learning analysis. By quantifying 2,850 urinary proteins from 264 participants, we delineated disease-specific proteomic patterns that distinguished disease cases from healthy controls in this exploratory cohort. Optimal model performance was achieved with compact multi-protein panels, typically comprising seven proteins, yielding AUC values above 0.90 for most conditions, with slightly lower performance for MS (AUC = 0.88, with a 5-protein panel, with performance remaining stable even when expanding to 17 proteins) and a 15-protein panel necessary for ovarian cancer. Urinary proteomics captures both systemic biochemical changes and renal influences, offering insight into disease biology but also introducing interpretive complexity. Variations in protein abundance may arise from kidney or urinary tract alterations that compromise the blood–urine barrier, allowing larger proteins (> 45 kDa) to pass [29], or from systemic processes such as inflammation and metabolic stress. Distinguishing disease-specific signatures from renal background signals therefore remains essential for accurate interpretation, a principle exemplified by the CKD273 classifier for chronic kidney disease [30]. Integrating this contextual understanding into analytic models enhances the interpretability of urinary proteomic data and strengthens its translational relevance for systemic disease detection.

Previous urinary proteomics studies have primarily examined single diseases such as coronary artery disease, chronic kidney disease, or urologic malignancies [4,5,30–32], demonstrating the feasibility of identifying disease-associated urinary proteins but offering limited comparability across conditions. In contrast, our study applies a unified analytical framework across multiple disease classes, showing that multiplexed proteomic strategies can capture both shared biological mechanisms, such as inflammation, endothelial activation, and metabolic dysregulation, and distinct disease-specific signatures within a single urine proteome platform. These findings are further supported by pathway enrichment analyses, demonstrating that the identified protein panels map to biologically coherent pathways consistent with known disease mechanisms.

An important consideration when interpreting these findings is that urinary protein abundance is influenced not only by disease-associated biology but also by physiological variation in urine concentration and renal handling of proteins. Because urinary creatinine, osmolality, specific gravity, and renal function measurements were not available in the present study, the observed differences in protein abundance cannot be fully disentangled from variability related to urine concentration, glomerular filtration, protein excretion, or other renal physiological factors. Consequently, although the identified protein panels demonstrated promising discriminatory performance, the disease specificity of individual urinary protein changes should be interpreted cautiously.

Applying machine learning to multiplexed proteomic data enabled consistent disease classification across diverse conditions. Bladder cancer reached a mean AUC of 0.97 with a nine-protein panel, representing promising performance that warrants prospective validation before frontline use. Prostate cancer and MASH models showed similar discrimination, with high specificity, though confirmatory diagnostic utility requires prospective validation. Other cancers, including cervical, endometrial, kidney, melanoma, and ovarian, maintained strong performance across moderate panel sizes with early convergence. These results align with previous proteomic studies [11,33]. Overall, the cross-disease analysis demonstrated strong accuracy for oncologic and metabolic diseases but revealed persistent limitations in MS, where urinary proteomic signals were weaker, likely reflecting restricted passage of brain-derived proteins across the blood–brain barrier (BBB). This observation is consistent with prior evidence that urine, being anatomically distant from the central nervous system, contains only trace amounts of brain-derived biomolecules due to the impermeability of the BBB [34].

However, the findings of this study extend prior evidence supporting the feasibility of urinary biomarkers for noninvasive disease detection. Earlier genomic and methylation-based assays, such as UroSEEK and EpiCheck, have demonstrated the diagnostic potential of urine-derived nucleic acids for specific cancers. Yet these approaches remain limited to static genetic alterations and often fail to capture the functional and dynamic aspects of disease biology [10,35]. In contrast, urinary proteomics provides a more comprehensive snapshot of the physiological state, integrating signals from multiple organs and reflecting ongoing cellular activity, inflammation, and metabolic stress. Proteins and peptides serve as proximal effectors of pathophysiologic processes, and their abundance patterns can therefore reveal both disease onset and progression. For instance, multi-protein panels developed for prostate and bladder cancers have achieved strong discrimination, with the former reaching sensitivities above 85% and the latter attaining an AUC of 0.82 in independent validation [11,33]. Similarly, urinary proteomic classifiers have achieved high accuracy in cardiometabolic diseases, with peptide-based panels for coronary artery disease reporting AUCs above 0.90 and for diabetic kidney disease AUCs of 0.93 in validation cohorts [36,37]. Our findings are consistent with previous reports demonstrating that compact, multiplexed urinary protein panels can achieve high diagnostic accuracy across diverse diseases within a single assay. Moreover, proteomic assays based on antibody- or aptamer-based multiplexing are generally more cost-efficient and scalable than sequencing or methylation assays, supporting their suitability for broader clinical implementation. Our findings indicate that several proteins, including VEGFD, C9orf40, and PPY, contributed across disease models, reflecting both distinct disease-specific patterns and shared systemic mechanisms. These results demonstrate that multiplexed urinary proteomic strategies can capture convergent biological pathways, such as inflammation, endothelial activation, and metabolic dysregulation, while preserving the resolution needed to distinguish individual disease signatures within a unified analytical framework.

The observed sex-related differences, particularly within the kallikrein family (KLK3, MSMB, KLK8, KLK13), parallel previous findings of sex-dependent urinary protein expression [38,39]. In contrast, the minimal influence of age observed here is consistent with overall stability of urinary protein concentrations across adulthood. These findings reinforce the biological plausibility, analytical robustness, and translational promise of deep urinary proteomics as a foundation for future multi-disease diagnostic development.

In this discovery cohort, multi-protein panels demonstrated promising sensitivity in identifying early-stage tumors in asymptomatic individuals, underscoring the exploratory potential of urinary proteomics for population-level screening pending prospective validation. The simplicity, safety, and cost-effectiveness of urine sampling further enhance its suitability for large-scale implementation, particularly among individuals at elevated risk due to family history or lifestyle factors. By enabling earlier detection and targeted follow-up, such approaches may have the potential to improve clinical outcomes and reduce healthcare costs, pending prospective validation.

Urine proteomics also complements existing diagnostic paradigms by capturing systemic biochemical changes. Because alterations in the urinary proteome can reflect renal or urinary tract physiology, distinguishing disease-specific signals from kidney-derived changes remains essential. Beyond analytical depth, a key strength of this study lies in the machine learning framework developed to extract discriminative features from high-dimensional proteomic data. This approach enabled the derivation of minimal yet accurate protein signatures across multiple diseases, and identifying candidate signatures for evaluation in future prospective studies. These findings highlight the exploratory translational potential of urinary proteomics, which may contribute to early disease detection if validated in future prospective studies.

### Limitations

Several limitations of this study should be considered when interpreting the findings. First, the case–control design using archived biobank specimens, while appropriate for discovery-phase diagnostic research, may overestimate diagnostic performance compared with prospective clinical settings. Case and control samples were drawn from distinct cohorts without matching or statistical adjustment for age and sex. Because cases and controls were recruited separately, cohort membership almost entirely coincides with disease status. The observed proteomic differences may therefore reflect cohort-related bias in pre-analytical handling, physiology, or demographic composition rather than disease biology. Age-related variation in urinary protein levels was minimal in this study, which limits age specifically as a source of such bias but does not address the other pathways through which cohort bias may act. Second, the sample size for each disease group was modest (n = 22 per group), which, although adequate for signal discovery and optimized for the proteomic assay panel, reduces statistical power and increases the risk of overfitting despite the leave-one-out validation strategy employed. Replication in larger, independently collected cohorts is necessary to confirm the robustness of the identified panels. The 100-iteration bootstrap resampling procedure was used to assess classifier stability, providing approximate rather than formal inferential confidence intervals. Mean AUC estimates were derived from leave-one-out cross-validation and are independent of the number of bootstrap iterations, although confidence interval precision may be limited by the relatively small number of resamples.

Third, urinary protein concentrations were not adjusted for hydration status, creatinine levels, osmolality, or specific gravity measurements — physiological variables known to influence protein excretion rates. This may introduce additional variability and potentially confound disease-specific signals, particularly for conditions not directly involving the kidney or urinary tract. Systematic measurement and correction of these variables — through creatinine normalization or osmolality adjustment — are identified as priorities for the planned prospective validation studies.

Fourth, all samples were derived from a single biobank representing predominantly the Ukrainian population, limiting ethnic and geographic diversity. The identified biomarker panels may not perform equivalently across populations with different genetic backgrounds, comorbidity profiles, or environmental exposures.

Finally, this study lacks external or prospective validation; all performance estimates are derived from the same biobank dataset, and independent validation in prospectively collected cohorts is required before any clinical utility can be established.

## 5. Conclusions

This exploratory, hypothesis-generating study identifies candidate urinary protein signatures that distinguish multiple early-stage conditions from healthy controls using compact, disease-specific protein panels. These findings support the feasibility of urine as a noninvasive matrix for disease biomarker discovery. However, because the observed proteomic differences cannot be fully disentangled from urine concentration-related variability, the identified signatures should be regarded as preliminary and require confirmation in prospectively standardized cohorts. Standardization of urine collection and handling procedures, together with adjustment for renal and urinary tract factors, will be essential to improve reproducibility. Future work should include external and longitudinal validation to confirm these findings and support translation toward clinical application.

## Supporting information

S1 File Supporting figures and tables. Fig A. Pairwise correlation matrices of the top 100 differentially represented proteins between patients and healthy controls. Matrices are displayed separately for healthy controls (A) and patients (B) across nine disease groups: bladder cancer, cervical cancer, endometrial cancer, kidney cancer, melanoma, multiple sclerosis, metabolic dysfunction–associated steatohepatitis (MASH), ovarian cancer, and prostate cancer. Color scale represents Pearson correlation coefficients ranging from −1 (blue) to +1 (red). Fig B. Venn diagrams illustrating the overlap of differentially abundant urinary proteins across disease groups. Shared and unique proteins are presented separately for overrepresented proteins in female-predominant cancers (A), overrepresented proteins in mixed-sex and other disease groups (B), and underrepresented proteins (C). Proteins were selected for display using a p < 0.05 threshold relative to healthy controls. This threshold is descriptive only: no correction for multiple testing was applied. Table A. Proteins included in the Olink Explore 3072 analysis and their assay characteristics. UniProt identifier, panel assignment, LOD, LLOQ, ULOQ, hook concentration, dynamic range (log10), and intra- and inter-assay CVs for each assay. Table B. Diagnostic performance of multiprotein urine panels across disease groups. Optimal panel size, mean AUC, 95% confidence interval, and performance trend for each of the nine disease groups.
(PDF)
