# Supplementary material for: The promise of deep urine proteomics for diagnosis of cancer, neurologic, and metabolic diseases
Source: PLoS One. 2026 Jul 30;21(7):e0354808. doi: 10.1371/journal.pone.0354808 (PMC13422848; doi:10.1371/journal.pone.0354808)
Supplement: S1 File — Fig A. Pairwise correlation matrices of the top 100 differentially represented proteins between patients and healthy controls. Matrices are displayed separately for healthy controls (A) and patients (B) across nine disease groups: bladder cancer, cervical cancer, endometrial cancer, kidney cancer, melanoma, multiple sclerosis, metabolic dysfunction–associated steatohepatitis (MASH), ovarian cancer, and prostate cancer. Color scale represents Pearson correlation coefficients ranging from −1 (blue) to +1 (red). Fig B. Venn diagrams illustrating the overlap of differentially abundant urinary proteins across disease groups. Shared and unique proteins are presented separately for overrepresented proteins in female-predominant cancers (A), overrepresented proteins in mixed-sex and other disease groups (B), and underrepresented proteins (C). Proteins were selected for display using a p < 0.05 threshold relative to healthy controls. This threshold is descriptive only: no correction for multiple testing was applied. Table A. Proteins included in the Olink Explore 3072 analysis and their assay characteristics. UniProt identifier, panel assignment, LOD, LLOQ, ULOQ, hook concentration, dynamic range (log10), and intra- and inter-assay CVs for each assay. Table B. Diagnostic performance of multiprotein urine panels across disease groups. Optimal panel size, mean AUC, 95% confidence interval, and performance trend for each of the nine disease groups. (PDF) [file pone.0354808.s001.pdf]

Figure A. Correlation Matrices of the Top 100 Differentially Represented Proteins between Patients and Healthy Controls.
Matrices are displayed separately for healthy controls (A) and patients (B).

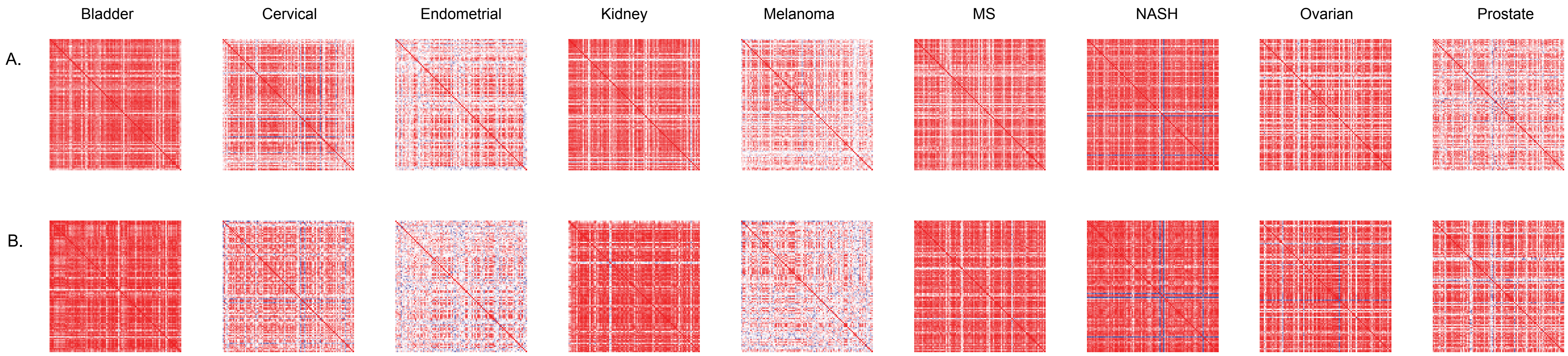

Figure B. Venn Diagrams Illustrating Significantly Altered Proteins Across Various Diseases.
The shared proteins are presented separately for overrepresented proteins (A, B) and underrepresented proteins in urine (C).

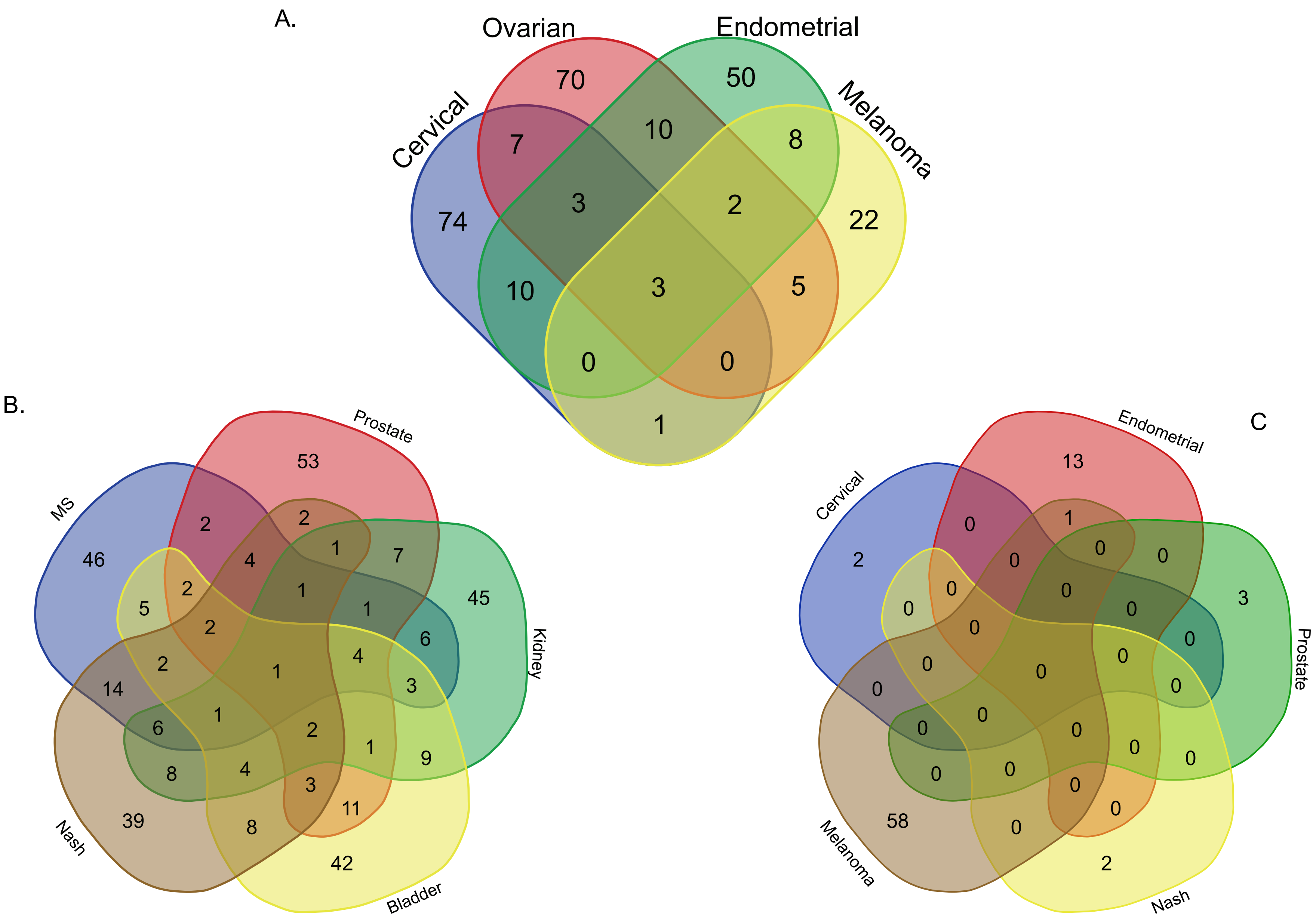

Table A. List of proteins included in the Olink® Explore 3072 analysis and their characteristics.

UniProt	Panel	LOD (pg/ml)	LLOQ (pg/ml)	ULOQ (pg/ml)	Hook (pg/mL)	Range (log10)	Intra-CV (%)	Inter-CV (%)
P31483	Cardiometabolic						9	27
P21964	Cardiometabolic	97.7	195.3	100000	800000	2.7	8	10
Q9NRD8	Cardiometabolic	97.7	195.3	100000	200000	2.7	7	31
P16860	Cardiometabolic						10	18
O60635	Cardiometabolic						7	9
O96017	Cardiometabolic						13	19
Q9UKL0	Cardiometabolic	1.5	3.1	3125	12500	3.0	7	12
Q8NHS0	Cardiometabolic						9	10
P58546	Cardiometabolic						8	8
O43854	Cardiometabolic	24.4	48.8	200000	800000	3.6	8	13
P40225	Cardiometabolic	97.7	781.3	200000	400000	2.4	9	14
Q99549	Cardiometabolic	48.8	97.7	12500	25000	2.1	8	19
P08319	Cardiometabolic						7	8
P25815	Cardiometabolic						12	15
Q8TE57	Cardiometabolic	6.1	24.4	25000	200000	3.0	9	6
Q04760	Cardiometabolic						7	9
Q9BYF1	Cardiometabolic	48.8	97.7	100000	400000	3.0	9	10
O14793	Cardiometabolic	390.6	781.3	50000	400000	1.8	10	14
Q9NWQ8	Cardiometabolic	24.4	48.8	6250	200000	2.1	11	11
Q13444	Cardiometabolic	24.4	48.8	25000	200000	2.7	7	10
P34913	Cardiometabolic	390.6	781.3	100000	200000	2.1	7	21
P09496	Cardiometabolic	3125.0	6250.0	6400000	12800000	3.0	7	18
P34947	Cardiometabolic	1562.5	3125.0	400000	800000	2.1	8	14
P55259	Cardiometabolic	3.1	6.1	12500	200000	3.3	6	9
P01375	Cardiometabolic	6.1	12.2	6250	200000	2.7	8	16
P52789	Cardiometabolic	97.7	195.3	50000	200000	2.4	9	39
P09668	Cardiometabolic						11	10
O75354	Cardiometabolic	195.3	781.3	50000	800000	1.8	8	8
Q9BWV1	Cardiometabolic						8	10

P22004	Cardiometabolic	0.001	97.7	12500	200000	2.1	13	16
P05231	Cardiometabolic	0.4	0.8	3125	12500	3.6	6	12
P46379	Cardiometabolic	390.6	781.3	200000	400000	2.4	8	10
P40818	Cardiometabolic	3125.0	6250.0	800000	800000	2.1	8	13
P62736	Cardiometabolic						7	8
P51161	Cardiometabolic						10	24
P09237	Cardiometabolic	97.7	97.7	6250	50000	1.8	6	8
Q15165	Cardiometabolic						8	8
Q92558	Cardiometabolic	6250.0	12500.0	800000	1600000	1.8	10	19
O43186	Cardiometabolic						8	13
P08670	Cardiometabolic	12500.0	12500.0	1600000	3200000	2.1	6	60
P07585	Cardiometabolic	195.3	390.6	100000	200000	2.4	7	11
Q15831	Cardiometabolic	390.6	781.3	400000	800000	2.7	10	21
P19429	Cardiometabolic	97.7	195.3	25000	200000	2.1	15	14
Q9UKP3	Cardiometabolic	195.3	781.3	100000	200000	2.1	9	18
O95988	Cardiometabolic	97.7	195.3	200000	800000	3.0	8	24
P36952	Cardiometabolic	781.3	1562.5	100000	200000	1.8	11	21
Q16619	Cardiometabolic	195.3	781.3	100000	400000	2.1	11	22
P61978	Cardiometabolic	97.7	97.7	50000	200000	2.7	8	12
P17676	Cardiometabolic						11	41
Q96N03	Cardiometabolic						8	11
Q13105	Cardiometabolic	6.1	12.2	6250	200000	2.7	8	8
O95684	Cardiometabolic	781.3	1562.5	100000	3200000	1.8	10	24
P21246	Cardiometabolic						20	17
P34998	Cardiometabolic	24.4	48.8	6250	25000	2.1	7	24
Q6UWL2	Cardiometabolic	48.8	97.7	12500	200000	2.1	8	14
Q969D9	Cardiometabolic	6.1	48.8	12500	200000	2.4	7	14
P35218	Cardiometabolic	1.5	6.1	6250	25000	3.0	7	9
P55082	Cardiometabolic	24.4	48.8	100000	800000	3.3	9	26
P17516	Cardiometabolic	390.6	781.3	100000	400000	2.1	12	17
O15354	Cardiometabolic	781.3	1562.5	100000	400000	1.8	9	11
Q12912	Cardiometabolic	48.8	97.7	12500	50000	2.1	8	12
P31997	Cardiometabolic						9	12

Q9NRV9	Cardiometabolic						8	12
Q9Y2B0	Cardiometabolic						7	10
O95183	Cardiometabolic	390.6	781.3	200000	400000	2.4	8	30
P13807	Cardiometabolic	12.2	48.8	25000	200000	2.7	8	16
P20718	Cardiometabolic						8	12
Q9H5Y7	Cardiometabolic	24.4	48.8	100000	400000	3.3	7	14
Q8NC01	Cardiometabolic	97.7	195.3	25000	400000	2.1	7	10
O75356	Cardiometabolic	48.8	97.7	100000	800000	3.0	8	8
Q96A56	Cardiometabolic	97.7	195.3	25000	200000	2.1	8	8
Q9GZM7	Cardiometabolic	1.5	3.1	6250	50000	3.3	10	6
P27352	Cardiometabolic	0.2	0.4	6250	50000	4.2	9	6
P12104	Cardiometabolic	0.4	1.5	781	6250	2.7	9	8
Q9NQX5	Cardiometabolic	6.1	12.2	3125	50000	2.4	10	6
P12724	Cardiometabolic						12	18
Q9UBU3	Cardiometabolic	24.4	48.8	50000	200000	3.0	8	13
P35754	Cardiometabolic						9	11
P41159	Cardiometabolic	12.2	48.8	12500	200000	2.4	7	11
P09382	Cardiometabolic	24.4	48.8	50000	200000	3.0	12	15
P40189	Cardiometabolic	390.6	781.3	200000	800000	2.4	7	7
Q92692	Cardiometabolic	0.1	0.1	195	781	3.3	9	6
Q15067	Cardiometabolic						9	15
Q16620	Cardiometabolic	48.8	48.8	6250	50000	2.1	10	10
P21583	Cardiometabolic	3.1	6.1	12500	50000	3.3	10	8
P31431	Cardiometabolic	1.5	3.1	3125	200000	3.0	9	6
P09417	Cardiometabolic	12.2	24.4	50000	800000	3.3	7	7
Q8WVQ1	Cardiometabolic	6.1	12.2	6250	50000	2.7	8	5
Q15846	Cardiometabolic	6.1	12.2	6250	50000	2.7	9	12
Q9UKJ0	Cardiometabolic	0.8	1.5	3125	12500	3.3	7	6
O00161	Cardiometabolic	6.1	12.2	12500	50000	3.0	12	13
Q6WN34	Cardiometabolic	195.3	390.6	50000	200000	2.1	10	7
Q92823	Cardiometabolic	6.1	12.2	6250	12500	2.7	15	16
P00568	Cardiometabolic						9	10
Q13043	Cardiometabolic	195.3	390.6	6250	50000	1.2	6	28

P09525	Cardiometabolic	97.7	195.3	25000	800000	2.1	7	14
Q05315	Cardiometabolic	390.6	781.3	50000	800000	1.8	10	8
Q9UHL4	Cardiometabolic	97.7	195.3	50000	400000	2.4	11	15
Q03154	Cardiometabolic	390.6	781.3	400000	800000	2.7	10	11
P10644	Cardiometabolic	24.4	48.8	100000	200000	3.3	12	12
O94903	Cardiometabolic	12.2	24.4	12500	50000	2.7	9	12
P16234	Cardiometabolic	12.2	48.8	6250	50000	2.1	10	6
Q9H773	Cardiometabolic	12.2	24.4	12500	50000	2.7	9	9
O14917	Cardiometabolic	24.4	48.8	50000	400000	3.0	13	10
Q9H7M9	Cardiometabolic	1.5	3.1	1563	6250	2.7	10	14
NT-proBNP	Cardiometabolic	97.7	195.3	50000	200000	2.4	11	9
P31949	Cardiometabolic	6.1	6.1	3125	12500	2.7	14	10
Q9Y4X3	Cardiometabolic						17	14
P01222	Cardiometabolic	1.5	3.1	1563	3125	2.7	11	7
P21980	Cardiometabolic	6.1	24.4	50000	200000	3.3	15	11
P21549	Cardiometabolic	390.6	781.3	1600000	12800000	3.3	9	10
Q9UMF0	Cardiometabolic	12.2	24.4	6250	50000	2.4	9	9
Q6GTS8	Cardiometabolic	48.8	97.7	100000	800000	3.0	11	13
Q9NY25	Cardiometabolic	0.1	0.4	3125	6250	3.9	9	8
Q9HBB8	Cardiometabolic	12.2	48.8	25000	200000	2.7	10	7
P16112	Cardiometabolic	12.2	24.4	50000	200000	3.3	12	9
P55285	Cardiometabolic	97.7	195.3	25000	200000	2.1	12	10
O60664	Cardiometabolic	195.3	390.6	50000	800000	2.1	12	13
P08263	Cardiometabolic	3.1	6.1	12500	400000	3.3	9	7
P52888	Cardiometabolic	6.1	12.2	6250	200000	2.7	9	5
Q969P0	Cardiometabolic	12.2	12.2	25000	200000	3.3	9	9
O75340	Cardiometabolic	1562.5	1562.5	100000	800000	1.8	8	10
Q9ULL4	Cardiometabolic	24.4	48.8	50000	200000	3.0	10	13
P41218	Cardiometabolic	390.6	781.3	800000	800000	3.0	9	10
P48357	Cardiometabolic						8	12
Q9Y286	Cardiometabolic	3.1	3.1	6250	50000	3.3	8	8
P51693	Cardiometabolic	195.3	195.3	50000	400000	2.4	12	15
O95502	Cardiometabolic	6.1	12.2	25000	50000	3.3	10	7

O75791	Cardiometabolic	1562.5	3125.0	400000	800000	2.1	8	10
Q06418	Cardiometabolic	3.1	6.1	1563	6250	2.4	9	10
Q12864	Cardiometabolic	195.3	390.6	50000	400000	2.1	14	13
Q9Y5X1	Cardiometabolic	390.6	781.3	400000	800000	2.7	9	11
Q13541	Cardiometabolic	24.4	24.4	12500	50000	2.7	8	8
Q9UHD0	Cardiometabolic	3.1	12.2	100000	400000	3.9	9	7
Q8WX77	Cardiometabolic	0.001	1.5	3125	6250	3.3	9	9
Q8WTU2	Cardiometabolic	6.1	6.1	6250	200000	3.0	7	8
P78380	Cardiometabolic	3.1	3.1	3125	12500	3.0	9	8
Q99674	Cardiometabolic	24.4	48.8	6250	50000	2.1	8	7
Q8NI22	Cardiometabolic	48.8	97.7	12500	400000	2.1	9	7
P23526	Cardiometabolic	195.3	390.6	100000	400000	2.4	8	13
Q8IW75	Cardiometabolic	24.4	97.7	6250	50000	1.8	9	14
P09601	Cardiometabolic	3.1	3.1	6250	25000	3.3	9	13
Q9BQR3	Cardiometabolic	12.2	24.4	25000	50000	3.0	8	6
Q6PJW8	Cardiometabolic	12.2	48.8	6250	50000	2.1	10	21
Q8IZP9	Cardiometabolic	12.2	48.8	12500	50000	2.4	10	8
P06858	Cardiometabolic	195.3	195.3	400000	800000	3.3	8	11
Q13158	Cardiometabolic	781.3	3125.0	800000	800000	2.4	9	10
Q9NR28	Cardiometabolic	3.1	6.1	12500	50000	3.3	12	28
Q86VZ4	Cardiometabolic	12.2	48.8	6250	200000	2.1	11	7
P35247	Cardiometabolic	6.1	12.2	6250	50000	2.7	8	5
O95544	Cardiometabolic	62.5	125.0	256000	512000	3.3	10	6
Q14956	Cardiometabolic	24.4	48.8	12500	200000	2.4	12	10
P18827	Cardiometabolic	6.1	12.2	6250	200000	2.7	14	12
P10145	Cardiometabolic	0.1	0.2	781	6250	3.6	10	15
Q53H82	Cardiometabolic	48.8	97.7	25000	200000	2.4	8	9
Q9BUD6	Cardiometabolic	24.4	48.8	25000	400000	2.7	9	8
Q16820	Cardiometabolic	97.7	195.3	25000	50000	2.1	9	9
Q9Y5K6	Cardiometabolic	6.1	12.2	12500	50000	3.0	10	9
P41236	Cardiometabolic	12.2	24.4	12500	50000	2.7	9	10
Q13275	Cardiometabolic	148.4	296.9	38000	304000	2.1	9	8
Q96LA6	Cardiometabolic	97.7	195.3	400000	800000	3.3	10	7

P19022	Cardiometabolic	390.6	781.3	200000	800000	2.4	11	9
P00797	Cardiometabolic	6.1	12.2	12500	50000	3.0	9	9
Q8N1Q1	Cardiometabolic	6.1	24.4	12500	50000	2.7	8	15
Q07108	Cardiometabolic	0.8	3.1	12500	50000	3.6	12	14
Q9UK05	Cardiometabolic	1.5	6.1	12500	50000	3.3	10	8
O95841	Cardiometabolic	97.7	195.3	100000	400000	2.7	9	7
Q9UEW3	Cardiometabolic	24.4	24.4	50000	800000	3.3	7	6
P02462	Cardiometabolic	0.8	3.1	50000	200000	4.2	10	6
P07204	Cardiometabolic	3.1	6.1	6250	50000	3.0	9	8
P01241	Cardiometabolic						7	15
A6NI73	Cardiometabolic	0.8	1.5	3125	6250	3.3	8	7
Q01973	Cardiometabolic	24.4	48.8	3125	12500	1.8	10	7
Q16773	Cardiometabolic	0.8	1.5	3125	12500	3.3	12	8
P09467	Cardiometabolic	97.7	195.3	50000	200000	2.4	8	40
P42830	Cardiometabolic	3.1	6.1	3125	6250	2.7	11	10
Q9BQB4	Cardiometabolic	6.1	6.1	25000	50000	3.6	8	11
Q76M96	Cardiometabolic	24.4	48.8	50000	800000	3.0	10	8
P19971	Cardiometabolic	390.6	781.3	200000	800000	2.4	9	17
Q92520	Cardiometabolic	3.1	6.1	6250	50000	3.0	8	7
P07711	Cardiometabolic	24.4	97.7	50000	200000	2.7	10	9
P04792	Cardiometabolic						15	28
Q99523	Cardiometabolic	24.4	24.4	50000	400000	3.3	9	8
P20711	Cardiometabolic	3.1	6.1	25000	50000	3.6	8	7
O60496	Cardiometabolic	6.1	12.2	25000	200000	3.3	10	15
P07911	Cardiometabolic	1.5	3.1	12500	100000	3.6	4	3
Q13361	Cardiometabolic	12.2	24.4	12500	100000	2.7	6	9
P00750	Cardiometabolic	0.8	3.1	25000	100000	3.9	8	8
O75326	Cardiometabolic	0.8	1.5	12500	50000	3.9	5	5
P23141	Cardiometabolic	24.4	48.8	400000	800000	3.9	5	7
P22748	Cardiometabolic	0.8	1.5	12500	100000	3.9	5	6
P55058	Cardiometabolic	6.1	12.2	25000	400000	3.3	5	11
P01130	Cardiometabolic	0.8	1.5	25000	100000	4.2	4	14
P13598	Cardiometabolic	97.7	195.3	200000	400000	3.0	6	9

Q8NBP7	Cardiometabolic	97.7	195.3	400000	800000	3.3	5	28
P15090	Cardiometabolic	48.8	195.3	50000	200000	2.4	5	5
Q76LX8	Cardiometabolic	6.1	12.2	6250	100000	2.7	6	9
P08833	Cardiometabolic	1.5	3.1	12500	100000	3.6	3	3
P33151	Cardiometabolic	195.3	390.6	200000	400000	2.7	5	8
Q16270	Cardiometabolic	12.2	24.4	12500	200000	2.7	7	8
P54760	Cardiometabolic	1.5	3.1	12500	100000	3.6	4	5
Q96AP7	Cardiometabolic	3.1	6.1	3125	12500	2.7	4	5
P32942	Cardiometabolic	1.5	1.5	3125	12500	3.3	3	4
P08118	Cardiometabolic	0.001	6.1	6250	12500	3.0	3	5
Q06141	Cardiometabolic	0.4	0.8	1563	12500	3.3	4	5
P01589	Cardiometabolic	0.02	0.0	391	3125	3.9	5	5
P07858	Cardiometabolic	12.2	24.4	12500	25000	2.7	4	4
Q9UBP4	Cardiometabolic	6.1	12.2	25000	100000	3.3	5	5
Q86U17	Cardiometabolic	3.1	6.1	12500	100000	3.3	4	14
P04066	Cardiometabolic	6.1	12.2	50000	200000	3.6	6	13
Q14767	Cardiometabolic	6.1	12.2	25000	200000	3.3	4	5
Q9NQ79	Cardiometabolic	6.1	24.4	100000	800000	3.6	6	8
O14798	Cardiometabolic	0.4	0.8	6250	12500	3.9	5	5
Q5VY43	Cardiometabolic	12.2	24.4	12500	100000	2.7	5	4
P48304	Cardiometabolic	0.8	1.5	1563	12500	3.0	4	4
P15085	Cardiometabolic	0.4	0.8	3125	12500	3.6	6	5
Q07507	Cardiometabolic	3.1	6.1	6250	400000	3.0	6	7
P17931	Cardiometabolic	12.2	48.8	12500	50000	2.4	5	5
P04275	Cardiometabolic	1.5	3.1	12500	100000	3.6	9	32
P55808	Cardiometabolic	1.5	3.1	6250	12500	3.3	6	10
Q03167	Cardiometabolic	12.2	24.4	100000	200000	3.6	5	8
P14555	Cardiometabolic						6	9
Q9Y275	Cardiometabolic	0.2	0.8	12500	50000	4.2	4	4
P08581	Cardiometabolic	6.1	12.2	12500	50000	3.0	4	6
Q9H2A7	Cardiometabolic	0.8	1.5	12500	50000	3.9	5	6
Q9UM47	Cardiometabolic	1.5	3.1	6250	50000	3.3	6	8
P07451	Cardiometabolic	1562.5	12500.0	12800000	12800000	3.0	4	5

P09619	Cardiometabolic	3.1	6.1	3125	12500	2.7	4	4
P80370	Cardiometabolic	0.8	3.1	6250	25000	3.3	4	5
Q14162	Cardiometabolic	0.8	1.5	3125	12500	3.3	5	6
Q99988	Cardiometabolic	1.5	3.1	6250	25000	3.3	5	4
P04080	Cardiometabolic	3.1	6.1	3125	12500	2.7	4	3
P02144	Cardiometabolic	0.8	0.8	195	3125	2.4	4	5
Q13822	Cardiometabolic	390.6	781.3	100000	800000	2.1	4	5
P08236	Cardiometabolic	3.1	6.1	6250	50000	3.0	4	6
Q01638	Cardiometabolic	0.8	3.1	3125	12500	3.0	4	4
Q13740	Cardiometabolic	3.1	6.1	3125	12500	2.7	4	5
P48960	Cardiometabolic	3.1	6.1	25000	200000	3.6	5	7
P17813	Cardiometabolic	1.5	3.1	3125	12500	3.0	4	4
P31146	Cardiometabolic	6.1	24.4	100000	400000	3.6	6	7
P12111	Cardiometabolic	3.1	6.1	3125	100000	2.7	4	6
P16581	Cardiometabolic	0.8	1.5	1563	3125	3.0	5	5
P15086	Cardiometabolic	1.5	3.1	6250	25000	3.3	4	5
Q15828	Cardiometabolic	12.2	24.4	3125	12500	2.1	5	5
Q9NNX6	Cardiometabolic	1.5	3.1	12500	100000	3.6	4	6
P04054	Cardiometabolic	0.4	0.8	6250	12500	3.9	4	6
Q9H1U4	Cardiometabolic	0.8	1.5	6250	25000	3.6	5	6
P19021	Cardiometabolic	97.7	195.3	200000	800000	3.0	4	6
P48745	Cardiometabolic	0.001	1.5	6250	25000	3.6	3	5
P20062	Cardiometabolic	0.8	1.5	6250	12500	3.6	5	9
O75023	Cardiometabolic	0.4	0.8	6250	25000	3.9	4	4
P18065	Cardiometabolic	12.2	24.4	50000	200000	3.3	5	7
O00584	Cardiometabolic	0.4	0.8	3125	12500	3.6	4	4
P19961	Cardiometabolic	12.2	24.4	6250	25000	2.4	8	10
Q12860	Cardiometabolic	3.1	6.1	12500	100000	3.3	5	6
Q13231	Cardiometabolic	0.4	0.8	6250	25000	3.9	5	5
P39060	Cardiometabolic	3.1	6.1	3125	12500	2.7	4	8
P25445	Cardiometabolic	0.1	0.2	6250	12500	4.5	5	6
P23284	Cardiometabolic	24.4	48.8	12500	200000	2.4	8	10
O15467	Cardiometabolic	3.1	3.1	6250	12500	3.3	4	8

Q13867	Cardiometabolic	24.4	48.8	12500	100000	2.4	4	5
Q13332	Cardiometabolic	3.1	6.1	6250	12500	3.0	5	5
P19957	Cardiometabolic	12.2	48.8	6250	12500	2.1	5	5
P35590	Cardiometabolic						4	6
P09093	Cardiometabolic	1.5	6.1	6250	25000	3.0	4	7
P46531	Cardiometabolic	0.2	0.4	6250	12500	4.2	3	4
P04746	Cardiometabolic	24.4	24.4	6250	12500	2.4	8	8
P78324	Cardiometabolic	0.4	0.8	6250	12500	3.9	5	7
P04085	Cardiometabolic	0.4	0.8	3125	12500	3.6	4	6
Q9HD89	Cardiometabolic	0.2	0.4	3125	12500	3.9	6	6
O15031	Cardiometabolic	0.8	1.5	6250	50000	3.6	4	5
P24158	Cardiometabolic	12.2	48.8	6250	100000	2.1	5	4
P05107	Cardiometabolic	1.5	3.1	6250	25000	3.3	6	8
P13987	Cardiometabolic	0.001	6.1	1563	3125	2.4	4	6
O75594	Cardiometabolic	0.8	1.5	3125	12500	3.3	5	6
Q12884	Cardiometabolic	12.2	24.4	12500	25000	2.7	5	6
P05121	Cardiometabolic	0.8	1.5	6250	25000	3.6	6	5
P00533	Cardiometabolic	3.1	6.1	6250	12500	3.0	4	6
P13686	Cardiometabolic	0.8	1.5	6250	12500	3.6	4	7
P02452	Cardiometabolic						3	4
P20160	Cardiometabolic	6.1	12.2	6250	12500	2.7	7	9
P42574	Cardiometabolic	1.5	3.1	3125	12500	3.0	5	9
P10451	Cardiometabolic	1.5	3.1	6250	12500	3.3	3	8
Q16769	Cardiometabolic	24.4	48.8	6250	50000	2.1	5	7
Q14393	Cardiometabolic	48.8	97.7	400000	800000	3.6	5	7
P42785	Cardiometabolic	24.4	24.4	6250	25000	2.4	5	6
Q8TDL5	Cardiometabolic	97.7	390.6	200000	800000	2.7	5	8
Q16663	Cardiometabolic	3.1	6.1	6250	12500	3.0	8	5
Q8N423	Cardiometabolic	0.8	1.5	6250	12500	3.6	4	6
P10586	Cardiometabolic	3.1	6.1	25000	200000	3.6	4	7
Q9Y4L1	Cardiometabolic	24.4	24.4	12500	400000	2.7	5	6
P15907	Cardiometabolic	24.4	48.8	400000	800000	3.9	6	9
Q8NHL6	Cardiometabolic	0.8	1.5	3125	12500	3.3	4	5

P43121	Cardiometabolic	24.4	48.8	25000	800000	2.7	8	7
P00740	Cardiometabolic	48.8	97.7	50000	800000	2.7	7	9
P12830	Cardiometabolic	6.1	6.1	12500	50000	3.3	9	12
P15529	Cardiometabolic	390.6	3125.0	6400000	12800000	3.3	11	13
P13591	Cardiometabolic	6.1	12.2	25000	400000	3.3	7	9
P12318	Cardiometabolic	0.2	0.4	1563	6250	3.6	10	10
Q9UBR2	Cardiometabolic	0.8	1.5	3125	6250	3.3	7	9
P18428	Cardiometabolic						11	24
Q12794	Cardiometabolic	24.4	24.4	25000	100000	3.0	7	6
P07478	Cardiometabolic	0.4	0.8	391	12500	2.7	7	6
P07359	Cardiometabolic	1.5	3.1	12500	25000	3.6	7	11
P98160	Cardiometabolic	0.4	1.5	6250	25000	3.6	5	9
P08887	Cardiometabolic	0.1	0.2	3125	6250	4.2	7	10
P59665	Cardiometabolic						12	22
P24821	Cardiometabolic	0.4	1.5	3125	25000	3.3	9	10
P16109	Cardiometabolic	0.2	0.4	3125	12500	3.9	9	11
Q14515	Cardiometabolic	6.1	12.2	12500	50000	3.0	9	9
Q86VB7	Cardiometabolic	6.1	12.2	25000	100000	3.3	8	7
O95998	Cardiometabolic	0.8	1.5	12500	25000	3.9	7	10
P20023	Cardiometabolic	0.4	0.8	12500	25000	4.2	6	8
Q9NZK5	Cardiometabolic	0.4	0.8	6250	50000	3.9	6	7
Q13508	Cardiometabolic	1.5	3.1	3125	25000	3.0	7	7
Q15485	Cardiometabolic	12.2	12.2	25000	800000	3.3	8	11
P80188	Cardiometabolic	0.8	1.5	1563	25000	3.0	8	9
P30530	Cardiometabolic	0.4	0.8	6250	25000	3.9	8	7
Q99650	Cardiometabolic	6.1	12.2	12500	25000	3.0	7	9
Q15113	Cardiometabolic	24.4	48.8	12500	25000	2.4	9	13
O14786	Cardiometabolic	6.1	12.2	12500	25000	3.0	8	8
Q96KN2	Cardiometabolic	390.6	781.3	400000	800000	2.7	8	15
Q6EMK4	Cardiometabolic	6.1	12.2	12500	50000	3.0	9	9
P19320	Cardiometabolic	1.5	3.1	12500	50000	3.6	6	8
P00441	Cardiometabolic	24.4	97.7	6250	25000	1.8	5	19
O75015	Cardiometabolic	3.1	6.1	6250	25000	3.0	7	7

P07339	Cardiometabolic	48.8	195.3	25000	50000	2.1		
Q16853	Cardiometabolic	6.1	12.2	6250	25000	2.7	8	8
P15144	Cardiometabolic	24.4	48.8	100000	800000	3.3	7	8
P08174	Cardiometabolic	0.2	0.4	3125	6250	3.9	8	9
O00533	Cardiometabolic	24.4	48.8	12500	400000	2.4	7	8
P02786	Cardiometabolic	24.4	48.8	200000	800000	3.6	7	6
P05556	Cardiometabolic	0.05	0.4	12500	25000	4.5	11	13
P10646	Cardiometabolic	0.8	1.5	6250	50000	3.6	10	13
Q9BXJ1	Cardiometabolic	0.8	1.5	6250	50000	3.6	17	19
Q9NPY3	Cardiometabolic	0.2	0.4	6250	25000	4.2	7	8
P10721	Cardiometabolic	0.4	0.8	1563	6250	3.3	8	10
P14543	Cardiometabolic	3.1	6.1	50000	200000	3.9	7	8
O95445	Cardiometabolic	6.1	6.1	12500	50000	3.3	11	12
Q96H15	Cardiometabolic	0.2	0.4	1563	6250	3.6	8	8
P08571	Cardiometabolic	12.2	24.4	200000	400000	3.9	10	14
Q99969	Cardiometabolic	6.1	24.4	25000	800000	3.0	8	12
A1L4H1	Cardiometabolic	48.8	97.7	50000	400000	2.7	9	10
Q07654	Cardiometabolic	0.2	0.4	781	3125	3.3	10	10
P35443	Cardiometabolic	6.1	24.4	50000	200000	3.3	7	9
P55774	Cardiometabolic	0.4	0.8	3125	6250	3.6	10	23
Q9Y5C1	Cardiometabolic	1.5	3.1	12500	100000	3.6	10	9
Q16627	Cardiometabolic	0.1	0.4	1563	6250	3.6	7	11
P08709	Cardiometabolic	0.8	3.1	6250	25000	3.3	6	10
P41222	Cardiometabolic	3.1	6.1	12500	50000	3.3	7	8
P06681	Cardiometabolic	48.8	97.7	200000	800000	3.3	7	11
P24592	Cardiometabolic	0.8	1.5	12500	25000	3.9	6	8
Q15582	Cardiometabolic	97.7	195.3	100000	400000	2.7	8	12
P36222	Cardiometabolic	0.4	0.8	3125	6250	3.6	6	10
Q06033	Cardiometabolic	48.8	97.7	50000	100000	2.7	7	10
Q9UGM5	Cardiometabolic	12.2	48.8	50000	200000	3.0	7	7
P49747	Cardiometabolic	1.5	3.1	12500	50000	3.6	8	9
Q92820	Cardiometabolic	195.3	390.6	50000	400000	2.1	6	7
P00915	Cardiometabolic	0.8	1.5	25000	100000	4.2	6	7

P13501	Cardiometabolic	0.4	1.5	1563	12500	3.0	10	14
P05451	Cardiometabolic	0.05	0.1	3125	6250	4.5	6	7
Q12805	Cardiometabolic	24.4	48.8	50000	100000	3.0	10	14
P03950	Cardiometabolic	0.8	1.5	781	3125	2.7	7	11
P27487	Cardiometabolic	12.2	48.8	12500	100000	2.4	6	8
P04070	Cardiometabolic	97.7	195.3	100000	400000	2.7	7	8
P05362	Cardiometabolic	0.4	0.8	6250	25000	3.9	8	8
P01034	Cardiometabolic	12.2	48.8	6250	12500	2.1	6	13
P17936	Cardiometabolic	12.2	24.4	12500	25000	2.7	8	9
P01033	Cardiometabolic	0.8	1.5	3125	12500	3.3	9	10
P14902	Cardiometabolic	390.6	1562.5	200000	400000	2.1	10	13
Q14160	Cardiometabolic	195.3	390.6	50000	200000	2.1	8	22
P12829	Cardiometabolic	3125.0	3125.0	100000	800000	1.5	11	20
Q9BY49	Cardiometabolic II						6	7
Q9NZN3	Cardiometabolic	6250.0	12500.0	800000	800000	1.8	6	27
Q96C92	Cardiometabolic	781.3	781.3	400000	800000	2.7	9	15
Q5SW79	Cardiometabolic	48.8	48.8	3125	6250	1.8	6	11
O75506	Cardiometabolic	12.2	12.2	3125	25000	2.4	6	29
Q15477	Cardiometabolic II						7	25
P04141	Cardiometabolic	12.2	24.4	12500	50000	2.7	16	18
P21817	Cardiometabolic	390.6	781.3	100000	200000	2.1	13	12
A6BM72	Cardiometabolic	97.7	97.7	25000	200000	2.4	10	10
O00291	Cardiometabolic	1562.5	3125.0	200000	400000	1.8	8	
Q8IZC4	Cardiometabolic	3125.0	6250.0	400000	800000	1.8	5	
O60701	Cardiometabolic II						9	24
O14958	Cardiometabolic	6250.0	6250.0	400000	1600000	1.8	12	21
E2RYF7	Cardiometabolic	24.4	48.8	12500	50000	2.4	6	16
Q9NVZ3	Cardiometabolic	12500.0	12500.0	400000	800000	1.5	15	25
P23634	Cardiometabolic	390.6	390.6	25000	400000	1.8		
Q9Y4C8	Cardiometabolic	390.6	390.6	100000	400000	2.4	14	18
Q9Y623	Cardiometabolic	390.6	390.6	25000	200000	1.8	14	
P54709	Cardiometabolic	781.3	781.3	100000	400000	2.1		
Q07973	Cardiometabolic	1562.5	3125.0	200000	800000	1.8	15	

P48507	Cardiometabolic	1562.5	3125.0	200000	800000	1.8	11	17
P06753	Cardiometabolic	1562.5	1562.5	200000	400000	2.1	11	12
Q04695	Cardiometabolic	390.6	781.3	200000	800000	2.4		
P25391	Cardiometabolic II							
Q15059	Cardiometabolic II						12	32
O00567	Cardiometabolic	3125.0	3125.0	200000	400000	1.8	13	37
Q9NZJ5	Cardiometabolic	3125.0	6250.0	400000	800000	1.8	17	15
P35228	Cardiometabolic	390.6	781.3	400000	800000	2.7	15	17
Q13503	Cardiometabolic	781.3	1562.5	100000	400000	1.8	9	17
P08913	Cardiometabolic	3125.0	6250.0	200000	400000	1.5	9	15
P33121	Cardiometabolic	3125.0	6250.0	200000	400000	1.5		
Q9BY32	Cardiometabolic	781.3	1562.5	50000	400000	1.5	8	21
P30049	Cardiometabolic	390.6	390.6	50000	200000	2.1	10	20
P10109	Cardiometabolic II						9	
P55011	Cardiometabolic II						8	
Q01780	Cardiometabolic	3125.0	6250.0	400000	800000	1.8	12	3
Q6UWF7	Cardiometabolic	1562.5	1562.5	200000	400000	2.1	7	14
Q9Y3B8	Cardiometabolic	97.7	195.3	12500	400000	1.8	14	
A6NCE7	Cardiometabolic	3125.0	3125.0	200000	800000	1.8	8	15
Q08499	Cardiometabolic	25000.0	50000.0	3200000	12800000	1.8	10	50
P46783	Cardiometabolic II							
Q96DA2	Cardiometabolic	3125.0	3125.0	200000	800000	1.8	13	16
P49755	Cardiometabolic	390.6	781.3	100000	200000	2.1	7	12
Q96HD9	Cardiometabolic II						10	9
B6SEH8	Cardiometabolic	195.3	781.3	100000	200000	2.1	15	33
O43734	Cardiometabolic	195.3	195.3	100000	200000	2.7	10	11
O95180	Cardiometabolic	390.6	781.3	100000	400000	2.1	12	20
Q9H2M3	Cardiometabolic	50000.0	100000.0	6400000	12800000	1.8		
P06729	Cardiometabolic	781.3	1562.5	200000	400000	2.1	10	14
Q96IW2	Cardiometabolic	6250.0	6250.0	400000	800000	1.8	12	8
P55769	Cardiometabolic II						8	12
Q9Y2W1	Cardiometabolic	3125.0	3125.0	200000	800000	1.8	9	15
O95858	Cardiometabolic	390.6	781.3	100000	200000	2.1	15	14

Q9H347	Cardiometabolic	390.6	390.6	100000	200000	2.4		
P78524	Cardiometabolic	3125.0	3125.0	200000	400000	1.8	11	6
Q14353	Cardiometabolic II						12	
Q15370	Cardiometabolic	1562.5	1562.5	100000	400000	1.8	10	32
P20929	Cardiometabolic	781.3	1562.5	200000	400000	2.1	13	8
Q9BW61	Cardiometabolic	1171.9	1171.9	300000	600000	2.4	11	11
Q5TA50	Cardiometabolic	6250.0	12500.0	400000	800000	1.5	7	17
O15305	Cardiometabolic II						11	39
P05026	Cardiometabolic	195.3	195.3	25000	200000	2.1	11	11
Q86UW2	Cardiometabolic	781.3	781.3	100000	400000	2.1	10	22
P38935	Cardiometabolic	390.6	781.3	50000	200000	1.8		
Q14088	Cardiometabolic	6250.0	12500.0	800000	800000	1.8	12	21
Q9Y2Y0	Cardiometabolic	1562.5	3125.0	200000	400000	1.8	9	17
Q8WZ42	Cardiometabolic	195.3	390.6	12500	200000	1.5	10	12
P12270	Cardiometabolic	97.7	97.7	12500	100000	2.1	14	16
O75521	Cardiometabolic	6250.0	12500.0	400000	800000	1.5	13	35
P05976	Cardiometabolic	6250.0	6250.0	200000	400000	1.5	6	14
P14415	Cardiometabolic	781.3	1562.5	100000	400000	1.8		
Q9UFP1	Cardiometabolic II						12	15
Q9BZC7	Cardiometabolic	781.3	1562.5	200000	400000	2.1	19	26
Q6NZY4	Cardiometabolic	48.8	97.7	50000	100000	2.7	18	30
Q9NYX4	Cardiometabolic	3125.0	3125.0	200000	800000	1.8	6	15
P16066	Cardiometabolic	3125.0	6250.0	200000	800000	1.5	8	9
Q99707	Cardiometabolic	781.3	781.3	50000	400000	1.8	20	10
Q8N8E3	Cardiometabolic	195.3	390.6	100000	200000	2.4	8	10
P37058	Cardiometabolic	3125.0	3125.0	200000	800000	1.8	12	25
Q92935	Cardiometabolic	390.6	390.6	100000	200000	2.4	10	14
P21673	Cardiometabolic	195.3	195.3	12500	100000	1.8	2	
O43290	Cardiometabolic	390.6	1562.5	100000	400000	1.8	11	15
Q96K76	Cardiometabolic	390.6	781.3	100000	400000	2.1		
Q13296	Cardiometabolic	1562.5	3125.0	200000	400000	1.8	23	
Q6P4F2	Cardiometabolic	195.3	195.3	12500	200000	1.8	13	18
P05000	Cardiometabolic	195.3	390.6	200000	400000	2.7	4	1

P57078	Cardiometabolic	1562.5	1562.5	200000	800000	2.1	15	31
Q9UKX7	Cardiometabolic II						11	
Q02127	Cardiometabolic	12500.0	12500.0	800000	800000	1.8	10	
Q6ZN66	Cardiometabolic II							
Q9BV94	Cardiometabolic	1562.5	3125.0	200000	800000	1.8	13	6
Q07075	Cardiometabolic	390.6	781.3	100000	400000	2.1	13	21
P23511	Cardiometabolic II						11	10
Q96LB8	Cardiometabolic	390.6	390.6	50000	100000	2.1	8	25
Q8NET8	Cardiometabolic	24.4	24.4	12500	50000	2.7	14	23
Q9NV35	Cardiometabolic	390.6	390.6	25000	200000	1.8	14	19
Q16774	Cardiometabolic II						13	3
Q16836	Cardiometabolic II							
P54296	Cardiometabolic	97.7	195.3	12500	100000	1.8	18	17
Q9BZL6	Cardiometabolic	6250.0	12500.0	800000	3200000	1.8	9	10
Q10587	Cardiometabolic	195.3	781.3	200000	800000	2.4	14	11
A6NHS7	Cardiometabolic	390.6	390.6	25000	200000	1.8	10	33
Q15018	Cardiometabolic II						9	18
O00425	Cardiometabolic	12500.0	12500.0	200000	800000	1.2		
Q9UNN8	Cardiometabolic II						8	26
Q14807	Cardiometabolic II						15	37
P35606	Cardiometabolic	195.3	195.3	6250	400000	1.5	16	32
P20382	Cardiometabolic	3125.0	3125.0	100000	800000	1.5	8	12
Q96PU4	Cardiometabolic	781.3	1562.5	100000	200000	1.8		
P00966	Cardiometabolic	781.3	3125.0	200000	400000	1.8	9	18
P48668	Cardiometabolic	390.6	390.6	25000	200000	1.8		
O00327	Cardiometabolic	3125.0	6250.0	400000	800000	1.8		
O95670	Cardiometabolic II						22	17
P50461	Cardiometabolic	1562.5	3125.0	50000	200000	1.2	24	22
Q3SXY8	Cardiometabolic	195.3	195.3	25000	400000	2.1	13	24
Q03013	Cardiometabolic	3125.0	3125.0	200000	800000	1.8	12	12
O43896	Cardiometabolic	97.7	97.7	25000	50000	2.4	9	10
P59901	Cardiometabolic II						12	
Q01484	Cardiometabolic	195.3	390.6	50000	100000	2.1	11	11

P19838	Cardiometabolic II						13	19
P22033	Cardiometabolic II						12	34
Q12986	Cardiometabolic	1562.5	3125.0	100000	400000	1.5	14	22
Q01581	Cardiometabolic	6250.0	6250.0	200000	400000	1.5	8	6
O94766	Cardiometabolic II							
Q14781	Cardiometabolic	3125.0	3125.0	400000	800000	2.1	10	
Q96A35	Cardiometabolic	3125.0	6250.0	400000	800000	1.8	11	16
Q58F21	Cardiometabolic	48.8	97.7	12500	50000	2.1	13	20
Q8NFP7	Cardiometabolic	781.3	781.3	50000	200000	1.8		
P46926	Cardiometabolic	195.3	195.3	12500	100000	1.8	9	12
Q9UBV2	Cardiometabolic	390.6	781.3	100000	400000	2.1	7	8
Q5JTV8	Cardiometabolic	6250.0	12500.0	800000	3200000	1.8	10	26
Q8ND90	Cardiometabolic	6250.0	12500.0	800000	800000	1.8	9	24
P32241	Cardiometabolic	195.3	390.6	100000	800000	2.4		
P35609	Cardiometabolic	781.3	1562.5	100000	400000	1.8	18	18
O75427	Cardiometabolic II						17	17
Q93052	Cardiometabolic II						14	15
Q86VR7	Cardiometabolic	6250.0	6250.0	200000	800000	1.5	10	
P41227	Cardiometabolic II						20	35
Q5W0V3	Cardiometabolic II						8	7
Q86VP3	Cardiometabolic	97.7	195.3	25000	200000	2.1	13	25
Q99598	Cardiometabolic II						11	20
Q13563	Cardiometabolic	781.3	1562.5	100000	200000	1.8	12	17
O75534	Cardiometabolic II						16	20
A6NDB9	Cardiometabolic II						12	19
Q5VVQ6	Cardiometabolic II						14	21
Q96EU7	Cardiometabolic II						16	26
P55010	Cardiometabolic	781.3	1562.5	100000	400000	1.8	16	27
Q9Y2L6	Cardiometabolic II						11	19
P13224	Cardiometabolic II						15	28
P0C7L1	Cardiometabolic	781.3	1562.5	200000	400000	2.1	10	
O15018	Cardiometabolic	390.6	390.6	200000	400000	2.7	14	20
P10082	Cardiometabolic II						14	18

Q7Z7H5	Cardiometabolic	1562.5	1562.5	100000	400000	1.8	11	
Q16206	Cardiometabolic	1562.5	3125.0	800000	3200000	2.4	8	27
P29536	Cardiometabolic	195.3	390.6	200000	800000	2.7	7	24
Q14324	Cardiometabolic	781.3	1562.5	100000	800000	1.8	7	21
Q96ID5	Cardiometabolic	1562.5	1562.5	100000	400000	1.8	7	15
P13929	Cardiometabolic II							
P20645	Cardiometabolic	97.7	97.7	6250	25000	1.8	9	12
P23327	Cardiometabolic	781.3	1562.5	200000	3200000	2.1	8	16
Q9H173	Cardiometabolic	390.6	781.3	50000	200000	1.8	6	9
Q9BTK6	Cardiometabolic	195.3	195.3	50000	400000	2.4	15	17
P01225	Cardiometabolic	97.7	195.3	12500	100000	1.8	6	9
Q8TER0	Cardiometabolic	1562.5	3125.0	400000	800000	2.1	12	21
Q0VD83	Cardiometabolic II						7	12
O95980	Cardiometabolic	1562.5	1562.5	50000	200000	1.5	7	5
Q13316	Cardiometabolic	3125.0	6250.0	800000	6400000	2.1	8	35
P50053	Cardiometabolic	195.3	195.3	12500	100000	1.8	6	20
Q14457	Cardiometabolic	12500.0	25000.0	800000	800000	1.5	10	14
Q99942	Cardiometabolic II						12	21
I3L3R5	Cardiometabolic	1562.5	1562.5	100000	400000	1.8	9	12
Q99807	Cardiometabolic	390.6	781.3	100000	400000	2.1	12	18
Q53T59	Cardiometabolic	3125.0	3125.0	100000	800000	1.5	8	25
Q8N668	Cardiometabolic	24.4	48.8	12500	50000	2.4	8	16
P55809	Cardiometabolic	781.3	3125.0	200000	800000	1.8	10	21
O75348	Cardiometabolic	3125.0	3125.0	200000	800000	1.8	12	35
P11532	Cardiometabolic	390.6	781.3	200000	800000	2.4	9	17
Q9Y5X3	Cardiometabolic II						5	9
P05305	Cardiometabolic II						6	15
Q8WZ75	Cardiometabolic II						6	9
Q8IVF2	Cardiometabolic	97.7	195.3	12500	50000	1.8	6	9
P35914	Cardiometabolic	781.3	1562.5	100000	400000	1.8	8	25
Q14643	Cardiometabolic	781.3	1562.5	100000	400000	1.8	13	14
Q9BQI0	Cardiometabolic	390.6	781.3	200000	800000	2.4	7	19
P36776	Cardiometabolic	781.3	781.3	50000	200000	1.8	10	28

Q9H7C9	Cardiometabolic II						8	13
O14841	Cardiometabolic II						7	24
Q8WXC3	Cardiometabolic	6.1	12.2	1563	12500	2.1	7	11
O75061	Cardiometabolic II						6	30
Q8NC42	Cardiometabolic	97.7	97.7	6250	50000	1.8	9	11
Q8TAE8	Cardiometabolic II						10	25
Q5GAN6	Cardiometabolic	195.3	195.3	12500	100000	1.8	7	13
P35520	Cardiometabolic	3125.0	3125.0	800000	800000	2.4	9	25
P30084	Cardiometabolic II						8	27
Q8WUF8	Cardiometabolic	390.6	781.3	100000	400000	2.1	8	20
O43423	Cardiometabolic	97.7	195.3	50000	400000	2.4	1	
Q13137	Cardiometabolic	195.3	195.3	50000	200000	2.4	8	15
O94979	Cardiometabolic II						7	31
Q16621	Cardiometabolic II						8	12
Q9H3K6	Cardiometabolic	195.3	195.3	25000	200000	2.1	7	11
P07098	Cardiometabolic	195.3	390.6	50000	200000	2.1	7	22
P21754	Cardiometabolic	48.8	97.7	25000	50000	2.4		
P07492	Cardiometabolic II						6	22
P20042	Cardiometabolic II						11	25
O60476	Cardiometabolic	781.3	781.3	100000	800000	2.1	5	8
Q9NYZ4	Cardiometabolic	12.2	12.2	6250	25000	2.7	6	11
Q09666	Cardiometabolic	97.7	195.3	50000	200000	2.4	7	14
Q92835	Cardiometabolic	3125.0	3125.0	200000	800000	1.8	8	12
P43487	Cardiometabolic II						7	14
Q6ZRY4	Cardiometabolic	48.8	97.7	6250	12500	1.8	6	30
P07355	Cardiometabolic	6250.0	12500.0	800000	800000	1.8	9	14
Q6YN16	Cardiometabolic II						8	12
Q9UJ70	Cardiometabolic	195.3	390.6	12500	100000	1.5	7	22
O95825	Cardiometabolic	48.8	195.3	25000	50000	2.1	7	28
Q24JP5	Cardiometabolic II							
P02458	Cardiometabolic	97.7	97.7	12500	50000	2.1	6	15
P09543	Cardiometabolic	781.3	781.3	100000	800000	2.1	6	14
P50914	Cardiometabolic	50000.0	100000.0	6400000	12800000	1.8	14	18

Q7L266	Cardiometabolic II						16	31
P01189	Cardiometabolic II							
Q8NFL0	Cardiometabolic	781.3	781.3	200000	400000	2.4	7	11
Q96DR5	Cardiometabolic	1562.5	3125.0	100000	400000	1.5	7	32
Q9HB40	Cardiometabolic	195.3	390.6	100000	400000	2.4	7	9
Q8IWT1	Cardiometabolic II						9	26
Q5FWE3	Cardiometabolic	781.3	1562.5	200000	800000	2.1	6	11
Q6UY14	Cardiometabolic II						8	13
Q9BV79	Cardiometabolic	6250.0	6250.0	400000	800000	1.8		
Q6UWR7	Cardiometabolic	12.2	24.4	6250	25000	2.4	8	22
P07942	Cardiometabolic	195.3	390.6	50000	400000	2.1	6	12
Q9NR61	Cardiometabolic II						17	5
P09681	Cardiometabolic II						13	12
P58107	Cardiometabolic	390.6	781.3	100000	400000	2.1	7	18
Q12841	Cardiometabolic II						5	9
P0DPI2	Cardiometabolic II							
P08590	Cardiometabolic	390.6	781.3	50000	800000	1.8	7	21
Q86X76	Cardiometabolic	6250.0	6250.0	400000	800000	1.8	6	13
Q96DC8	Cardiometabolic	24.4	48.8	25000	100000	2.7	4	14
Q8TCD5	Cardiometabolic	12500.0	12500.0	800000	800000	1.8	5	21
Q7Z7M9	Cardiometabolic	6250.0	6250.0	400000	800000	1.8	10	10
Q00872	Cardiometabolic	781.3	1562.5	200000	800000	2.1	7	24
Q14914	Cardiometabolic	390.6	390.6	50000	200000	2.1	5	17
Q9UBQ7	Cardiometabolic	1562.5	1562.5	200000	800000	2.1	9	18
Q9BVM4	Cardiometabolic	48.8	48.8	1563	12500	1.5	12	28
Q12982	Cardiometabolic	3125.0	3125.0	100000	800000	1.5	11	13
P33681	Cardiometabolic II						17	18
Q6UW49	Cardiometabolic	1562.5	3125.0	200000	800000	1.8	8	24
P51511	Cardiometabolic II						5	2
Q9BW04	Cardiometabolic	97.7	195.3	12500	100000	1.8		
O14933	Cardiometabolic	1562.5	1562.5	50000	200000	1.5	7	11
Q8WWV6	Cardiometabolic	6250.0	6250.0	400000	800000	1.8	6	22
P23919	Cardiometabolic	3125.0	6250.0	400000	800000	1.8	5	21

O75711	Cardiometabolic	3125.0	3125.0	100000	400000	1.5	10	12
Q6UXI7	Cardiometabolic	195.3	390.6	25000	100000	1.8	6	16
P29692	Cardiometabolic	3125.0	3125.0	400000	800000	2.1	4	12
P02008	Cardiometabolic II						7	31
Q9NQR4	Cardiometabolic II						10	32
Q9BQS7	Cardiometabolic	97.7	97.7	12500	50000	2.1	5	7
Q9Y2E5	Cardiometabolic	390.6	781.3	50000	800000	1.8	4	12
Q9H3S4	Cardiometabolic	24.4	48.8	12500	100000	2.4	5	8
O43405	Cardiometabolic	195.3	195.3	25000	200000	2.1	5	9
Q96C24	Cardiometabolic II						13	26
O60234	Cardiometabolic	781.3	781.3	50000	200000	1.8	6	23
Q7Z304	Cardiometabolic	195.3	195.3	50000	200000	2.4	5	10
P78539	Cardiometabolic	781.3	781.3	50000	200000	1.8	6	16
Q9P2J2	Cardiometabolic	97.7	97.7	25000	100000	2.4	8	19
Q8N4F0	Cardiometabolic	1562.5	3125.0	100000	200000	1.5		
P53674	Cardiometabolic	12.2	12.2	6250	25000	2.7	3	16
P16035	Cardiometabolic II						11	10
Q8N436	Cardiometabolic	12500.0	12500.0	800000	800000	1.8	8	18
Q13442	Cardiometabolic	3125.0	6250.0	200000	800000	1.5		
P14854	Cardiometabolic II						10	20
P23467	Cardiometabolic	781.3	3125.0	100000	200000	1.5	6	10
Q13428	Cardiometabolic	390.6	781.3	50000	200000	1.8	7	9
O75223	Cardiometabolic	195.3	390.6	25000	200000	1.8	9	29
O75154	Cardiometabolic	48.8	97.7	25000	200000	2.4	6	27
Q6NUS6	Cardiometabolic	1562.5	1562.5	100000	800000	1.8	6	12
Q96EM0	Cardiometabolic	781.3	781.3	50000	200000	1.8	9	30
Q96FZ7	Cardiometabolic	781.3	781.3	100000	800000	2.1	8	22
Q969H8	Cardiometabolic	97.7	195.3	12500	200000	1.8	5	29
P98161	Cardiometabolic	24.4	97.7	6250	25000	1.8	6	10
Q9BXD5	Cardiometabolic	781.3	1562.5	100000	400000	1.8	5	18
P54687	Cardiometabolic	1562.5	1562.5	100000	800000	1.8	8	10
Q9BXN1	Cardiometabolic II						8	26
P51688	Cardiometabolic	97.7	97.7	12500	50000	2.1	4	6

O14960	Cardiometabolic	6250.0	6250.0	800000	800000	2.1	9	35
P23471	Cardiometabolic	781.3	781.3	100000	200000	2.1	7	10
P32320	Cardiometabolic	1562.5	3125.0	100000	800000	1.5	9	13
P08138	Cardiometabolic	390.6	390.6	25000	100000	1.8	14	22
Q6PI73	Cardiometabolic	97.7	195.3	25000	200000	2.1	6	21
Q8NDI1	Cardiometabolic	195.3	390.6	25000	100000	1.8	8	20
P08582	Cardiometabolic	97.7	195.3	50000	200000	2.4	5	8
P52209	Cardiometabolic	6250.0	6250.0	200000	800000	1.5	8	19
O43681	Cardiometabolic	1562.5	3125.0	400000	800000	2.1	8	26
P15502	Cardiometabolic II						11	16
Q969X0	Cardiometabolic	195.3	390.6	50000	200000	2.1	6	35
Q96MK3	Cardiometabolic	390.6	390.6	100000	800000	2.4	5	14
Q8IZF2	Cardiometabolic	1562.5	3125.0	200000	800000	1.8	6	8
Q96AG4	Cardiometabolic II						12	29
Q7Z7K0	Cardiometabolic II						6	29
P07093	Cardiometabolic II						5	17
P62072	Cardiometabolic	781.3	781.3	50000	200000	1.8	10	10
P61026	Cardiometabolic II						13	16
P45954	Cardiometabolic	3125.0	3125.0	400000	800000	2.1	12	24
Q6ZMM2	Cardiometabolic	390.6	390.6	50000	100000	2.1	11	19
P05413	Cardiometabolic II						8	16
Q15388	Cardiometabolic	97.7	195.3	25000	100000	2.1	12	29
Q9UBR1	Cardiometabolic	781.3	781.3	50000	200000	1.8	14	13
P49593	Cardiometabolic	6250.0	6250.0	800000	800000	2.1	10	26
O00194	Cardiometabolic	195.3	390.6	100000	400000	2.4		
P13667	Cardiometabolic	390.6	390.6	50000	100000	2.1	8	27
P23560	Cardiometabolic	48.8	195.3	50000	100000	2.4	9	28
P30046	Cardiometabolic II						10	19
Q86TH1	Cardiometabolic	3125.0	3125.0	400000	800000	2.1	9	12
P02730	Cardiometabolic II							
P13796	Cardiometabolic	1562.5	1562.5	400000	800000	2.4	10	19
Q9Y303	Cardiometabolic	3125.0	25000.0	800000	800000	1.5	8	19
Q6H9L7	Cardiometabolic	195.3	195.3	25000	200000	2.1	11	22

P07288	Cardiometabolic	195.3	195.3	25000	200000	2.1	5	5
P16410	Cardiometabolic	97.7	97.7	25000	100000	2.4	1	
P40199	Cardiometabolic	3125.0	3125.0	400000	800000	2.1	10	15
Q8N6C8	Cardiometabolic II						5	13
Q02817	Cardiometabolic	6.1	12.2	6250	25000	2.7	7	30
P98095	Cardiometabolic	195.3	195.3	25000	100000	2.1	4	9
P02461	Cardiometabolic	97.7	195.3	25000	100000	2.1	6	10
Q6UWP8	Cardiometabolic II						7	19
Q6UVK1	Cardiometabolic	97.7	97.7	25000	100000	2.4	5	6
P39059	Cardiometabolic	195.3	195.3	12500	50000	1.8	5	10
Q9BYJ0	Cardiometabolic	195.3	195.3	25000	50000	2.1	4	15
Q9HCU0	Cardiometabolic	97.7	195.3	25000	100000	2.1	5	10
Q96CG8	Cardiometabolic	781.3	1562.5	50000	200000	1.5	8	13
Q96NZ9	Cardiometabolic II						4	12
P47972	Cardiometabolic	48.8	97.7	25000	100000	2.4	7	14
P02818	Cardiometabolic	781.3	1562.5	100000	400000	1.8	6	16
Q8N114	Cardiometabolic	390.6	390.6	25000	50000	1.8	6	9
Q6IBS0	Cardiometabolic	781.3	781.3	200000	800000	2.4		
P30405	Cardiometabolic	6250.0	6250.0	200000	800000	1.5	10	28
P32971	Cardiometabolic	12.2	24.4	12500	50000	2.7	11	11
Q9Y2Y8	Cardiometabolic	97.7	195.3	25000	200000	2.1	5	14
P35579	Cardiometabolic II							
P13727	Cardiometabolic	97.7	97.7	25000	50000	2.4	5	15
P08575	Cardiometabolic	48.8	48.8	12500	100000	2.4	7	7
O43280	Cardiometabolic	48.8	97.7	25000	800000	2.4	5	20
Q9NRR1	Cardiometabolic	390.6	390.6	25000	200000	1.8	6	12
O75339	Cardiometabolic II						9	26
Q9H2X3	Cardiometabolic	24.4	48.8	6250	25000	2.1	6	14
Q9Y646	Cardiometabolic	195.3	195.3	50000	200000	2.4	4	11
P10645	Cardiometabolic	1562.5	3125.0	200000	800000	1.8	9	22
Q04721	Cardiometabolic	48.8	97.7	6250	50000	1.8	5	6
O95965	Cardiometabolic	97.7	195.3	25000	100000	2.1	6	13
Q9Y251	Cardiometabolic II						6	33

Q8TDY8	Cardiometabolic	48.8	48.8	6250	50000	2.1	6	8
Q15063	Cardiometabolic II						9	15
P08217	Cardiometabolic	48.8	48.8	25000	100000	2.7	5	9
Q9UQP3	Cardiometabolic	50000.0	100000.0	6400000	12800000	1.8	7	8
P17900	Cardiometabolic	781.3	1562.5	50000	200000	1.5	7	17
P37837	Cardiometabolic	97.7	195.3	25000	200000	2.1	4	8
Q8WWQ8	Cardiometabolic	390.6	1562.5	100000	800000	1.8	5	9
P55000	Cardiometabolic	1562.5	1562.5	100000	800000	1.8	7	9
P12277	Cardiometabolic II						9	15
Q13510	Cardiometabolic	195.3	390.6	25000	200000	1.8	8	18
P11279	Cardiometabolic	97.7	390.6	50000	100000	2.1	7	6
P07602	Cardiometabolic	1562.5	1562.5	50000	200000	1.5	6	7
P17174	Cardiometabolic	390.6	390.6	25000	50000	1.8	8	12
P61916	Cardiometabolic	390.6	390.6	12500	50000	1.5	5	8
P19878	Inflammation	0.8	3.1	12500	25000	3.6	8	6
P40933	Inflammation	1.5	3.1	50000	400000	4.2	9	4
P11274	Inflammation	781.3	1562.5	200000	800000	2.1	7	4
P52564	Inflammation	24.4	48.8	25000	200000	2.7	9	10
Q9UN19	Inflammation	3.1	12.2	25000	200000	3.3	10	9
P24394	Inflammation	3.1	6.1	25000	100000	3.6	10	12
Q6ZUJ8	Inflammation	781.3	1562.5	400000	800000	2.4	14	13
P01730	Inflammation	97.7	97.7	400000	800000	3.6	8	9
Q13241	Inflammation	1.5	3.1	6250	25000	3.3	3	4
P35613	Inflammation	0.2	0.4	12500	50000	4.5	3	3
P50452	Inflammation	12.2	12.2	12500	50000	3.0	6	6
O43915	Inflammation	12.2	24.4	25000	100000	3.0	4	4
O00253	Inflammation	48.8	97.7	50000	400000	2.7	6	14
P10147	Inflammation	0.2	0.4	781	1563	3.3	9	5
Q92609	Inflammation						7	7
Q9GZT9	Inflammation	6.1	12.2	12500	25000	3.0	9	5
Q9Y266	Inflammation	24.4	97.7	100000	800000	3.0	4	5
Q14242	Inflammation	3.1	6.1	6250	25000	3.0	9	5
Q12918	Inflammation	0.8	1.5	3125	12500	3.3	3	2

Q3KPI0	Inflammation	0.4	0.8	1563	12500	3.3	8	6
Q9NRM6	Inflammation	24.4	24.4	25000	400000	3.0	8	8
Q01344	Inflammation	6.1	12.2	25000	400000	3.3	9	8
P02745	Inflammation	29.2	58.3	119500	239000	3.3	4	5
Q9HBG7	Inflammation	3.1	6.1	12500	50000	3.3	3	4
O94992	Inflammation	1.5	3.1	781	1563	2.4	10	7
Q08174	Inflammation	195.3	390.6	200000	800000	2.7	4	4
O60449	Inflammation	24.4	48.8	50000	400000	3.0	10	7
O15455	Inflammation	1.5	3.1	12500	100000	3.6	4	3
P22304	Inflammation	97.7	195.3	400000	800000	3.3	3	4
P43234	Inflammation	48.8	97.7	50000	400000	2.7	4	4
P14210	Inflammation	3.1	12.2	100000	200000	3.9	4	3
Q12866	Inflammation	97.7	195.3	100000	800000	2.7	5	6
P51671	Inflammation	1.5	3.1	25000	200000	3.9	3	4
P42701	Inflammation	97.7	195.3	100000	800000	2.7	8	17
P09874	Inflammation	48.8	97.7	50000	200000	2.7	7	14
Q5R372	Inflammation	48.8	48.8	25000	200000	2.7	10	19
Q13459	Inflammation						10	19
O95760	Inflammation	6.1	24.4	6250	25000	2.4	8	58
P14784	Inflammation	97.7	390.6	200000	800000	2.7	10	37
Q8NHJ6	Inflammation	97.7	195.3	100000	800000	2.7	8	15
P01584	Inflammation	0.4	1.5	25000	100000	4.2	10	20
P60568	Inflammation	0.4	0.8	6250	25000	3.9	7	20
O76038	Inflammation	97.7	390.6	200000	800000	2.7	9	11
O95715	Inflammation						8	15
Q8N6P7	Inflammation	0.4	1.5	3125	12500	3.3	8	22
P22301	Inflammation	6.1	24.4	200000	800000	3.9	10	18
Q9UPV0	Inflammation	12.2	48.8	12500	25000	2.4	7	18
P28838	Inflammation						12	20
O60934	Inflammation						11	9
P57771	Inflammation						18	14
Q03426	Inflammation	390.6	781.3	400000	800000	2.7	11	12
O14904	Inflammation	3.1	6.1	25000	100000	3.6	9	11

Q9Y478	Inflammation	48.8	97.7	25000	100000	2.4	7	21
P20809	Inflammation	6.1	12.2	6250	100000	2.7	9	9
P05412	Inflammation	390.6	781.3	100000	3200000	2.1	11	14
O43707	Inflammation	390.6	781.3	100000	400000	2.1	7	14
Q96PD4	Inflammation	1.5	6.1	6250	25000	3.0	8	18
P05112	Inflammation	0.8	1.5	6250	25000	3.6	7	33
P35225	Inflammation	1.5	6.1	100000	800000	4.2	9	22
Q96AX2	Inflammation	12.2	24.4	12500	100000	2.7	9	19
Q9NYY1	Inflammation	24.4	97.7	25000	800000	2.4	7	16
Q96P31	Inflammation	48.8	97.7	50000	800000	2.7	8	18
Q9NP70	Inflammation	12.2	24.4	25000	400000	3.0	9	15
Q13007	Inflammation	24.4	48.8	12500	25000	2.4	14	16
Q9HCU5	Inflammation	390.6	781.3	200000	800000	2.4	9	12
Q8WV07	Inflammation	12.2	24.4	50000	100000	3.3	8	17
Q9Y2J8	Inflammation	48.8	97.7	400000	800000	3.6	8	15
Q9Y3P8	Inflammation	0.04	4.5	18500	37000	3.6	8	16
Q8IU57	Inflammation	0.8	1.5	25000	100000	4.2	11	5
P30838	Inflammation	0.8	3.1	3125	25000	3.0	10	10
O14867	Inflammation	12.2	24.4	25000	200000	3.0	8	7
P19801	Inflammation	97.7	195.3	100000	800000	2.7	10	21
Q16552	Inflammation	6.1	12.2	50000	100000	3.6	7	7
Q7Z739	Inflammation	12.2	24.4	12500	100000	2.7	9	32
O60575	Inflammation						10	6
P26951	Inflammation						7	28
Q8TAD2	Inflammation	3.1	6.1	6250	25000	3.0	8	14
Q9P0M4	Inflammation	12.2	24.4	50000	100000	3.3	10	14
Q7Z6M3	Inflammation						7	21
Q8TCS8	Inflammation						9	11
Q5T4W7	Inflammation	0.8	1.5	6250	25000	3.6	9	9
Q99748	Inflammation	0.8	1.5	3125	25000	3.3	15	31
P48061	Inflammation						8	17
Q04759	Inflammation						13	18
Q12933	Inflammation						9	4

P42768	Inflammation						8	31
O95379	Inflammation						11	16
Q13219	Inflammation	195.3	390.6	50000	200000	2.1	12	15
Q13574	Inflammation	3.1	6.1	3125	25000	2.7	8	13
P63241	Inflammation						11	10
O43736	Inflammation						7	9
O60542	Inflammation	3.1	6.1	12500	100000	3.3	11	5
P13693	Inflammation	1562.5	6250.0	400000	800000	1.8	16	19
P09038	Inflammation	12.2	12.2	12500	400000	3.0	4	6
Q9Y5A7	Inflammation	3.1	6.1	12500	25000	3.3	8	12
Q6UXK5	Inflammation	12.2	24.4	25000	400000	3.0	6	17
P01375	Inflammation	1.5	3.1	6250	100000	3.3	9	15
Q13651	Inflammation	1.5	6.1	400000	800000	4.8	7	17
Q96RJ3	Inflammation	12.2	24.4	6250	100000	2.4	9	13
P27540	Inflammation						8	14
Q969V3	Inflammation						6	17
Q9UHF4	Inflammation	0.8	1.5	12500	400000	3.9	8	13
Q06520	Inflammation	195.3	390.6	200000	400000	2.7	10	8
Q6UB28	Inflammation	0.4	0.8	12500	25000	4.2	8	9
Q0Z7S8	Inflammation	3.1	12.2	12500	25000	3.0	10	7
O60880	Inflammation	6.1	12.2	6250	25000	2.7	8	5
Q12968	Inflammation	6.1	24.4	3125	12500	2.1	8	9
P78362	Inflammation	3.1	6.1	1563	12500	2.4	5	11
P01903	Inflammation	390.6	781.3	400000	800000	2.7	10	9
P78410	Inflammation	48.8	97.7	200000	800000	3.3	10	7
O43521-2	Inflammation						9	8
P01583	Inflammation	1.5	3.1	3148	12594	3.0	8	47
P01579	Inflammation	1.5	6.1	12500	50000	3.3	15	22
Q05084	Inflammation	48.8	195.3	100000	400000	2.7	8	16
Q7L8A9	Inflammation	195.3	390.6	100000	400000	2.4	6	9
P05113	Inflammation	6.1	12.2	100000	400000	3.9	10	21
O43597	Inflammation	24.4	97.7	50000	400000	2.7	9	15
Q13261	Inflammation	6.1	12.2	3125	25000	2.4	7	23

P12034	Inflammation	3.1	6.1	50000	100000	3.9	11	19
Q92844	Inflammation	48.8	195.3	100000	400000	2.7	8	15
O95644	Inflammation	390.6	781.3	400000	800000	2.7	10	9
P09919	Inflammation	97.7	390.6	400000	800000	3.0	9	14
Q9BXJ7	Inflammation						8	19
Q13291	Inflammation	781.3	1562.5	400000	800000	2.4	14	17
P51617	Inflammation	781.3	3125.0	400000	800000	2.1	11	14
Q12778	Inflammation						8	8
Q14435	Inflammation	781.3	1562.5	400000	800000	2.4	11	14
P30048	Inflammation						8	12
P32456	Inflammation	390.6	781.3	400000	800000	2.7	12	20
P01591	Inflammation						13	19
P55957	Inflammation	781.3	3125.0	400000	3200000	2.1	11	11
Q12765	Inflammation	48.8	97.7	50000	400000	2.7	9	12
Q6ZMH5	Inflammation						10	9
Q8N8S7	Inflammation	48.8	48.8	6250	25000	2.1	10	20
Q9Y6K9	Inflammation	3.1	12.2	12500	25000	3.0	13	14
P18564	Inflammation	1.5	3.1	6250	25000	3.3	8	14
P58294	Inflammation	3.1	12.2	12500	25000	3.0	9	5
Q9HB29	Inflammation	0.8	1.5	6250	100000	3.6	8	16
P05231	Inflammation	0.2	0.4	3125	25000	3.9	10	14
P12872	Inflammation	390.6	781.3	200000	400000	2.4	12	10
Q96DB9	Inflammation	6.1	12.2	3125	100000	2.4	8	13
Q96LC7	Inflammation	12.2	48.8	200000	800000	3.6	8	4
O75475	Inflammation						9	8
P19474	Inflammation	24.4	97.7	400000	800000	3.6	12	9
B1AKI9	Inflammation	97.7	195.3	200000	400000	3.0	10	10
P13232	Inflammation	1.5	3.1	6250	12500	3.3	12	9
P13747	Inflammation	12500.0	25000.0	1600000	3200000	1.8	6	4
Q9UNK0	Inflammation	12.2	48.8	12500	25000	2.4	4	13
P33241	Inflammation	97.7	97.7	25000	400000	2.4	10	8
Q8WTT0	Inflammation	24.4	48.8	25000	400000	2.7	8	4
P13725	Inflammation	0.2	0.4	781	3125	3.3	12	6

Q8IVG5	Inflammation	195.3	390.6	200000	800000	2.7	7	8
Q8TD46	Inflammation	6.1	12.2	25000	100000	3.3	7	7
Q9UHC6	Inflammation	12.2	24.4	12500	400000	2.7	10	10
P50995	Inflammation	1562.5	3125.0	800000	800000	2.4	7	9
Q6DN72	Inflammation	97.7	195.3	200000	800000	3.0	10	5
P23582	Inflammation	12.2	24.4	12500	25000	2.7	11	8
Q8NDB2	Inflammation	195.3	390.6	200000	400000	2.7	9	16
Q01151	Inflammation	1.5	3.1	12500	25000	3.6	9	6
P45984	Inflammation						12	13
Q9NRJ3	Inflammation	97.7	195.3	100000	400000	2.7	11	12
Q9NZN5	Inflammation	781.3	3125.0	400000	800000	2.1	8	9
Q9HD26	Inflammation	12.2	24.4	12500	25000	2.7	7	16
P28827	Inflammation	97.7	195.3	200000	800000	3.0	11	11
P29965	Inflammation	0.8	1.5	6250	12500	3.6	4	4
P16455	Inflammation	195.3	390.6	100000	400000	2.4	12	13
Q9BT73	Inflammation	390.6	781.3	200000	400000	2.4	9	10
Q8N608	Inflammation	6.1	12.2	25000	200000	3.3	9	9
P28845	Inflammation						9	7
Q9UNE0	Inflammation	0.4	0.8	781	12500	3.0	9	5
P20849	Inflammation	6.1	12.2	12500	25000	3.0	11	6
Q9HCM2	Inflammation	24.4	97.7	50000	400000	2.7	12	6
P01588	Inflammation	3.9	7.8	1000	4000	2.1	11	11
P23229	Inflammation						9	7
P80098	Inflammation	0.4	0.8	781	25000	3.0	10	8
O76036	Inflammation	3.1	6.1	12500	25000	3.3	9	6
P01374	Inflammation	0.8	1.5	3125	12500	3.3	8	4
P42575	Inflammation	195.3	390.6	200000	800000	2.7	7	5
P24071	Inflammation	0.8	3.1	12500	25000	3.6	9	9
Q9NWZ3	Inflammation	390.6	1562.5	400000	800000	2.4	9	14
Q6UXB4	Inflammation	3.1	6.1	50000	200000	3.9	10	6
P37235	Inflammation	48.8	97.7	50000	200000	2.7	4	5
Q9Y258	Inflammation	97.7	781.3	400000	800000	2.7	15	17
Q9UKX5	Inflammation	97.7	390.6	200000	800000	2.7	10	15

Q9H0P0	Inflammation	97.7	195.3	200000	800000	3.0	9	7
P08727	Inflammation	48.8	97.7	100000	400000	3.0	8	9
P20340	Inflammation	48.8	97.7	200000	800000	3.3	8	8
Q9UIB8	Inflammation	24.4	48.8	12500	200000	2.4	9	7
P78310	Inflammation	3.1	3.1	1563	12500	2.7	12	7
P32970	Inflammation	12.2	48.8	100000	200000	3.3	10	6
Q29983_Q2	Inflammation	6.1	12.2	12500	25000	3.0	10	8
O14788	Inflammation	12.2	24.4	50000	200000	3.3	11	7
Q9UDT6	Inflammation	195.3	195.3	50000	800000	2.4	13	20
Q9C035	Inflammation						10	17
P26022	Inflammation						9	7
Q07065	Inflammation						3	6
P80162	Inflammation						6	13
P20783	Inflammation	0.4	0.8	3125	12500	3.6	15	7
Q14773	Inflammation	781.3	1562.5	400000	800000	2.4	9	5
Q16698	Inflammation						11	11
P50591	Inflammation	24.4	97.7	25000	50000	2.4	4	4
Q8WXI8	Inflammation	3.1	6.1	12500	25000	3.3	10	8
O94856	Inflammation						3	2
P49771	Inflammation	0.4	0.8	1563	12500	3.3	6	4
Q14005	Inflammation	1.5	3.1	3125	12500	3.0	3	6
Q15517	Inflammation	390.6	781.3	400000	800000	2.7	4	4
O15169	Inflammation	781.3	1562.5	400000	800000	2.4	6	9
Q9NQ25	Inflammation	195.3	390.6	200000	800000	2.7	10	6
Q9UMR7	Inflammation	0.8	3.1	6250	25000	3.3	9	7
O43561	Inflammation	48.8	97.7	25000	100000	2.4	7	8
P10145	Inflammation	0.1	0.2	1563	6250	3.9	3	4
Q96SB3	Inflammation	12.2	24.4	25000	800000	3.0	7	12
P41217	Inflammation	6.1	12.2	3125	25000	2.4	10	6
P14317	Inflammation	195.3	195.3	25000	400000	2.1	8	8
Q9BZW8	Inflammation	1.5	3.1	6250	25000	3.3	4	5
Q16719	Inflammation	48.8	48.8	400000	800000	3.9	3	11
O00273	Inflammation	0.8	3.1	6250	50000	3.3	5	6

Q13478	Inflammation	0.2	0.8	6250	25000	3.9	3	3
O75077	Inflammation	12.2	24.4	50000	200000	3.3	5	3
Q9UQV4	Inflammation	12.2	24.4	25000	200000	3.0	5	4
P24001	Inflammation	0.8	3.1	12500	25000	3.6	9	7
P36959	Inflammation						4	4
P30203	Inflammation	1.5	1.5	6250	25000	3.6	4	4
P20273	Inflammation	3.1	6.1	50000	200000	3.9	3	3
Q6UXB2	Inflammation	3.1	6.1	12500	50000	3.3	4	4
P68106	Inflammation	390.6	781.3	200000	400000	2.4	5	26
P12544	Inflammation	390.6	390.6	800000	800000	3.3	5	5
O95971	Inflammation	1.5	3.1	12500	25000	3.6	3	7
P43489	Inflammation	0.8	1.5	6250	12500	3.6	5	4
P01137	Inflammation						4	4
Q15661	Inflammation	6.1	12.2	100000	200000	3.9	4	3
Q04637	Inflammation	12.2	24.4	25000	200000	3.0	6	10
P48023	Inflammation	0.2	0.2	6250	12500	4.5	4	4
P40259	Inflammation	0.8	1.5	6250	25000	3.6	4	4
Q03431	Inflammation	3.1	3.1	6250	25000	3.3	7	8
Q9Y6Q6	Inflammation	0.8	1.5	6250	12500	3.6	4	4
Q96LA5	Inflammation	24.4	48.8	50000	200000	3.0	5	3
Q9BXN2	Inflammation	3.1	6.1	3125	12500	2.7	3	5
Q9H4D0	Inflammation	6.1	6.1	25000	200000	3.6	5	3
P29460	Inflammation	0.4	0.8	3125	12500	3.6	5	4
P42702	Inflammation	6.1	48.8	12500	400000	2.4	12	7
Q99616	Inflammation	0.8	6.1	3125	6250	2.7	5	5
P00813	Inflammation	781.3	1562.5	3200000	12800000	3.3	4	3
P30044	Inflammation	1562.5	3125.0	800000	800000	2.4	4	25
O60884	Inflammation	97.7	390.6	400000	800000	3.0	6	16
P15692	Inflammation	0.8	1.5	6250	12500	3.6	7	7
O43508	Inflammation	12.2	24.4	100000	400000	3.6	5	4
O15444	Inflammation	3.1	24.4	25000	100000	3.0	5	6
P10144	Inflammation						12	7
P01135	Inflammation	0.2	0.4	1563	25000	3.6	12	7

P78556	Inflammation	3.1	6.1	6250	25000	3.0	4	5
P03956	Inflammation	0.8	1.5	3125	12500	3.3	4	5
P49763	Inflammation	0.4	0.8	6250	12500	3.9	4	3
Q9BY76	Inflammation	12.2	24.4	25000	50000	3.0	7	4
O95750	Inflammation	3.1	6.1	12500	50000	3.3	6	4
O14836	Inflammation	6.1	24.4	50000	200000	3.3	8	4
P46109	Inflammation	6.1	12.2	25000	100000	3.3	6	8
Q03405	Inflammation	0.2	0.4	1563	6250	3.6	6	4
O43598	Inflammation	6.1	12.2	6250	12500	2.7	7	8
Q9HCB6	Inflammation	48.8	97.7	100000	200000	3.0	5	4
Q9NQ30	Inflammation	3.1	6.1	6250	25000	3.0	8	5
Q16651	Inflammation	0.4	0.8	6250	12500	3.9	8	3
O00468	Inflammation	6.1	12.2	6250	12500	2.7	7	5
P29350	Inflammation	781.3	1562.5	100000	200000	1.8	6	7
Q07325	Inflammation	0.05	0.1	6250	12500	4.8	7	5
Q9UII2	Inflammation	195.3	390.6	50000	200000	2.1	8	7
Q9H008	Inflammation	0.8	1.5	12500	25000	3.9	8	5
P19876	Inflammation	12.2	48.8	50000	200000	3.0	10	9
Q6UWV6	Inflammation	0.8	1.5	12500	200000	3.9	6	4
P09341	Inflammation	0.4	0.8	6250	12500	3.9	8	8
Q9H3U7	Inflammation	24.4	48.8	50000	200000	3.0	7	3
Q92583	Inflammation	0.4	0.8	781	3125	3.0	7	14
Q99538	Inflammation	0.8	3.1	6250	50000	3.3	9	5
O00182	Inflammation	48.8	97.7	50000	200000	2.7	7	4
Q03403	Inflammation	0.8	1.5	3125	12500	3.3	8	6
P53634	Inflammation	24.4	48.8	50000	200000	3.0	8	12
Q5ZPR3	Inflammation	97.7	195.3	400000	800000	3.3	7	4
P55773	Inflammation	6.1	12.2	6250	25000	2.7	6	8
P25116	Inflammation	48.8	195.3	200000	400000	3.0	8	5
Q9NZC2	Inflammation						6	10
P34896	Inflammation	12.2	24.4	100000	400000	3.6	8	5
Q15389	Inflammation	12.2	24.4	100000	200000	3.6	6	3
O00626	Inflammation	6.1	6.1	3125	12500	2.7	14	20

O75888	Inflammation	3.1	6.1	25000	100000	3.6	5	5
P47712	Inflammation	781.3	1562.5	400000	800000	2.4	6	7
Q15166	Inflammation	390.6	390.6	200000	800000	2.7	5	9
Q14118	Inflammation	3.1	6.1	6250	12500	3.0	7	4
Q9BZZ2	Inflammation	12.2	24.4	100000	200000	3.6	7	4
Q8NFT8	Inflammation	1.5	3.1	25000	100000	3.9	5	4
Q99685	Inflammation	1562.5	12500.0	3200000	12800000	2.4	9	9
O00585	Inflammation	24.4	48.8	200000	800000	3.6	8	4
P19256	Inflammation	0.4	0.8	3125	12500	3.6	6	4
P09326	Inflammation	6.1	12.2	100000	200000	3.9	6	4
Q5KU26	Inflammation	1.5	3.1	6250	25000	3.3	7	6
Q6GTX8	Inflammation	0.1	0.2	391	3125	3.3	7	4
P09603	Inflammation	0.05	0.2	3125	6250	4.2	4	3
P51888	Inflammation	6.1	12.2	12500	200000	3.0	6	4
P16422	Inflammation	0.4	0.4	6250	25000	4.2	7	7
P01133	Inflammation	0.1	0.2	781	1563	3.6	13	9
P02778	Inflammation	0.2	6.1	6250	25000	3.0	7	9
Q92484	Inflammation	6.1	12.2	100000	200000	3.9	6	4
Q7KYR7	Inflammation	3.1	6.1	6250	25000	3.0	7	4
O43291	Inflammation	6.1	12.2	25000	50000	3.3	8	6
Q9Y6N7	Inflammation	3.1	6.1	100000	200000	4.2	6	4
Q8WU39	Inflammation	0.4	0.8	6250	50000	3.9	7	2
P35625	Inflammation	390.6	781.3	200000	400000	2.4	13	13
O43639	Inflammation						5	10
O76096	Inflammation	0.4	0.8	6250	12500	3.9	7	5
O00339	Inflammation	24.4	48.8	25000	50000	2.7	8	3
O75462	Inflammation	390.6	781.3	100000	800000	2.1	6	7
Q9UJU6	Inflammation	24.4	97.7	50000	200000	2.7	10	10
Q15109	Inflammation	0.8	3.1	6250	25000	3.3	7	5
Q08334	Inflammation	1.5	6.1	3125	12500	2.7	8	3
Q9HC38	Inflammation	0.8	3.1	6250	25000	3.3	7	4
P21709	Inflammation	3.1	6.1	50000	200000	3.9	7	3
P27930	Inflammation	0.8	1.5	6250	12500	3.6	8	3

Q9UJA9	Inflammation	6.1	6.1	3125	12500	2.7	4	3
Q9NZV1	Inflammation	1.5	3.1	6250	50000	3.3	8	4
P15260	Inflammation	1.5	3.1	3125	12500	3.0	7	4
P21860	Inflammation	6.1	12.2	12500	50000	3.0	7	5
P09238	Inflammation	0.2	0.4	6250	12500	4.2	7	7
Q9NR12	Inflammation	24.4	48.8	25000	50000	2.7	7	11
P11684	Inflammation	390.6	781.3	400000	800000	2.7	6	4
P18510	Inflammation	0.2	0.4	1563	12500	3.6	6	3
Q14210	Inflammation	6.1	12.2	12500	200000	3.0	9	5
Q14116	Inflammation	0.1	0.2	6250	12500	4.5	5	4
P13236	Inflammation	0.2	0.4	1563	200000	3.6	7	5
Q99435	Inflammation	12.2	24.4	50000	100000	3.3	6	2
P36941	Inflammation	0.4	0.8	6250	12500	3.9	7	5
P30613	Inflammation	3.1	6.1	50000	200000	3.9	6	7
P55145	Inflammation	3.1	6.1	12500	25000	3.3	7	14
O00241	Inflammation	1.5	1.5	6250	25000	3.6	4	4
P29279	Inflammation	24.4	48.8	25000	200000	2.7	4	5
P0DMV8	Inflammation	48.8	97.7	50000	200000	2.7	6	4
O00300	Inflammation	0.1	0.2	6250	25000	4.5	7	3
Q8WXD2	Inflammation	12.2	24.4	25000	200000	3.0	7	4
O14773	Inflammation	1.5	3.1	12500	100000	3.6	6	3
Q96KG7	Inflammation	87.9	175.8	90000	360000	2.7	5	5
Q4KMG0	Inflammation	12.2	24.4	50000	200000	3.3	6	4
O95866	Inflammation	0.8	1.5	1563	3125	3.0	12	14
P56470	Inflammation	1.5	6.1	6250	25000	3.0	5	4
O75563	Inflammation	24.4	48.8	50000	200000	3.0	9	11
P01127	Inflammation	6.1	6.1	6250	12500	3.0	10	11
Q96PL1	Inflammation	3.1	12.2	50000	200000	3.6	8	11
O95633	Inflammation	12.2	12.2	6250	25000	2.7	6	5
Q9UKU9	Inflammation	781.3	1562.5	6400000	12800000	3.6	9	8
Q9BYZ8	Inflammation	48.8	97.7	200000	400000	3.3	7	9
P24387	Inflammation	24.4	97.7	100000	800000	3.0	7	5
Q99983	Inflammation	195.3	390.6	50000	200000	2.1	7	3

Q13232	Inflammation	6.1	12.2	6250	25000	2.7	8	5
Q9Y3D6	Inflammation	195.3	781.3	800000	800000	3.0	9	12
P19883	Inflammation	12.2	24.4	25000	50000	3.0	5	6
P15291	Inflammation	24.4	48.8	100000	200000	3.3	10	6
P12532	Inflammation	48.8	97.7	400000	800000	3.6	7	15
Q9UHX3	Inflammation	0.8	1.5	6250	25000	3.6	7	5
Q9NQ76	Inflammation	24.4	48.8	12500	25000	2.4	8	3
Q6UXH1	Inflammation	3.1	6.1	12500	200000	3.3	8	3
Q99895	Inflammation	3.1	6.1	12500	50000	3.3	7	9
P07148	Inflammation	3.1	6.1	100000	200000	4.2	8	5
Q16363	Inflammation	12.2	24.4	100000	200000	3.6	7	5
Q92956	Inflammation	0.8	1.5	6250	25000	3.6	8	4
P22466	Inflammation	24.4	48.8	100000	200000	3.3	8	7
Q8TEU8	Inflammation	3.1	6.1	6250	25000	3.0	5	4
Q8IYS5	Inflammation	3.1	6.1	6250	200000	3.0	7	5
O00175	Inflammation	0.8	0.8	3125	6250	3.6	8	10
P25942	Inflammation	0.1	0.2	3125	12500	4.2	9	8
P54317	Inflammation	3.1	6.1	25000	200000	3.6	6	4
Q9BU40	Inflammation	390.6	390.6	100000	200000	2.4	8	6
Q8N907	Inflammation II						10	
Q04609	Inflammation II	6250.0	6250.0	400000	800000	1.8	12	14
P62834	Inflammation II						11	16
P10070	Inflammation II	195.3	195.3	12500	25000	1.8		
P61328	Inflammation II	24.4	48.8	3125	25000	1.8		
O00206	Inflammation II							
P46013	Inflammation II	48.8	97.7	12500	100000	2.1	13	24
P24864	Inflammation II	390.6	781.3	50000	400000	1.8		
Q9H832	Inflammation II	390.6	781.3	100000	800000	2.1	13	21
P85299	Inflammation II	3125.0	6250.0	400000	800000	1.8	13	0.3
P49715	Inflammation II							
Q9Y4C1	Inflammation II	1562.5	3125.0	200000	800000	1.8		
Q9NP95	Inflammation II	3125.0	6250.0	800000	800000	2.1	6	18
P06401	Inflammation II	781.3	781.3	200000	800000	2.4	10	9

Q6UXL0	Inflammation II	390.6	781.3	200000	800000	2.4		
Q9NP85	Inflammation II	390.6	781.3	50000	800000	1.8	6	20
P15531	Inflammation II	3125.0	3125.0	200000	400000	1.8		
Q6UXM1	Inflammation II							
Q05329	Inflammation II	1562.5	6250.0	400000	800000	1.8		
P09693	Inflammation II	390.6	781.3	50000	400000	1.8	10	13
P24928	Inflammation II						6	
Q09472	Inflammation II	390.6	390.6	12500	25000	1.5	3	
Q9HBE5	Inflammation II	781.3	781.3	50000	400000	1.8		
P43378	Inflammation II	1562.5	6250.0	400000	800000	1.8		
Q92185	Inflammation II	390.6	1562.5	200000	800000	2.1	14	20
Q9BY41	Inflammation II							
P10767	Inflammation II	1562.5	3125.0	400000	800000	2.1	13	16
Q01201	Inflammation II						12	7
P41273	Inflammation II						12	17
P03372	Inflammation II						8	29
Q9UPW0	Inflammation II	195.3	390.6	25000	800000	1.8	12	12
P25490	Inflammation II							
Q6R327	Inflammation II						4	17
Q13190	Inflammation II						7	10
Q6UXZ4	Inflammation II	48.8	97.7	12500	100000	2.1	15	14
Q9NQI0	Inflammation II	195.3	195.3	12500	100000	1.8		
Q8WX93	Inflammation II	781.3	1562.5	100000	400000	1.8	10	27
Q16665	Inflammation II	12.2	24.4	1563	12500	1.8	8	20
P15927	Inflammation II							
Q99665	Inflammation II	3125.0	3125.0	400000	800000	2.1	2	28
O75365	Inflammation II						8	10
P24530	Inflammation II	48.8	97.7	6250	25000	1.8	8	20
Q04837	Inflammation II						12	16
O00401	Inflammation II							
Q9NZS2	Inflammation II	6.1	12.2	3125	25000	2.4	9	13
Q9NWV8	Inflammation II						4	
Q7Z698	Inflammation II						14	21

Q9Y2I7	Inflammation II						8	12
Q9NRR2	Inflammation II	390.6	390.6	25000	800000	1.8	19	
Q96EB6	Inflammation II	195.3	390.6	25000	200000	1.8	9	19
Q12888	Inflammation II	390.6	1562.5	400000	800000	2.4	11	18
Q9NY59	Inflammation II	3125.0	6250.0	400000	800000	1.8	10	
Q8WVV4	Inflammation II	390.6	390.6	100000	200000	2.4	17	24
Q13114-2	Inflammation II						10	13
Q6EBC2	Inflammation II	781.3	1562.5	200000	800000	2.1	8	9
Q14511	Inflammation II	195.3	195.3	12500	50000	1.8	11	
Q8NHP1	Inflammation II						15	23
O95429	Inflammation II						13	14
P36897	Inflammation II	390.6	390.6	50000	200000	2.1	1	
Q8IX19	Inflammation II	24.4	48.8	6250	25000	2.1	21	28
Q6B9Z1	Inflammation II	24.4	48.8	12500	50000	2.4	13	25
Q9Y3D3	Inflammation II	3125.0	6250.0	400000	800000	1.8		
Q8IYW5	Inflammation II							
Q96MM7	Inflammation II						9	24
Q9UHN6	Inflammation II	48.8	195.3	25000	200000	2.1	12	14
Q5TBC7	Inflammation II	48.8	97.7	12500	50000	2.1	13	15
P09923	Inflammation II	390.6	781.3	25000	100000	1.5	10	36
Q96D71	Inflammation II						14	16
Q92574	Inflammation II	3125.0	6250.0	400000	800000	1.8	14	21
P98170	Inflammation II	3125.0	3125.0	400000	800000	2.1	8	15
P36551	Inflammation II	6250.0	6250.0	800000	800000	2.1	9	17
O00148	Inflammation II	97.7	390.6	25000	200000	1.8	5	16
O43583	Inflammation II	390.6	781.3	25000	100000	1.5	11	21
P48730	Inflammation II						12	36
Q9NS62	Inflammation II	1562.5	3125.0	200000	800000	1.8	7	35
Q8NI17	Inflammation II	1562.5	3125.0	200000	800000	1.8	11	16
Q93062	Inflammation II						10	16
Q9UIK4	Inflammation II	1562.5	1562.5	100000	800000	1.8	18	22
Q8NBK3	Inflammation II	3125.0	6250.0	400000	800000	1.8	8	17
P35219	Inflammation II	3125.0	6250.0	800000	6400000	2.1		

O60447	Inflammation II	195.3	390.6	100000	200000	2.4	9	15
Q8IV38	Inflammation II	781.3	1562.5	100000	400000	1.8	11	23
P54274	Inflammation II	781.3	1562.5	50000	400000	1.5	12	
Q9GZN4	Inflammation II						12	12
O75173	Inflammation II	781.3	781.3	100000	800000	2.1	13	25
P17643	Inflammation II	6250.0	12500.0	800000	800000	1.8	10	16
Q15465	Inflammation II							
Q7Z5L3	Inflammation II	1562.5	1562.5	100000	400000	1.8	10	16
Q96EP0	Inflammation II						11	15
Q9NQ66	Inflammation II	6250.0	6250.0	400000	800000	1.8		
Q5JS54	Inflammation II						12	12
O43184	Inflammation II	390.6	781.3	50000	800000	1.8	14	14
Q9UHA7	Inflammation II	195.3	195.3	25000	200000	2.1	21	
Q15697	Inflammation II	390.6	390.6	25000	200000	1.8	9	18
P17050	Inflammation II	3125.0	6250.0	400000	800000	1.8	13	17
Q9H0U9	Inflammation II	390.6	1562.5	100000	800000	1.8	9	17
P56645	Inflammation II						16	37
P16671	Inflammation II	781.3	781.3	50000	400000	1.8	9	14
P26436	Inflammation II	12.2	48.8	6250	50000	2.1	9	24
Q96PX8	Inflammation II	195.3	195.3	25000	200000	2.1	11	19
P29536	Inflammation II						17	23
P14902	Inflammation II	195.3	390.6	100000	400000	2.4	13	17
Q14160	Inflammation II						16	26
Q16718	Inflammation II	781.3	1562.5	200000	800000	2.1	16	17
Q15223	Inflammation II						9	13
P11487	Inflammation II	1562.5	1562.5	100000	800000	1.8		
P48546	Inflammation II	24.4	48.8	12500	50000	2.4		
Q8WV28	Inflammation II	12500.0	12500.0	800000	800000	1.8	16	18
O60500	Inflammation II	781.3	1562.5	100000	400000	1.8	12	17
Q96PL5	Inflammation II	12.2	24.4	1563	6250	1.8	13	15
Q86UE4	Inflammation II	781.3	781.3	50000	100000	1.8		
P20701	Inflammation II						13	30
P52630	Inflammation II	781.3	1562.5	100000	800000	1.8	11	24

O43320	Inflammation II	6250.0	6250.0	400000	800000	1.8	17	14
P81534	Inflammation II						6	26
Q9Y2X7	Inflammation II						15	17
Q15399	Inflammation II	12.2	24.4	12500	100000	2.7	14	17
Q9H3T2	Inflammation II	781.3	1562.5	200000	800000	2.1	10	14
P55211	Inflammation II							
Q99584	Inflammation II	3.1	12.2	12500	400000	3.0	10	31
Q9UHI8	Inflammation II	390.6	781.3	50000	100000	1.8	14	13
P23743	Inflammation II	6250.0	12500.0	800000	800000	1.8	8	18
Q99062	Inflammation II	97.7	195.3	12500	100000	1.8	10	11
O75688	Inflammation II	1562.5	3125.0	200000	800000	1.8	9	17
Q5T2W1	Inflammation II	3125.0	6250.0	400000	800000	1.8	15	18
P49757	Inflammation II	390.6	1562.5	100000	800000	1.8	8	30
P11234	Inflammation II						21	
P06213	Inflammation II	1562.5	1562.5	50000	400000	1.5	6	6
O95157	Inflammation II	390.6	390.6	25000	200000	1.8	9	12
O60437	Inflammation II	48.8	97.7	6250	50000	1.8	9	14
Q9H7Z7	Inflammation II						17	27
O15400	Inflammation II						10	19
Q02880	Inflammation II	781.3	1562.5	50000	400000	1.5	11	29
P06730	Inflammation II	195.3	195.3	25000	100000	2.1	11	37
Q15762	Inflammation II						15	21
Q9BV40	Inflammation II	1562.5	3125.0	400000	3200000	2.1	12	30
P49765	Inflammation II						9	12
P32927	Inflammation II	97.7	97.7	12500	100000	2.1	9	22
Q5QGZ9	Inflammation II						8	9
Q9NZH8	Inflammation II						16	16
Q16643	Inflammation II	24.4	48.8	3125	800000	1.8	9	17
Q9H171	Inflammation II	6250.0	12500.0	800000	800000	1.8	9	10
P09564	Inflammation II	781.3	781.3	200000	800000	2.4	15	11
O60238	Inflammation II	195.3	390.6	12500	100000	1.5	15	18
P24666	Inflammation II	6250.0	6250.0	400000	800000	1.8		
Q96PU5	Inflammation II						8	21

O94916	Inflammation II						12	18
O95835	Inflammation II						14	38
Q8N556	Inflammation II						6	23
P17301	Inflammation II						8	14
Q8NEU8	Inflammation II	195.3	390.6	50000	800000	2.1		
Q02223	Inflammation II						5	12
Q5JS37	Inflammation II						5	7
Q6QNK2	Inflammation II	48.8	97.7	12500	50000	2.1	5	10
Q8N8U9	Inflammation II	24.4	48.8	25000	100000	2.7	5	11
P20908	Inflammation II	12.2	12.2	6250	50000	2.7	6	11
Q9BX67	Inflammation II	48.8	97.7	12500	100000	2.1	14	33
P22303	Inflammation II	24.4	48.8	12500	100000	2.4	6	16
Q9BUH6	Inflammation II	97.7	195.3	25000	100000	2.1	5	9
O76074	Inflammation II	1562.5	3125.0	200000	800000	1.8	5	27
Q9BRK3	Inflammation II	195.3	390.6	25000	200000	1.8	5	14
Q9H7Y0	Inflammation II						6	10
P01037	Inflammation II	390.6	781.3	50000	400000	1.8	6	32
P04083	Inflammation II						7	16
Q8TEA8	Inflammation II	781.3	781.3	50000	800000	1.8		
Q9ULI3	Inflammation II	390.6	781.3	50000	400000	1.8	5	9
P30040	Inflammation II	97.7	97.7	6250	12500	1.8		
Q96QR1	Inflammation II						6	13
O75347	Inflammation II	24.4	48.8	6250	50000	2.1	5	27
Q9UGN4	Inflammation II	48.8	97.7	6250	50000	1.8	5	9
Q8NDA2	Inflammation II	390.6	781.3	100000	400000	2.1	5	21
P06280	Inflammation II						5	11
Q6P5S2	Inflammation II	12.2	48.8	6250	25000	2.1	5	18
Q16378	Inflammation II						7	18
P61457	Inflammation II						5	12
Q9UI42	Inflammation II						6	13
P27348	Inflammation II	390.6	390.6	25000	200000	1.8	6	29
Q99574	Inflammation II	195.3	195.3	6250	50000	1.5	8	12
P06132	Inflammation II	390.6	390.6	25000	200000	1.8	6	16

Q5TDH0	Inflammation II	195.3	390.6	50000	100000	2.1	6	17
Q9HCU4	Inflammation II						5	8
P40197	Inflammation II	390.6	781.3	50000	200000	1.8	5	16
P04406	Inflammation II						6	21
Q9NS98	Inflammation II						6	13
P00325	Inflammation II	3125.0	6250.0	400000	800000	1.8	9	19
P55083	Inflammation II	390.6	781.3	50000	200000	1.8	5	6
Q8TDQ7	Inflammation II	390.6	390.6	12500	50000	1.5	6	15
Q9H939	Inflammation II	1562.5	3125.0	200000	800000	1.8	10	37
Q9BUN1	Inflammation II						5	7
O75190	Inflammation II	781.3	3125.0	200000	800000	1.8	6	27
P04090	Inflammation II	781.3	3125.0	800000	800000	2.4	20	24
O95393	Inflammation II	781.3	781.3	50000	100000	1.8	7	11
O43399	Inflammation II	781.3	781.3	50000	800000	1.8	6	37
Q96HD1	Inflammation II	195.3	195.3	25000	200000	2.1	5	11
Q02952	Inflammation II	781.3	1562.5	100000	1600000	1.8	8	10
Q4VCS5	Inflammation II	12.2	24.4	3125	12500	2.1	7	20
P10912	Inflammation II						5	10
Q9BXJ0	Inflammation II	1562.5	3125.0	200000	1600000	1.8	5	9
P30101	Inflammation II						11	18
P0C862	Inflammation II	195.3	195.3	12500	400000	1.8	4	18
P30047	Inflammation II						5	8
Q15276	Inflammation II	48.8	195.3	25000	100000	2.1	7	14
O14745	Inflammation II						14	33
Q6FHJ7	Inflammation II						5	10
Q58EX2	Inflammation II	195.3	390.6	25000	100000	1.8	10	15
Q9HB71	Inflammation II	390.6	781.3	50000	200000	1.8	6	35
P14091	Inflammation II	97.7	97.7	50000	200000	2.7	6	13
O60279	Inflammation II	195.3	390.6	25000	100000	1.8	4	16
P20155	Inflammation II	48.8	48.8	6250	25000	2.1	5	10
A6NC86	Inflammation II	390.6	781.3	50000	200000	1.8	11	16
A2VDF0	Inflammation II	48.8	195.3	12500	100000	1.8	5	22
P49862	Inflammation II						6	14

P09529	Inflammation II	390.6	390.6	25000	100000	1.8	7	18
P25686	Inflammation II	195.3	390.6	25000	100000	1.8	6	11
Q86SX6	Inflammation II	48.8	97.7	6250	50000	1.8		
P22455	Inflammation II	390.6	781.3	50000	100000	1.8	5	13
Q53FA7	Inflammation II	6.1	24.4	6250	50000	2.4	5	10
O96007	Inflammation II	1562.5	3125.0	200000	800000	1.8	7	11
Q8NHV1	Inflammation II	12.2	48.8	12500	100000	2.4	7	11
Q9NQ48	Inflammation II	24.4	97.7	50000	200000	2.7	9	24
Q96AJ9	Inflammation II	195.3	195.3	12500	25000	1.8	9	13
Q6UX06	Inflammation II	781.3	1562.5	400000	800000	2.4	6	34
Q96A49	Inflammation II						9	24
P01210	Inflammation II	3125.0	3125.0	200000	400000	1.8	6	8
Q92619	Inflammation II	1562.5	1562.5	400000	800000	2.4	9	16
P21854	Inflammation II	97.7	195.3	12500	100000	1.8	6	14
P52758	Inflammation II	48.8	97.7	3125	12500	1.5	5	10
Q9Y4D1	Inflammation II	6250.0	6250.0	400000	800000	1.8	9	27
Q9UK23	Inflammation II	12.2	48.8	6250	50000	2.1	5	9
Q8IUZ5	Inflammation II	3125.0	3125.0	400000	800000	2.1	8	14
Q9NRS6	Inflammation II	195.3	195.3	12500	100000	1.8	11	16
Q5VTT5	Inflammation II	12.2	24.4	6250	50000	2.4	5	36
P37840	Inflammation II							
P07311	Inflammation II	12.2	48.8	3125	12500	1.8	5	15
Q04323	Inflammation II						10	21
Q6P589	Inflammation II						8	22
P32321	Inflammation II	195.3	390.6	25000	100000	1.8	6	24
P56192	Inflammation II	1562.5	3125.0	400000	800000	2.1	6	16
O15335	Inflammation II	195.3	390.6	25000	100000	1.8	5	18
Q9P2T1	Inflammation II	97.7	195.3	25000	100000	2.1	6	15
P35611	Inflammation II						10	21
P54764	Inflammation II						6	9
Q13976	Inflammation II						5	31
Q8N0X7	Inflammation II	97.7	390.6	50000	400000	2.1	6	16
Q6GMV3	Inflammation II	390.6	390.6	50000	800000	2.1	7	26

P50502	Inflammation II	390.6	390.6	50000	800000	2.1	5	9
P32119	Inflammation II						8	12
P0DML2	Inflammation II	97.7	97.7	12500	50000	2.1		
P40925	Inflammation II	1562.5	6250.0	400000	800000	1.8	4	8
P10599	Inflammation II	48.8	97.7	6250	12500	1.8	24	23
Q92686	Inflammation II	48.8	48.8	12500	800000	2.4	7	28
Q08830	Inflammation II	390.6	390.6	100000	200000	2.4	4	27
Q13790	Inflammation II						6	20
P07333	Inflammation II	97.7	195.3	12500	50000	1.8	5	9
P00390	Inflammation II	195.3	390.6	25000	100000	1.8	5	6
P0DJD7	Inflammation II	12.2	24.4	25000	100000	3.0	4	9
P02748	Inflammation II						19	28
P17927	Inflammation II	6.1	6.1	6250	50000	3.0	6	9
P12821	Inflammation II	390.6	1562.5	200000	800000	2.1	7	9
Q6UXB8	Inflammation II						6	6
P22897	Inflammation II	24.4	48.8	12500	200000	2.4	5	9
P16233	Inflammation II	6.1	12.2	12500	50000	3.0	5	13
P50552	Inflammation II	6250.0	12500.0	800000	800000	1.8	9	36
P04180	Inflammation II	390.6	781.3	50000	400000	1.8	5	9
O43493	Inflammation II	97.7	195.3	12500	800000	1.8	7	10
O00602	Inflammation II						11	22
Q93091	Inflammation II	6.1	24.4	3125	12500	2.1	6	7
P12955	Inflammation II	195.3	390.6	25000	200000	1.8	6	9
Q0ZGT2	Inflammation II	48.8	97.7	25000	200000	2.4		
P20061	Inflammation II	12.2	24.4	6250	50000	2.4	5	10
P02652	Inflammation II							
P04278	Inflammation II	1562.5	3125.0	400000	800000	2.1	3	10
P00742	Inflammation II	97.7	195.3	12500	50000	1.8	8	9
P07307	Inflammation II	195.3	390.6	25000	200000	1.8	8	14
P08294	Inflammation II	3.1	12.2	1563	12500	2.1	4	11
Q86YW5	Inflammation II	97.7	390.6	25000	200000	1.8	8	29
P09172	Inflammation II	97.7	195.3	100000	400000	2.7	4	32
P27169	Inflammation II						7	11

P16442	Inflammation II	12.2	48.8	50000	200000	3.0	8	9
O95497	Inflammation II	24.4	48.8	6250	50000	2.1	4	20
Q04756	Inflammation II	48.8	195.3	12500	50000	1.8	4	7
P06276	Inflammation II						4	12
Q9BXR6	Inflammation II						6	15
P54108	Inflammation II	390.6	390.6	25000	50000	1.8	3	7
P06396	Inflammation II	1562.5	3125.0	200000	800000	1.8	4	8
P0DN86	Inflammation II						13	
P26927	Inflammation II	97.7	195.3	25000	200000	2.1	4	14
P04040	Inflammation II						7	11
P34096	Inflammation II	781.3	781.3	50000	200000	1.8	6	9
P07998	Inflammation II						10	11
Q01459	Inflammation II	195.3	195.3	12500	50000	1.8	4	5
P06744	Inflammation II	195.3	390.6	50000	200000	2.1	7	16
P04114	Inflammation II						9	29
P01019	Inflammation II	1562.5	3125.0	200000	800000	1.8	6	31
P0DUB6_P0	Inflammation II	195.3	195.3	12500	50000	1.8	6	12
P02765	Inflammation II						5	14
O14791	Inflammation II	97.7	97.7	25000	100000	2.4	4	33
P07358	Inflammation II						12	14
P36980	Inflammation II	195.3	390.6	25000	100000	1.8	8	16
P08185	Inflammation II	390.6	781.3	200000	800000	2.4	5	8
P05543	Inflammation II	195.3	390.6	25000	200000	1.8	7	8
P36955	Inflammation II	195.3	390.6	50000	800000	2.1	3	8
Q96PD5	Inflammation II	1562.5	3125.0	400000	800000	2.1	4	6
Q08380	Inflammation II	97.7	195.3	12500	50000	1.8	7	11
P10909	Inflammation II	781.3	1562.5	100000	800000	1.8	5	8
P61769	Inflammation II	48.8	97.7	6250	25000	1.8	6	5
P43652	Inflammation II	97.7	195.3	50000	200000	2.4	6	7
Q92496	Inflammation II	48.8	97.7	12500	50000	2.1	4	12
P35542	Inflammation II						5	9
P0DOY2	Inflammation II	6.1	12.2	781	50000	1.8	4	5
P00748	Inflammation II	97.7	195.3	25000	200000	2.1	9	23

P11226	Inflammation II	1.5	12.2	3125	25000	2.4	5	27
P00734	Inflammation II						6	7
P02649	Inflammation II						10	17
Q9Y5Y7	Inflammation II	3.1	6.1	1563	12500	2.4	6	11
P00746	Inflammation II	12.2	24.4	12500	50000	2.7	7	6
P07225	Inflammation II	195.3	390.6	50000	200000	2.1	5	7
P03952	Inflammation II	195.3	390.6	25000	200000	1.8	5	7
Q16610	Inflammation II	6250.0	6250.0	400000	800000	1.8	7	15
O75882-2	Inflammation II	195.3	390.6	50000	200000	2.1	5	6
P05154	Inflammation II						5	14
O00391	Inflammation II	390.6	390.6	12500	50000	1.5	6	7
P02775	Inflammation II	24.4	48.8	12500	100000	2.4	5	28
Q96IY4	Inflammation II	195.3	390.6	25000	200000	1.8	6	9
P20742	Inflammation II	390.6	390.6	100000	800000	2.4	4	6
P06727	Inflammation II	781.3	1562.5	100000	800000	1.8	8	16
P09871	Inflammation II	195.3	390.6	50000	200000	2.1	6	7
P02776	Inflammation II						11	28
P05452	Inflammation II	97.7	195.3	25000	200000	2.1	5	6
Q14624	Inflammation II	390.6	781.3	50000	200000	1.8	4	14
P05546	Inflammation II	3125.0	3125.0	400000	800000	2.1	5	11
P02647	Inflammation II						8	14
Q15848	Inflammation II	781.3	781.3	25000	50000	1.5	7	18
P10643	Inflammation II	24.4	48.8	12500	100000	2.4	9	6
P08519	Inflammation II	24.4	48.8	3125	50000	1.8		
P00736	Inflammation II	390.6	1562.5	100000	200000	1.8	5	6
P05090	Inflammation II	781.3	1562.5	50000	200000	1.5	7	17
P14151	Inflammation II	3.1	6.1	1563	6250	2.4	4	6
P43251	Inflammation II	12.2	24.4	3125	25000	2.1	5	7
Q9NZP8	Inflammation II	12.2	24.4	6250	50000	2.4	5	7
P02763	Inflammation II	24.4	48.8	6250	25000	2.1	6	5
P19827	Inflammation II	195.3	195.3	25000	200000	2.1	6	8
P02654	Inflammation II	195.3	390.6	100000	800000	2.4	9	16
P00751	Inflammation II	781.3	1562.5	100000	800000	1.8	6	12

P01009	Inflammation II	3125.0	6250.0	400000	800000	1.8	3	4
P49908	Inflammation II	97.7	195.3	25000	100000	2.1	15	19
P01031	Inflammation II	24.4	48.8	3125	12500	1.8	7	7
P02750	Inflammation II	97.7	97.7	6250	25000	1.8	5	6
P04196	Inflammation II	97.7	195.3	25000	200000	2.1	6	9
P00747	Inflammation II	97.7	195.3	50000	200000	2.4	4	11
P27918	Inflammation II	195.3	390.6	25000	200000	1.8	4	7
P02787	Inflammation II						5	5
P02751	Inflammation II						4	23
P02766	Inflammation II	195.3	195.3	12500	50000	1.8	5	5
P05160	Inflammation II	390.6	781.3	25000	100000	1.5	6	10
P02774	Inflammation II	97.7	390.6	25000	200000	1.8	4	7
P08697	Inflammation II	390.6	781.3	100000	400000	2.1	4	6
P02671	Inflammation II	781.3	1562.5	200000	800000	2.1	6	19
P05155	Inflammation II	390.6	390.6	25000	200000	1.8	4	7
P01008	Inflammation II						4	4
P01024	Inflammation II						7	40
O43866	Inflammation II	24.4	24.4	12500	50000	2.7	4	13
P29622	Inflammation II						5	6
P02743	Inflammation II	195.3	390.6	25000	100000	1.8	4	10
P01011	Inflammation II						3	6
P04217	Inflammation II	48.8	195.3	12500	200000	1.8	4	6
P08603	Inflammation II						5	8
P03951	Inflammation II	12.2	48.8	12500	50000	2.4	6	9
P05156	Inflammation II	195.3	390.6	50000	200000	2.1	5	9
A1E959	Neurology	195.3	781.3	100000	200000	2.1	8	14
P06748	Neurology	24.4	48.8	12500	200000	2.4	8	10
Q9NRG1	Neurology	3125.0	6250.0	400000	800000	1.8	10	11
P58417	Neurology	48.8	195.3	100000	200000	2.7	8	13
Q9H3R2	Neurology	6.1	12.2	6250	25000	2.7	9	19
Q6UX27	Neurology	48.8	97.7	6250	25000	1.8	9	9
Q15043	Neurology	97.7	390.6	12500	50000	1.5	9	19
Q9HA65	Neurology	195.3	390.6	50000	200000	2.1	8	12

O60242	Neurology	12.2	24.4	100000	800000	3.6	7	6
Q06323	Neurology	97.7	195.3	50000	200000	2.4	8	8
Q92765	Neurology	97.7	195.3	25000	50000	2.1	7	5
P16278	Neurology	195.3	390.6	100000	800000	2.4	8	10
Q9H1C3	Neurology	3125.0	6250.0	1600000	3200000	2.4	9	9
Q9NR71	Neurology	97.7	195.3	25000	200000	2.1	11	6
O95994	Neurology	1562.5	1562.5	100000	800000	1.8	21	13
O60609	Neurology	24.4	48.8	12500	50000	2.4	9	8
Q9HD42	Neurology						5	10
Q16762	Neurology	6250.0	6250.0	800000	1600000	2.1	11	13
P61244	Neurology	97.7	390.6	25000	100000	1.8	7	19
P09211	Neurology	12500.0	12500.0	400000	1600000	1.5	8	17
Q6UXK2	Neurology	24.4	48.8	25000	200000	2.7	6	15
P28325	Neurology	24.4	24.4	6250	25000	2.4	6	6
P78423	Neurology	24.4	48.8	6250	12500	2.1	8	8
P29466	Neurology	97.7	195.3	50000	100000	2.4	8	21
P07196	Neurology	390.6	781.3	200000	800000	2.4	12	12
Q9Y2W6	Neurology	6.1	12.2	6250	25000	2.7	6	13
O94985	Neurology	390.6	1562.5	200000	800000	2.1	10	11
Q6UWL6	Neurology	97.7	195.3	50000	200000	2.4	8	14
Q9UHV9	Neurology	97.7	195.3	100000	800000	2.7	7	23
P50135	Neurology	12.2	48.8	25000	50000	2.7	7	6
Q9H3S3	Neurology	3.1	6.1	6250	50000	3.0	8	5
O14763	Neurology	0.8	1.5	3125	12500	3.3	10	7
O15496	Neurology	1.5	3.1	3125	12500	3.0	9	6
Q9Y6Y9	Neurology	6250.0	12500.0	1600000	3200000	2.1	9	6
O00559	Neurology	97.7	390.6	50000	200000	2.1	8	6
P11464	Neurology	6.1	12.2	3125	12500	2.4	8	7
P15509	Neurology	781.3	1562.5	400000	800000	2.4	9	8
P42892	Neurology						13	17
Q96NZ8	Neurology	12.2	24.4	50000	200000	3.3	10	5
P78333	Neurology	12.2	48.8	25000	50000	2.7	10	6
P78560	Neurology						10	6

O14618	Neurology	781.3	1562.5	400000	800000	2.4	9	10
P07306	Neurology	195.3	390.6	100000	800000	2.4	7	6
P13500	Neurology	6.1	12.2	1563	6250	2.1	9	8
P02533	Neurology	97.7	390.6	200000	800000	2.7	6	18
Q9NRW1	Neurology	195.3	781.3	50000	200000	1.8	8	11
Q6ZMC9	Neurology	97.7	195.3	100000	200000	2.7	6	15
P53582	Neurology	781.3	1562.5	200000	800000	2.1	7	23
Q6ZMJ4	Neurology	48.8	195.3	200000	800000	3.0	8	14
Q8IU54	Neurology	6.1	12.2	25000	100000	3.3	9	16
Q8N2G4	Neurology	195.3	390.6	100000	400000	2.4	6	12
P20936	Neurology	6250.0	6250.0	3200000	6400000	2.7	9	15
P48047	Neurology	48.8	48.8	100000	800000	3.3	6	20
Q9BXS1	Neurology	195.3	781.3	200000	800000	2.4	12	20
Q4LE39	Neurology	12.2	24.4	12500	200000	2.7	6	15
P05455	Neurology	390.6	1562.5	200000	800000	2.1	6	10
Q6P1M0	Neurology	24.4	24.4	100000	200000	3.6	8	18
Q9NXA8	Neurology	1562.5	1562.5	200000	400000	2.1	12	24
Q6PIL6	Neurology	48.8	48.8	12500	200000	2.4	8	8
P53985	Neurology	781.3	1562.5	400000	800000	2.4	13	9
Q86VW0	Neurology	3125.0	3125.0	400000	800000	2.1	14	6
P49023	Neurology						7	14
P51531	Neurology	97.7	195.3	50000	200000	2.4	7	12
P08758	Neurology						10	17
P15018	Neurology	3.1	6.1	3125	100000	2.7	6	9
P13385	Neurology	390.6	781.3	100000	200000	2.1	8	34
P15336	Neurology	1406.3	2812.5	360000	720000	2.1	8	16
O96013	Neurology	390.6	1562.5	200000	800000	2.1	12	15
Q96JP9	Neurology	48.8	97.7	50000	200000	2.7	8	10
P45452	Neurology	24.4	48.8	100000	400000	3.3	8	13
P10636	Neurology	1562.5	1562.5	50000	200000	1.5	11	22
O14579	Neurology	781.3	1562.5	400000	800000	2.4	8	12
P09769	Neurology	390.6	390.6	100000	400000	2.4	8	19
P53539	Neurology	195.3	390.6	200000	800000	2.7	6	22

Q9HAW4	Neurology						17	32
O95466	Neurology	3125.0	3125.0	200000	800000	1.8	11	19
Q96FQ6	Neurology	97.7	390.6	25000	400000	1.8	13	13
O00399	Neurology	781.3	3125.0	200000	800000	1.8	5	14
Q8WTV0	Neurology	1562.5	3125.0	400000	800000	2.1	7	14
P22676	Neurology	195.3	781.3	200000	400000	2.4	9	19
Q9UBB4	Neurology	195.3	390.6	50000	200000	2.1	9	13
Q9H2W6	Neurology						11	21
Q07812	Neurology	14.2	28.3	29000	116000	3.0	9	21
Q16864	Neurology						7	16
Q9BW30	Neurology	195.3	390.6	25000	100000	1.8	8	10
P23276	Neurology	1562.5	3125.0	200000	800000	1.8	6	8
Q92597	Neurology	48.8	195.3	100000	400000	2.7	9	15
P26440	Neurology	3125.0	3125.0	400000	800000	2.1	10	12
Q9UM07	Neurology						9	14
Q9UJY5	Neurology						11	11
P14625	Neurology						7	11
Q5JZY3	Neurology	97.7	195.3	100000	800000	2.7	8	15
Q8WUW1	Neurology	24.4	48.8	100000	200000	3.3	9	13
Q15126	Neurology	3125.0	3125.0	800000	6400000	2.4	17	21
P30519	Neurology	24.4	48.8	100000	200000	3.3	8	10
Q86Z14	Neurology	1562.5	1562.5	200000	800000	2.1	12	22
Q9H0C8	Neurology	781.3	1562.5	400000	800000	2.4	10	17
O14523	Neurology	25000.0	50000.0	6400000	12800000	2.1	5	
O94813	Neurology	97.7	390.6	100000	200000	2.4	7	11
P13647	Neurology						11	17
Q9NS71	Neurology	1562.5	3125.0	800000	3200000	2.4	6	26
Q13308	Neurology	390.6	390.6	100000	200000	2.4	7	11
Q9UKV5	Neurology	97.7	195.3	100000	400000	2.7	7	21
Q9NSK7	Neurology	48.8	195.3	100000	200000	2.7	7	18
P29474	Neurology	24.4	48.8	100000	200000	3.3	10	14
P60484	Neurology						10	11
Q15633	Neurology	97.7	195.3	100000	200000	2.7	7	19

Q9Y5P4	Neurology						10	13
Q9UPY6	Neurology						6	9
P01375	Neurology	1.5	6.1	6250	200000	3.0	10	16
P29475	Neurology	6.1	6.1	25000	50000	3.6	11	21
P51452	Neurology						9	10
P41208	Neurology	3125.0	3125.0	200000	800000	1.8	11	20
O60240	Neurology						6	9
Q9Y6E0	Neurology	24.4	195.3	50000	200000	2.4	5	18
P30039	Neurology	390.6	781.3	400000	800000	2.7	7	12
Q03393	Neurology	24.4	48.8	25000	200000	2.7	8	11
Q13426	Neurology	390.6	781.3	100000	200000	2.1	7	11
P39905	Neurology	0.4	0.8	1563	12500	3.3	7	22
Q9UK53	Neurology	48.8	97.7	100000	200000	3.0	8	15
Q02643	Neurology	3125.0	3125.0	400000	800000	2.1	9	17
Q9Y2V2	Neurology	781.3	1562.5	100000	400000	1.8	11	17
Q9UJ72	Neurology	48.8	97.7	25000	200000	2.4	8	19
Q8NBI3	Neurology	97.7	195.3	50000	200000	2.4	8	9
P50453	Neurology	1562.5	3125.0	400000	800000	2.1	8	7
P52798	Neurology	6.1	6.1	3125	12500	2.7	7	5
Q92932	Neurology	12.2	48.8	25000	50000	2.7	9	7
Q02083	Neurology	97.7	195.3	100000	800000	2.7	7	7
Q9H5V8	Neurology	48.8	97.7	6250	12500	1.8	7	6
Q16740	Neurology	48.8	195.3	100000	800000	2.7	7	10
P52943	Neurology	781.3	1562.5	200000	800000	2.1	7	6
P36269	Neurology	781.3	1562.5	400000	800000	2.4	9	6
Q9Y6D9	Neurology						9	5
P63098	Neurology						8	7
O95256	Neurology	12.2	12.2	6250	50000	2.7	9	5
Q9HAN9	Neurology						7	7
Q96IU4	Neurology						8	16
O43155	Neurology	12.2	48.8	25000	100000	2.7	9	8
P07741	Neurology	390.6	781.3	400000	800000	2.7	10	6
O00220	Neurology	6.1	12.2	6250	25000	2.7	10	6

Q96PP9	Neurology	1562.5	6250.0	800000	3200000	2.1	9	10
Q92917	Neurology	3125.0	6250.0	400000	3200000	1.8	8	10
Q8IUN9	Neurology	12.2	48.8	12500	50000	2.4	7	8
Q96B36	Neurology	546.9	1093.8	70000	280000	1.8	9	16
Q92851	Neurology	97.7	195.3	50000	200000	2.4	7	12
O95817	Neurology	97.7	97.7	50000	200000	2.7	9	6
O95630	Neurology	24.4	48.8	25000	200000	2.7	10	7
Q2TAL6	Neurology	97.7	195.3	25000	50000	2.1	8	6
Q9UBT3	Neurology						9	5
O00308	Neurology	97.7	390.6	400000	800000	3.0	11	10
P09466	Neurology	46.4	92.8	47500	380000	2.7	10	13
Q08629	Neurology	390.6	390.6	200000	800000	2.7	9	5
Q6UX15	Neurology	3.1	6.1	6250	50000	3.0	9	6
Q53H47	Neurology	390.6	1562.5	400000	800000	2.4	8	4
P49789	Neurology	6.1	12.2	1563	6250	2.1	10	14
P05231	Neurology	0.8	1.5	1563	12500	3.0	10	8
P18031	Neurology						8	12
P29218	Neurology	195.3	781.3	200000	800000	2.4	9	9
P14868	Neurology	48.8	97.7	100000	400000	3.0	10	13
O95727	Neurology	12.2	48.8	12500	200000	2.4	9	8
P01138	Neurology	48.8	97.7	12500	25000	2.1	7	4
Q9BZM5	Neurology	24.4	48.8	100000	200000	3.3	9	8
Q8TCT1	Neurology	3.1	6.1	781	6250	2.1	8	6
Q2MKA7	Neurology	3.1	3.1	1563	12500	2.7	8	7
Q9NQ88	Neurology	24.4	195.3	100000	400000	2.7	7	24
Q9Y680	Neurology	97.7	195.3	100000	800000	2.7	7	10
Q86WV1	Neurology	48.8	195.3	50000	200000	2.4	8	8
O14944	Neurology	6.1	6.1	1563	6250	2.4	6	6
Q13451	Neurology	97.7	195.3	25000	50000	2.1	9	6
Q9Y4K4	Neurology	12.2	48.8	6250	50000	2.1	6	10
P16444	Neurology						8	5
O76070	Neurology	24.4	48.8	50000	800000	3.0	9	8
Q15814	Neurology	781.3	781.3	400000	800000	2.7	8	8

P08962	Neurology	24.4	48.8	12500	50000	2.4	9	6
O15232	Neurology	12.2	12.2	25000	200000	3.3	7	6
P48740	Neurology	390.6	781.3	100000	400000	2.1	10	6
P38484	Neurology	24.4	48.8	12500	100000	2.4	9	4
Q6ISS4	Neurology	0.8	1.5	781	6250	2.7	10	6
Q6P1N0	Neurology	97.7	195.3	100000	800000	2.7	9	6
Q07011	Neurology	0.8	1.5	3125	6250	3.3	8	6
Q8WWN9	Neurology	12.2	97.7	25000	50000	2.4	11	6
O15197	Neurology	12.2	48.8	25000	50000	2.7	9	6
Q9NPH6	Neurology	0.8	3.1	3125	12500	3.0	9	6
P10145	Neurology	0.2	0.4	1563	6250	3.6	7	5
Q9P0K1	Neurology	97.7	195.3	50000	200000	2.4	9	6
Q9H477	Neurology	6.1	6.1	12500	50000	3.3	7	11
Q8N474	Neurology	24.4	48.8	50000	200000	3.0	18	20
P40222	Neurology	3.1	6.1	1563	12500	2.4	9	8
P02771	Neurology	12.2	24.4	25000	100000	3.0	9	4
Q02790	Neurology	48.8	195.3	25000	50000	2.1	8	7
Q96GW7	Neurology	781.3	1562.5	200000	800000	2.1	7	5
Q9NZQ7	Neurology	3.1	3.1	3125	50000	3.0	9	6
P01258	Neurology	24.4	48.8	25000	50000	2.7	8	4
Q14108	Neurology	3.1	6.1	6250	50000	3.0	8	6
P56279	Neurology	97.7	195.3	50000	200000	2.4	10	6
Q9UL46	Neurology	48.8	195.3	25000	200000	2.1	7	7
Q9BS40	Neurology	390.6	390.6	200000	800000	2.7	10	12
P23588	Neurology	390.6	390.6	100000	200000	2.4	8	20
P08134	Neurology	24.4	48.8	50000	200000	3.0	8	8
Q8NFP4	Neurology	48.8	97.7	25000	200000	2.4	8	4
P22079	Neurology	3.1	12.2	25000	200000	3.3	7	8
O43557	Neurology	48.8	195.3	50000	200000	2.4	7	6
Q6XZF7	Neurology	12.2	24.4	50000	200000	3.3	9	6
P37023	Neurology	3.1	6.1	3125	12500	2.7	9	5
O95407	Neurology	97.7	195.3	50000	200000	2.4	10	8
Q06830	Neurology	97.7	195.3	25000	200000	2.1	10	12

Q8WV92	Neurology	3.1	6.1	25000	50000	3.6	9	8
Q92752	Neurology	97.7	195.3	50000	200000	2.4	9	7
Q99426	Neurology	24.4	97.7	100000	400000	3.0	7	10
P12644	Neurology	6.1	48.8	12500	50000	2.4	18	49
Q9BYC5	Neurology						7	6
O43927	Neurology	3.1	6.1	1563	6250	2.4	6	10
P15151	Neurology	97.7	195.3	200000	800000	3.0	9	8
Q08345	Neurology	12.2	12.2	50000	400000	3.6	7	5
P06733	Neurology						7	11
Q96GP6	Neurology	48.8	97.7	200000	400000	3.3	6	7
P14384	Neurology	12.2	24.4	25000	200000	3.0	5	5
O14594	Neurology	3.1	6.1	6250	50000	3.0	6	8
Q96B86	Neurology	24.4	48.8	100000	400000	3.3	7	7
Q6P4E1	Neurology	24.4	48.8	50000	100000	3.0	7	5
O14625	Neurology	1.5	6.1	6250	12500	3.0	7	9
P35237	Neurology	24.4	97.7	50000	400000	2.7	7	7
P52823	Neurology						7	7
Q08708	Neurology	3.1	6.1	6250	25000	3.0	7	9
P01178	Neurology	6.1	24.4	25000	200000	3.0	5	6
P17405	Neurology	97.7	195.3	400000	800000	3.3	6	9
P50225	Neurology	97.7	195.3	100000	400000	2.7	5	14
P15311	Neurology	390.6	781.3	100000	400000	2.1	6	4
P13611	Neurology	1.5	3.1	6250	25000	3.3	7	6
Q8NBJ7	Neurology	24.4	48.8	6250	200000	2.1	6	5
Q8NBS9	Neurology	3125.0	6250.0	800000	3200000	2.1	10	9
P55291	Neurology	1562.5	3125.0	400000	800000	2.1	7	6
P22223	Neurology	97.7	195.3	100000	200000	2.7	9	5
O14737	Neurology	6.1	24.4	12500	100000	2.7	8	13
P57087	Neurology	0.4	1.5	6250	25000	3.6	6	5
P21757	Neurology	12.2	24.4	12500	100000	2.7	6	8
Q9NP79	Neurology	195.3	390.6	400000	800000	3.0	9	15
P05060	Neurology	390.6	781.3	400000	800000	2.7	7	7
P78325	Neurology	3.1	6.1	12500	25000	3.3	5	7

O94907	Neurology	12.2	24.4	25000	50000	3.0	7	8
P30533	Neurology	6.1	12.2	6250	12500	2.7	5	6
Q9P126	Neurology	12.2	24.4	12500	50000	2.7	11	12
Q10589	Neurology	6.1	12.2	25000	50000	3.3	7	4
P01236	Neurology	24.4	97.7	100000	400000	3.0	6	11
Q92854	Neurology	97.7	195.3	50000	400000	2.4	6	7
P28906	Neurology	97.7	195.3	200000	400000	3.0	7	12
Q14739	Neurology	390.6	781.3	400000	800000	2.7	6	7
P01732	Neurology						6	10
Q99731	Neurology						5	12
Q9H446	Neurology	24.4	48.8	100000	200000	3.3	7	11
Q9UNZ2	Neurology	48.8	97.7	100000	800000	3.0	8	8
P21217_Q1	Neurology	1.5	6.1	6250	50000	3.0	5	7
Q9BRF8	Neurology	24.4	48.8	50000	200000	3.0	5	13
P14207	Neurology	3.1	6.1	6250	25000	3.0	6	5
P22894	Neurology	97.7	390.6	200000	800000	2.7	5	5
Q10588	Neurology	48.8	97.7	50000	800000	2.7	6	7
P09104	Neurology	3125.0	6250.0	3200000	12800000	2.7	6	13
Q6ZMJ2	Neurology	48.8	97.7	25000	100000	2.4	6	6
Q16288	Neurology	12.2	24.4	25000	100000	3.0	6	5
Q6NW40	Neurology	48.8	195.3	100000	200000	2.7	6	5
Q01469	Neurology	24.4	97.7	100000	800000	3.0	14	34
O00214	Neurology	12.2	48.8	25000	100000	2.7	5	6
Q9HCK4	Neurology	12.2	24.4	25000	200000	3.0	6	6
P13473	Neurology	97.7	195.3	200000	800000	3.0	5	6
P12429	Neurology	195.3	390.6	100000	200000	2.4	7	8
Q96CD2	Neurology	12.2	24.4	25000	200000	3.0	5	6
P28908	Neurology	12.2	48.8	50000	200000	3.0	5	6
P48052	Neurology	97.7	195.3	400000	800000	3.3	6	6
P25774	Neurology	1.5	12.2	12500	50000	3.0	7	6
O94779	Neurology	24.4	48.8	25000	100000	2.7	5	6
O75509	Neurology	24.4	48.8	25000	100000	2.7	6	6
P04216	Neurology	0.4	0.8	6250	25000	3.9	6	6

P04233	Neurology	97.7	195.3	25000	100000	2.1	5	5
Q9NZD4	Neurology	6.1	6.1	6250	25000	3.0	7	5
Q14112	Neurology	1562.5	3125.0	800000	800000	2.4	10	10
P01215	Neurology	0.8	1.5	6250	50000	3.6	7	6
Q96RD9	Neurology	6.1	12.2	25000	800000	3.3	7	3
Q9NPH3	Neurology	6.1	12.2	12500	50000	3.0	7	3
P16871	Neurology	6.1	12.2	12500	200000	3.0	7	15
Q9P232	Neurology	6.1	24.4	12500	50000	2.7	6	4
Q8N6Q3	Neurology	0.8	1.5	6250	25000	3.6	8	4
P06734	Neurology	1.5	3.1	6250	25000	3.3	7	6
P00749	Neurology	0.4	0.8	6250	12500	3.9	5	3
Q9Y240	Neurology	12.2	24.4	25000	100000	3.0	8	6
Q8TCZ2	Neurology	0.2	0.8	3125	6250	3.6	6	7
P20774	Neurology	6.1	24.4	25000	50000	3.0	6	4
Q96FE7	Neurology	6.1	12.2	6250	25000	2.7	5	4
Q6UXH9	Neurology	24.4	48.8	25000	50000	2.7	7	5
Q969Z4	Neurology	3.1	12.2	6250	25000	2.7	8	4
Q9NQ38	Neurology	97.7	195.3	25000	50000	2.1	7	3
P20333	Neurology	3.1	12.2	12500	50000	3.0	7	4
P14174	Neurology	6.1	12.2	3125	100000	2.4	7	14
Q9UBW5	Neurology	3125.0	6250.0	400000	800000	1.8	8	12
P12081	Neurology	48.8	195.3	100000	200000	2.7	6	16
P04118	Neurology	24.4	48.8	50000	400000	3.0	9	15
P30740	Neurology	97.7	390.6	400000	800000	3.0	7	8
P23381	Neurology	48.8	97.7	200000	400000	3.3	7	17
Q86T13	Neurology	48.8	97.7	200000	800000	3.3	5	5
Q99972	Neurology						6	8
Q15155	Neurology	6.1	12.2	25000	200000	3.3	5	5
Q15818	Neurology	6.1	12.2	25000	200000	3.3	7	5
Q9UGT4	Neurology	3.1	6.1	12500	50000	3.3	7	6
P05164	Neurology	97.7	195.3	12500	25000	1.8	6	6
P55103	Neurology	24.4	24.4	12500	50000	2.7	8	7
P22692	Neurology	390.6	390.6	100000	400000	2.4	8	5

P11215	Neurology	97.7	195.3	200000	400000	3.0	8	6
P02749	Neurology						9	12
P50895	Neurology	24.4	48.8	6250	12500	2.1	6	6
P19440	Neurology	6.1	24.4	50000	400000	3.3	6	5
P31948	Neurology	24.4	97.7	25000	200000	2.4	9	7
O76076	Neurology						11	13
P30086	Neurology	48.8	97.7	50000	400000	2.7	9	10
Q96F46	Neurology	3.1	6.1	12500	25000	3.3	8	5
P08254	Neurology	6.1	12.2	6250	25000	2.7	6	4
P14778	Neurology	0.4	0.4	6250	12500	4.2	5	5
Q04900	Neurology	24.4	48.8	12500	25000	2.4	8	5
Q02487	Neurology	6.1	24.4	25000	200000	3.0	8	5
O60462	Neurology	24.4	48.8	25000	200000	2.7	10	7
O15389	Neurology	97.7	195.3	100000	200000	2.7	8	6
P05067	Neurology	195.3	390.6	200000	400000	2.7	5	7
Q6YHK3	Neurology	48.8	97.7	200000	800000	3.3	5	8
P04179	Neurology	390.6	1562.5	800000	800000	2.7	7	4
P00352	Neurology	48.8	97.7	100000	200000	3.0	6	6
O43278	Neurology	6.1	24.4	25000	400000	3.0	5	3
O76061	Neurology	1.5	3.1	6250	25000	3.3	6	5
Q14126	Neurology	195.3	390.6	25000	50000	1.8	7	5
Q9Y279	Neurology	3.1	6.1	6250	25000	3.0	6	5
P11717	Neurology	97.7	97.7	50000	200000	2.7	7	6
P15289	Neurology	24.4	97.7	25000	50000	2.4	5	6
Q99727	Neurology	3.1	6.1	3125	12500	2.7	7	5
Q02747	Neurology	1.5	3.1	6250	25000	3.3	5	6
O15117	Neurology	48.8	97.7	50000	200000	2.7	8	8
Q9HCN6	Neurology	3.1	3.1	6250	25000	3.3	7	6
Q5T2D2	Neurology	3.1	6.1	6250	25000	3.0	7	5
P22105	Neurology	97.7	390.6	50000	400000	2.1	6	3
P16284	Neurology	6.1	12.2	50000	100000	3.6	8	4
Q9Y624	Neurology	0.4	0.8	3125	6250	3.6	11	6
P30043	Neurology	97.7	390.6	200000	400000	2.7	8	6

P14209	Neurology	1.5	3.1	6250	25000	3.3	6	3
P01833	Neurology	48.8	97.7	50000	200000	2.7	6	4
Q8IWV2	Neurology	48.8	97.7	50000	400000	2.7	6	5
P22749	Neurology	3.1	6.1	12500	50000	3.3	8	9
P14780	Neurology	97.7	195.3	50000	400000	2.4	7	5
P04155	Neurology	3.1	6.1	6250	25000	3.0	7	5
P28799	Neurology	12.2	48.8	12500	25000	2.4	7	5
P00918	Neurology	97.7	195.3	200000	800000	3.0	8	6
Q99497	Neurology	6.1	12.2	3125	12500	2.4	7	9
P17538	Neurology	1.5	3.1	12500	25000	3.6	6	4
Q9UKK9	Neurology	48.8	97.7	25000	50000	2.4	7	10
Q08431	Neurology	48.8	97.7	200000	800000	3.3	8	5
Q9UKJ1	Neurology	0.8	1.5	12500	25000	3.9	5	4
Q16881	Neurology	1.5	6.1	6250	25000	3.0	8	4
Q14696	Neurology	1562.5	3125.0	800000	800000	2.4	8	13
P35442	Neurology	24.4	48.8	100000	800000	3.3	6	4
Q8TDQ0	Neurology	1.5	3.1	6250	25000	3.3	6	4
Q13093	Neurology	195.3	390.6	200000	800000	2.7	7	5
P08648	Neurology	24.4	48.8	50000	400000	3.0	10	9
P63313	Neurology	48.8	97.7	12500	25000	2.1	7	6
P20827	Neurology	97.7	195.3	12500	25000	1.8	8	7
P00995	Neurology	24.4	48.8	12500	25000	2.4	8	5
Q8N149	Neurology	24.4	48.8	12500	50000	2.4	6	4
P07108	Neurology	12.2	24.4	12500	25000	2.7	7	7
P19438	Neurology	1.5	6.1	12500	25000	3.3	7	3
P23280	Neurology	6.1	12.2	12500	25000	3.0	9	7
Q6UXG3	Neurology	24.4	48.8	6250	25000	2.1	7	6
O43505	Neurology	24.4	97.7	50000	400000	2.7	7	6
O15394	Neurology	24.4	48.8	25000	200000	2.7	7	5
Q5SXM8	Neurology II	3125.0	3125.0	200000	800000	1.8	19	26
Q96Q89	Neurology II	12.2	48.8	12500	100000	2.4	18	12
Q9NQW8	Neurology II	781.3	781.3	50000	200000	1.8	15	17
P26441	Neurology II	195.3	390.6	400000	800000	3.0		

Q96GG9	Neurology II	1562.5	3125.0	200000	800000	1.8	18	
P48169	Neurology II	3125.0	3125.0	100000	400000	1.5		
P49792	Neurology II	3125.0	3125.0	400000	800000	2.1		
Q03721	Neurology II	195.3	390.6	25000	100000	1.8	6	10
Q96L15	Neurology II	390.6	1562.5	100000	400000	1.8	13	
Q8N111	Neurology II	6.1	24.4	6250	25000	2.4	15	13
Q96P66	Neurology II	48.8	97.7	12500	50000	2.1	9	7
P35221	Neurology II	6250.0	6250.0	200000	800000	1.5	7	20
Q13936	Neurology II	781.3	781.3	100000	800000	2.1	11	36
O95372	Neurology II						8	32
P04234	Neurology II	390.6	390.6	50000	400000	2.1	4	
P25440	Neurology II	1562.5	3125.0	200000	400000	1.8	7	6
O95049	Neurology II	1562.5	3125.0	400000	800000	2.1	12	20
P08908	Neurology II	1562.5	1562.5	100000	800000	1.8	11	20
Q30KQ4	Neurology II	195.3	195.3	12500	100000	1.8	10	4
Q13509	Neurology II	1562.5	1562.5	200000	800000	2.1	7	
Q8IVU1	Neurology II	1562.5	1562.5	100000	800000	1.8	13	
Q6P5Q4	Neurology II	781.3	1562.5	50000	400000	1.5	19	35
Q9NZA1	Neurology II	12.2	24.4	6250	25000	2.4	8	6
P0C7M8	Neurology II	195.3	195.3	50000	200000	2.4	13	
P63172	Neurology II	390.6	781.3	50000	100000	1.8	14	19
Q02641	Neurology II	781.3	781.3	50000	400000	1.8	7	
Q14894	Neurology II						13	17
O76039	Neurology II	1562.5	1562.5	100000	800000	1.8	7	33
Q7RTW8	Neurology II	97.7	195.3	50000	400000	2.4	10	20
Q5U5Z8	Neurology II	390.6	781.3	50000	800000	1.8	21	22
P07954	Neurology II						10	27
Q8N8R5	Neurology II	1562.5	3125.0	400000	800000	2.1	14	15
O15350	Neurology II	1562.5	1562.5	800000	800000	2.7	4	7
Q9ULW2	Neurology II	200000.0	200000.0	6400000	12800000	1.5		
Q9Y3E2	Neurology II	1562.5	3125.0	400000	800000	2.1	19	11
Q9BZ29	Neurology II	195.3	390.6	100000	400000	2.4	12	34
P47874	Neurology II	6.1	24.4	3125	25000	2.1	5	10

Q8IWB1	Neurology II	195.3	195.3	12500	800000	1.8	13	15
P23435	Neurology II	6250.0	6250.0	400000	1600000	1.8	14	
Q99700	Neurology II	781.3	781.3	50000	400000	1.8	12	28
Q8WTQ1	Neurology II	97.7	390.6	25000	100000	1.8	10	21
Q9P2M7	Neurology II	781.3	1562.5	100000	200000	1.8	9	8
Q14129	Neurology II	390.6	781.3	50000	100000	1.8	13	13
Q13002	Neurology II	12.2	24.4	50000	100000	3.3	12	17
Q9H492	Neurology II	3125.0	3125.0	800000	800000	2.4	10	
Q6QNY1	Neurology II	195.3	390.6	25000	100000	1.8	24	5
P26378	Neurology II	24.4	195.3	12500	50000	1.8	15	24
Q86T26	Neurology II	390.6	781.3	50000	800000	1.8	4	
Q99653	Neurology II	12500.0	12500.0	400000	800000	1.5	13	22
Q6QEF8	Neurology II	3125.0	12500.0	800000	800000	1.8	14	21
Q12774	Neurology II	390.6	390.6	25000	50000	1.8	16	25
Q8WVC0	Neurology II	781.3	781.3	100000	800000	2.1	8	13
Q9NXH3	Neurology II	781.3	781.3	50000	200000	1.8	10	19
Q13084	Neurology II	1562.5	1562.5	800000	800000	2.7	9	6
P30419	Neurology II						8	32
Q8N967	Neurology II	24.4	48.8	12500	50000	2.4	9	9
Q8IZJ0	Neurology II	3125.0	3125.0	400000	800000	2.1	6	10
Q15042	Neurology II	1562.5	1562.5	50000	400000	1.5	8	11
Q96PV0	Neurology II	48.8	97.7	12500	100000	2.1	5	13
P05549	Neurology II	3125.0	6250.0	200000	800000	1.5	13	
Q0VDD7	Neurology II	25000.0	25000.0	1600000	12800000	1.8	11	27
P28222	Neurology II						8	4
O15382	Neurology II	195.3	390.6	50000	400000	2.1		
Q687X5	Neurology II	48.8	48.8	12500	100000	2.4	11	17
Q5T871	Neurology II						4	
Q9UL42	Neurology II	12500.0	12500.0	800000	800000	1.8	7	22
O95196	Neurology II	390.6	390.6	50000	800000	2.1	16	23
Q9C0A0	Neurology II	390.6	390.6	25000	100000	1.8	7	17
O15013	Neurology II	390.6	1562.5	100000	200000	1.8	6	22
P12319	Neurology II	390.6	781.3	50000	200000	1.8	12	11

P54284	Neurology II	781.3	1562.5	100000	200000	1.8	11	35
Q96I25	Neurology II						13	25
Q01432	Neurology II						4	21
Q9Y6X8	Neurology II	390.6	390.6	50000	100000	2.1	6	8
Q8WW22	Neurology II						8	16
P04439	Neurology II						9	9
Q9NWU2	Neurology II	781.3	781.3	25000	100000	1.5	6	
Q5SW96	Neurology II	12500.0	12500.0	800000	1600000	1.8		
O15240	Neurology II						9	10
Q92859	Neurology II	97.7	97.7	25000	50000	2.4	8	12
Q14627	Neurology II	1562.5	1562.5	800000	800000	2.7	10	
Q9Y3B9	Neurology II	781.3	781.3	100000	800000	2.1		
Q96A32	Neurology II	3125.0	3125.0	100000	800000	1.5	14	25
Q9NPB3	Neurology II	1562.5	3125.0	200000	800000	1.8	16	25
Q9ULU8	Neurology II	195.3	390.6	100000	200000	2.4	10	13
Q6GQQ9	Neurology II	390.6	781.3	200000	800000	2.4	8	19
Q96HC4	Neurology II	390.6	781.3	100000	400000	2.1	7	37
Q9BV20	Neurology II						9	36
Q14184	Neurology II	195.3	390.6	100000	200000	2.4	8	15
P43007	Neurology II	12500.0	25000.0	1600000	6400000	1.8	9	20
Q68J44	Neurology II	390.6	390.6	50000	200000	2.1	16	22
P14649	Neurology II	1562.5	1562.5	100000	400000	1.8	12	16
Q96G03	Neurology II	390.6	781.3	200000	800000	2.4	11	28
Q9NY46	Neurology II	781.3	781.3	50000	800000	1.8		
O75718	Neurology II						4	
B6A8C7	Neurology II	195.3	390.6	50000	800000	2.1		
Q9Y4C0	Neurology II	390.6	390.6	25000	800000	1.8	6	31
Q7L311	Neurology II	781.3	1562.5	100000	800000	1.8		
Q15735	Neurology II	195.3	195.3	50000	100000	2.4	16	29
P48431	Neurology II	781.3	1562.5	100000	400000	1.8	2	
Q6ZVN8	Neurology II	781.3	1562.5	100000	200000	1.8	12	10
O43665	Neurology II	195.3	195.3	25000	100000	2.1	8	24
Q6Y7W6	Neurology II	390.6	390.6	50000	200000	2.1	9	16

Q8WXW3-4	Neurology II	195.3	195.3	50000	200000	2.4	11	31
Q9H4X1	Neurology II	1562.5	3125.0	200000	800000	1.8	8	31
Q16625	Neurology II	195.3	195.3	25000	100000	2.1	9	12
P78318	Neurology II	1562.5	1562.5	100000	400000	1.8	11	21
Q6ZVM7	Neurology II						12	37
Q9H6S1	Neurology II	781.3	1562.5	400000	800000	2.4	10	25
P52179	Neurology II	195.3	781.3	50000	200000	1.8	12	
Q99250	Neurology II	195.3	390.6	25000	100000	1.8	9	23
O75631	Neurology II	781.3	781.3	50000	200000	1.8		
Q96J84	Neurology II	1562.5	1562.5	200000	800000	2.1	9	14
Q9UKV0	Neurology II						8	22
Q19T08	Neurology II						0.1	
Q9ULH4	Neurology II						21	
O95295	Neurology II	781.3	781.3	100000	400000	2.1	11	23
A8MVW0	Neurology II	781.3	1562.5	100000	400000	1.8	9	28
Q9BZE9	Neurology II	390.6	781.3	200000	800000	2.4	14	22
P61764	Neurology II	1562.5	3125.0	400000	800000	2.1		
P68400	Neurology II						7	22
Q13224	Neurology II	1562.5	6250.0	400000	800000	1.8	9	12
O75312	Neurology II	781.3	781.3	50000	800000	1.8	13	23
P60880	Neurology II	97.7	390.6	50000	400000	2.1	14	23
A6NGG8	Neurology II	390.6	781.3	50000	100000	1.8	13	8
P05787	Neurology II	24.4	195.3	400000	800000	3.3	13	28
Q99856	Neurology II						12	
Q96PH6	Neurology II	3125.0	6250.0	200000	800000	1.5	3	1
P52788	Neurology II	3125.0	3125.0	200000	400000	1.8	9	21
Q9Y6U3	Neurology II	48.8	48.8	25000	100000	2.7	12	16
Q9Y285	Neurology II	97.7	781.3	100000	200000	2.1	10	19
Q9HCM4	Neurology II	781.3	1562.5	100000	400000	1.8	19	31
P26715	Neurology II	195.3	390.6	50000	100000	2.1	2	
Q6P9F5	Neurology II							
Q8WXG9	Neurology II	781.3	781.3	100000	400000	2.1	19	34
O15020	Neurology II	195.3	390.6	25000	100000	1.8	7	

P55283	Neurology II	390.6	781.3	50000	200000	1.8	9	2
P10523	Neurology II	3125.0	3125.0	200000	400000	1.8		
O14503	Neurology II	195.3	390.6	50000	400000	2.1	11	26
O43396	Neurology II						11	32
Q8IY33	Neurology II	1562.5	1562.5	50000	800000	1.5	16	15
Q2M3V2	Neurology II	781.3	1562.5	200000	800000	2.1	14	38
P48775	Neurology II	12.2	48.8	12500	50000	2.4	12	16
P61366	Neurology II	3125.0	3125.0	100000	200000	1.5	11	21
Q10571	Neurology II	781.3	781.3	50000	800000	1.8	11	24
Q8N2Q7	Neurology II	1562.5	1562.5	100000	800000	1.8	13	16
Q12809	Neurology II	1562.5	1562.5	50000	400000	1.5	6	7
O15212	Neurology II	3125.0	6250.0	800000	800000	2.1	18	18
P61266	Neurology II	390.6	1562.5	200000	800000	2.1	13	10
Q86UP6	Neurology II	6.1	24.4	6250	50000	2.4	8	33
P0DKB5	Neurology II	25000.0	25000.0	1600000	6400000	1.8	16	34
P11229	Neurology II	1562.5	3125.0	200000	800000	1.8	12	5
Q5VT99	Neurology II	1562.5	3125.0	400000	800000	2.1	9	10
Q16650	Neurology II	1562.5	1562.5	200000	800000	2.1	6	
Q6ZUT3	Neurology II	1562.5	1562.5	50000	800000	1.5	7	3
Q6UWJ8	Neurology II	390.6	781.3	50000	200000	1.8		
P63211	Neurology II	390.6	390.6	25000	100000	1.8	11	14
P20823	Neurology II	6250.0	6250.0	400000	800000	1.8	9	8
Q16401	Neurology II	390.6	390.6	100000	800000	2.4	11	13
O75121	Neurology II						6	17
O15234	Neurology II						10	21
Q86UW9	Neurology II	1562.5	3125.0	200000	800000	1.8	13	9
Q86WK6	Neurology II	781.3	781.3	25000	800000	1.5	11	21
O15083	Neurology II	97.7	195.3	100000	200000	2.7	16	23
Q53GD3	Neurology II	195.3	390.6	100000	200000	2.4	5	
Q8NFZ4	Neurology II	3125.0	3125.0	200000	800000	1.8	10	
P54105	Neurology II	1562.5	1562.5	100000	800000	1.8	10	23
Q93015	Neurology II	781.3	3125.0	200000	400000	1.8	11	30
Q9UHL0	Neurology II							

Q8N6Q1	Neurology II	390.6	1562.5	100000	200000	1.8	14	19
Q16520	Neurology II	48.8	97.7	25000	50000	2.4	11	9
Q96KJ4	Neurology II	781.3	1562.5	100000	800000	1.8	11	16
Q9UGI9	Neurology II	12500.0	12500.0	400000	1600000	1.5		
Q53EL9	Neurology II	781.3	781.3	50000	200000	1.8	15	21
P04062	Neurology II	50000.0	50000.0	1600000	6400000	1.5		
Q7L0J3	Neurology II	195.3	390.6	100000	400000	2.4	19	29
P01270	Neurology II							
Q8IY31	Neurology II	781.3	781.3	25000	100000	1.5	12	13
P10301	Neurology II	390.6	390.6	100000	200000	2.4	16	30
P98164	Neurology II	1562.5	1562.5	100000	800000	1.8	23	34
Q92834	Neurology II	781.3	781.3	50000	200000	1.8	15	16
O14967	Neurology II	6.1	48.8	25000	50000	2.7	16	22
Q9H461	Neurology II						22	13
O43474	Neurology II	781.3	1562.5	200000	800000	2.1	12	14
Q9NY72	Neurology II	6250.0	6250.0	800000	800000	2.1		
P43220	Neurology II							
P41732	Neurology II						11	
Q17R60	Neurology II	781.3	1562.5	100000	400000	1.8	10	10
Q9UKW4	Neurology II	97.7	195.3	25000	100000	2.1	7	19
Q9UJC5	Neurology II	6250.0	12500.0	800000	3200000	1.8	13	28
Q96BJ3	Neurology II							
O60218	Neurology II	6250.0	6250.0	200000	800000	1.5	16	
P52799	Neurology II	390.6	781.3	100000	800000	2.1	22	17
P43146	Neurology II						11	11
P49069	Neurology II	781.3	1562.5	100000	200000	1.8	12	26
P09683	Neurology II						9	26
Q7Z3D4	Neurology II	97.7	195.3	100000	400000	2.7	14	39
P24386	Neurology II	195.3	390.6	25000	100000	1.8		
Q96QH8	Neurology II	12.2	24.4	6250	25000	2.4	9	11
Q9UM54	Neurology II						8	23
P08651	Neurology II	3125.0	3125.0	800000	800000	2.4	10	19
Q9HCY8	Neurology II	195.3	390.6	50000	400000	2.1	10	17

O60662	Neurology II	390.6	390.6	200000	400000	2.7	14	20
Q96JB5	Neurology II						12	16
A6NFN3	Neurology II	24.4	48.8	6250	100000	2.1	9	10
Q9P2M1	Neurology II						10	11
Q9HBL6	Neurology II	3125.0	3125.0	100000	200000	1.5	9	20
P10746	Neurology II	195.3	390.6	25000	800000	1.8		
Q2UY09	Neurology II	781.3	781.3	100000	400000	2.1	13	20
Q9NPD7	Neurology II						6	
Q9BY14	Neurology II	12.2	24.4	6250	25000	2.4	12	19
Q9BZJ3	Neurology II	195.3	390.6	50000	200000	2.1	12	14
P48436	Neurology II	781.3	1562.5	100000	400000	1.8	16	
Q9NUG6	Neurology II	3125.0	3125.0	100000	800000	1.5	14	19
P09455	Neurology II	390.6	781.3	50000	800000	1.8	12	20
Q0P6D2	Neurology II	3125.0	3125.0	200000	800000	1.8	10	22
Q8WWM7	Neurology II	3125.0	3125.0	400000	800000	2.1	7	11
Q07617	Neurology II						11	15
Q9BX66	Neurology II	1562.5	1562.5	50000	200000	1.5	6	17
Q15398	Neurology II	195.3	195.3	50000	400000	2.4	6	23
P35556	Neurology II							
Q8IWP9	Neurology II							
Q9UNY4	Neurology II	390.6	390.6	25000	100000	1.8		
Q9UH03	Neurology II	781.3	1562.5	800000	800000	2.7	7	18
P53367	Neurology II	390.6	1562.5	400000	800000	2.4	13	29
P60763	Neurology II						9	11
P63010	Neurology II						12	20
O14578	Neurology II	48.8	48.8	25000	100000	2.7	15	30
O60939	Neurology II	781.3	1562.5	100000	800000	1.8		
P20916	Neurology II						19	22
Q9BX10	Neurology II	781.3	781.3	50000	200000	1.8	12	24
Q86TM3	Neurology II	390.6	781.3	100000	800000	2.1	12	
Q15102	Neurology II	6250.0	6250.0	800000	800000	2.1	10	
P54277	Neurology II	48.8	97.7	12500	50000	2.1	21	24
P50897	Neurology II	3125.0	3125.0	200000	800000	1.8	9	26

Q8TC05	Neurology II	1562.5	1562.5	100000	200000	1.8	11	19
O95202	Neurology II	781.3	781.3	50000	200000	1.8	11	22
Q9UQ16	Neurology II	390.6	390.6	100000	800000	2.4	9	22
Q9H6Q3	Neurology II	3125.0	6250.0	800000	800000	2.1	10	35
Q15714	Neurology II	390.6	390.6	25000	100000	1.8	13	19
Q8IVM0	Neurology II	781.3	1562.5	400000	800000	2.4	10	13
Q86YD3	Neurology II	97.7	97.7	25000	100000	2.4	9	11
P29536	Neurology II	24.4	48.8	12500	50000	2.4	8	23
O95954	Neurology II	781.3	3125.0	100000	800000	1.5	5	19
Q5T848	Neurology II	781.3	781.3	50000	200000	1.8	9	9
B2RUY7	Neurology II	48.8	97.7	12500	50000	2.1	9	16
Q14151	Neurology II	781.3	1562.5	100000	400000	1.8	9	10
Q92888	Neurology II	12500.0	12500.0	6400000	12800000	2.7	6	23
Q96A00	Neurology II						11	32
Q15366	Neurology II	25000.0	25000.0	800000	3200000	1.5	7	23
O60869	Neurology II						13	22
O75167	Neurology II	781.3	781.3	100000	800000	2.1		
O95467	Neurology II						7	25
P22102	Neurology II	781.3	1562.5	50000	200000	1.5	5	23
Q07021	Neurology II	48.8	195.3	50000	100000	2.4	8	12
Q08378	Neurology II	781.3	781.3	400000	800000	2.7	10	17
O75792	Neurology II	1562.5	3125.0	400000	800000	2.1	9	21
O43432	Neurology II	1562.5	6250.0	400000	800000	1.8	11	21
Q9BUJ2	Neurology II	97.7	195.3	25000	50000	2.1	7	15
Q96PE7	Neurology II	6250.0	6250.0	400000	800000	1.8	11	12
Q6UWW0	Neurology II	97.7	390.6	100000	400000	2.4	8	12
P51687	Neurology II	97.7	390.6	50000	100000	2.1	9	18
Q01826	Neurology II	195.3	390.6	100000	400000	2.4	7	7
P04808	Neurology II	1562.5	6250.0	400000	800000	1.8	5	26
Q8WWV3	Neurology II	781.3	781.3	200000	800000	2.4	8	35
Q9H6H4	Neurology II	195.3	390.6	100000	800000	2.4	6	9
O60469	Neurology II	195.3	195.3	50000	200000	2.4	9	7
O75592	Neurology II	390.6	390.6	100000	800000	2.4	9	20

Q9H777	Neurology II	390.6	390.6	50000	400000	2.1	9	25
Q14197	Neurology II	781.3	1562.5	50000	800000	1.5	12	22
Q5JSP0	Neurology II	97.7	195.3	25000	100000	2.1	15	30
O43776	Neurology II						11	10
P31751	Neurology II	6250.0	12500.0	800000	800000	1.8		
P29377	Neurology II	97.7	195.3	25000	200000	2.1	10	14
P14902	Neurology II	195.3	390.6	200000	400000	2.7	14	12
Q99460	Neurology II						15	26
Q9BXI9	Neurology II	1562.5	1562.5	200000	800000	2.1	14	30
Q5T5Y3	Neurology II	97.7	195.3	25000	100000	2.1	11	29
Q6PKH6	Neurology II	781.3	781.3	200000	800000	2.4	6	11
Q13277	Neurology II	1562.5	1562.5	200000	800000	2.1	7	5
P21579	Neurology II	195.3	195.3	50000	800000	2.4	7	10
Q53GL0	Neurology II	1562.5	6250.0	400000	800000	1.8	8	16
O60890	Neurology II	3125.0	3125.0	200000	800000	1.8	9	21
O15269	Neurology II	1562.5	3125.0	800000	800000	2.4	7	12
Q9H1P3	Neurology II	781.3	1562.5	200000	800000	2.1	5	7
Q96RU2	Neurology II	195.3	390.6	100000	400000	2.4	8	8
P01350	Neurology II						6	30
P78352	Neurology II	195.3	390.6	25000	100000	1.8	8	11
P26718	Neurology II	48.8	97.7	25000	50000	2.4	10	13
O77932	Neurology II	97.7	97.7	6250	25000	1.8	6	16
Q92599	Neurology II	195.3	390.6	200000	800000	2.7	5	9
P13995	Neurology II	195.3	195.3	12500	50000	1.8	6	15
Q8N163	Neurology II	781.3	1562.5	400000	800000	2.4	9	16
Q8IWY9	Neurology II	50000.0	50000.0	3200000	12800000	1.8		
Q86VP1	Neurology II	24.4	24.4	12500	25000	2.7	6	11
Q9H251	Neurology II	1562.5	3125.0	100000	800000	1.5	8	13
Q14149	Neurology II	1562.5	3125.0	800000	800000	2.4	5	5
P43320	Neurology II	97.7	195.3	100000	400000	2.7	6	14
P51649	Neurology II	3125.0	6250.0	200000	400000	1.5	11	15
Q9NWM8	Neurology II	781.3	781.3	25000	100000	1.5	9	33
Q14160	Neurology II	390.6	781.3	50000	400000	1.8	10	24

Q9BUP0	Neurology II	195.3	390.6	50000	200000	2.1	9	15
O94830	Neurology II	195.3	390.6	25000	100000	1.8	7	16
Q8IWQ3	Neurology II	3125.0	3125.0	100000	400000	1.5	8	21
Q9NPG4	Neurology II						6	8
Q6UXV0	Neurology II	12.2	24.4	12500	50000	2.7	7	17
Q4ZHG4	Neurology II						8	17
Q13938	Neurology II	48.8	48.8	12500	200000	2.4	7	9
P47813	Neurology II						12	25
Q9UKY0	Neurology II	48.8	48.8	12500	25000	2.4	3	13
P54315	Neurology II						9	22
Q15256	Neurology II	48.8	48.8	25000	100000	2.7	6	10
O14530	Neurology II	6250.0	6250.0	200000	800000	1.5	5	17
Q15025	Neurology II	97.7	195.3	25000	200000	2.1	4	12
P13861	Neurology II	781.3	1562.5	400000	800000	2.4	4	13
P10092	Neurology II	195.3	195.3	200000	800000	3.0	8	10
Q9ULA0	Neurology II	390.6	390.6	50000	400000	2.1	3	19
P59780	Neurology II	25000.0	25000.0	3200000	12800000	2.1	7	11
Q8WYQ3	Neurology II	195.3	195.3	6250	25000	1.5	10	19
Q9NPE2	Neurology II	781.3	781.3	200000	400000	2.4	6	7
Q6UX71	Neurology II	781.3	781.3	50000	400000	1.8	10	19
Q6XQN6	Neurology II						6	9
P46937	Neurology II	781.3	1562.5	50000	100000	1.5	6	9
Q9BXI3	Neurology II	97.7	97.7	6250	25000	1.8	6	11
Q9P2X3	Neurology II	390.6	781.3	100000	400000	2.1	3	11
Q99704	Neurology II	6250.0	6250.0	200000	800000	1.5		
Q6P1J6	Neurology II	97.7	97.7	12500	100000	2.1	9	21
O60235	Neurology II	12.2	24.4	6250	25000	2.4	8	8
Q03252	Neurology II	1562.5	1562.5	800000	800000	2.7	6	13
Q8TAT2	Neurology II	390.6	390.6	12500	50000	1.5	7	8
Q5F1R6	Neurology II	390.6	390.6	25000	100000	1.8	11	17
Q03014	Neurology II	6.1	6.1	6250	25000	3.0	7	26
Q6QNY0	Neurology II	781.3	1562.5	50000	200000	1.5	7	17
Q9BPX1	Neurology II	390.6	781.3	200000	800000	2.4	6	22

P32455	Neurology II	3125.0	3125.0	3200000	6400000	3.0	14	25
Q9Y3C0	Neurology II	195.3	390.6	25000	200000	1.8	5	17
P62760	Neurology II	195.3	390.6	25000	100000	1.8	7	17
Q2L4Q9	Neurology II	3.1	6.1	12500	25000	3.3	7	10
P55210	Neurology II						7	19
P51460	Neurology II							
Q5VV43	Neurology II	195.3	195.3	25000	100000	2.1	7	11
A0FGR8	Neurology II	6250.0	12500.0	800000	800000	1.8	9	18
P11137	Neurology II	25000.0	50000.0	1600000	6400000	1.5	5	10
Q9BRJ6	Neurology II						5	7
Q7Z4V5	Neurology II						10	14
P08579	Neurology II	390.6	781.3	50000	400000	1.8	9	13
Q9BQT9	Neurology II	3125.0	6250.0	200000	800000	1.5	6	15
Q9UBC9	Neurology II						12	18
P0CG30	Neurology II	48.8	48.8	12500	100000	2.4	5	39
Q8IXS6	Neurology II	390.6	781.3	50000	400000	1.8	5	9
O75146	Neurology II						7	10
Q9NRY6	Neurology II	1562.5	1562.5	200000	800000	2.1	7	16
Q96CN9	Neurology II	97.7	195.3	50000	200000	2.4	4	36
Q9NXV2	Neurology II	97.7	195.3	50000	100000	2.4	8	11
P07320	Neurology II	3.1	24.4	3125	25000	2.1	5	15
P54252	Neurology II	1562.5	3125.0	100000	800000	1.5	6	13
Q8IXM2	Neurology II	195.3	390.6	50000	200000	2.1	7	22
A8MVW5	Neurology II	390.6	390.6	50000	400000	2.1	7	26
O60220	Neurology II	390.6	781.3	50000	100000	1.8	10	27
Q8TF65	Neurology II	195.3	195.3	25000	100000	2.1	7	8
P53814	Neurology II	1562.5	1562.5	50000	400000	1.5	5	12
Q07817	Neurology II							
Q00722	Neurology II	390.6	781.3	50000	200000	1.8		
P40313	Neurology II	97.7	97.7	12500	25000	2.1	7	22
Q8N4C8	Neurology II							
P54819	Neurology II						11	37
Q9NR46	Neurology II						6	16

P09110	Oncology	1562.5	3125.0	400000	800000	2.1	14	20
O14713	Oncology						11	14
Q13145	Oncology						7	33
Q9NX58	Oncology						9	32
O95498	Oncology	781.3	781.3	200000	800000	2.4	6	10
Q07954	Oncology	12.2	24.4	12500	50000	2.7	9	11
Q9BTE6	Oncology	48.8	195.3	200000	400000	3.0	8	16
Q6UWW8	Oncology	195.3	390.6	200000	400000	2.7	9	53
P01229	Oncology	6250.0	12500.0	800000	1600000	1.8	10	16
P05937	Oncology	0.8	1.5	6250	25000	3.6	8	13
P08069	Oncology	97.7	195.3	100000	800000	2.7	6	12
P00519	Oncology	12.2	24.4	50000	200000	3.3	9	11
Q96I82	Oncology	97.7	195.3	100000	200000	2.7	7	11
Q9BS26	Oncology	97.7	195.3	100000	400000	2.7	7	10
Q14241	Oncology	3.1	6.1	12500	50000	3.3	7	10
Q9HAV7	Oncology	12.2	24.4	50000	200000	3.3	7	13
Q9BSL1	Oncology	48.8	195.3	25000	800000	2.1	10	11
Q9C0C4	Oncology	24.4	48.8	50000	800000	3.0	10	8
O00592	Oncology	97.7	195.3	12500	100000	1.8	8	8
Q9UK85	Oncology	48.8	97.7	50000	800000	2.7	8	11
Q7Z5R6	Oncology	195.3	390.6	100000	400000	2.4	10	10
P30041	Oncology						6	15
P82980	Oncology	97.7	195.3	12500	50000	1.8	8	11
P47992	Oncology						4	7
Q9NSA1	Oncology	12.2	24.4	25000	200000	3.0	4	5
Q9NTU7	Oncology	97.7	195.3	200000	800000	3.0	4	27
Q14213_Q8	Oncology	97.7	390.6	400000	800000	3.0	3	8
P13726	Oncology	0.8	1.5	6250	25000	3.6	7	4
P06756	Oncology	48.8	97.7	12500	100000	2.1	3	4
P61218	Oncology	12.2	24.4	6250	12500	2.4	10	11
Q96NA2	Oncology	24.4	48.8	25000	50000	2.7	5	4
P50579	Oncology	781.3	1562.5	200000	800000	2.1	4	8
Q9UBG3	Oncology	1.5	3.1	6250	12500	3.3	3	4

Q14790	Oncology	0.8	1.5	6250	12500	3.6	6	13
P35637	Oncology	100000.0	100000.0	6400000	12800000	1.8	8	16
Q13490	Oncology	24.4	195.3	50000	200000	2.4	11	24
Q9UQB8	Oncology						9	11
Q6EIG7	Oncology	1.5	3.1	6250	12500	3.3	10	18
P80075	Oncology	0.8	1.5	1563	6250	3.0	4	5
O00292	Oncology						4	10
Q9BSG5	Oncology	97.7	195.3	50000	200000	2.4	9	14
Q99075	Oncology	0.8	1.5	3125	12500	3.3	4	4
Q9Y5W5	Oncology	48.8	48.8	25000	400000	2.7	4	6
P42658	Oncology	97.7	195.3	200000	800000	3.0	9	13
Q99717	Oncology	195.3	390.6	25000	200000	1.8	8	12
O43699	Oncology	12.2	24.4	25000	200000	3.0	3	5
Q86SJ6	Oncology	12.2	24.4	6250	25000	2.4	6	11
P35318	Oncology	781.3	1562.5	400000	800000	2.4	4	8
P35813	Oncology	390.6	781.3	50000	200000	1.8	8	14
Q7L5Y9	Oncology						9	42
P01375	Oncology	6.1	12.2	12500	100000	3.0	7	20
Q9Y265	Oncology	781.3	3125.0	400000	800000	2.1	11	15
P42331	Oncology	195.3	390.6	100000	800000	2.4	10	16
P06850	Oncology	1562.5	3125.0	400000	800000	2.1	12	27
Q8IUK5	Oncology	48.8	97.7	25000	200000	2.4	12	14
Q9BSW2	Oncology	24.4	195.3	25000	200000	2.1	11	16
O95388	Oncology	12.2	24.4	6250	25000	2.4	3	4
Q2VWP7	Oncology	6.1	12.2	6250	100000	2.7	5	6
Q00796	Oncology						6	11
O95786	Oncology	1562.5	3125.0	800000	800000	2.4	8	13
Q9UHF1	Oncology						11	18
P14136	Oncology	97.7	195.3	400000	800000	3.3	14	23
P31994	Oncology						12	27
P55789	Oncology	781.3	1562.5	100000	800000	1.8	9	17
P55273	Oncology						8	23
Q9Y243	Oncology	48.8	97.7	50000	200000	2.7	9	24

P22307	Oncology	24.4	195.3	100000	200000	2.7	12	12
P43628	Oncology	24.4	195.3	25000	200000	2.1	9	18
P31350	Oncology	390.6	781.3	50000	400000	1.8	11	20
P39748	Oncology						11	25
O14964	Oncology	3125.0	6250.0	800000	800000	2.1	11	19
Q9NRA1	Oncology	195.3	781.3	100000	800000	2.1	10	16
Q05516	Oncology	24.4	48.8	50000	200000	3.0	9	18
P48643	Oncology						9	38
P46060	Oncology	6250.0	6250.0	1600000	12800000	2.4	10	11
O75569	Oncology	3125.0	6250.0	400000	800000	1.8	11	15
Q6UX82	Oncology	24.4	48.8	25000	200000	2.7	10	16
Q99683	Oncology	6250.0	6250.0	1600000	12800000	2.4	13	21
P01242	Oncology	390.6	781.3	100000	200000	2.1	14	19
Q08AG7	Oncology	48.8	195.3	50000	100000	2.4	9	27
Q96DU3	Oncology	390.6	781.3	50000	800000	1.8	13	13
P43629	Oncology	6.1	12.2	50000	100000	3.6	15	35
O43752	Oncology	390.6	781.3	100000	800000	2.1	11	13
O60828	Oncology	195.3	390.6	50000	200000	2.1	8	22
P35070	Oncology	3.1	6.1	3125	12500	2.7	10	20
Q8IWL2	Oncology	3125.0	3125.0	100000	400000	1.5	10	29
Q7Z7D3	Oncology	24.4	48.8	50000	200000	3.0	11	41
P34130	Oncology	6.1	12.2	6250	200000	2.7	7	26
Q9UKR0	Oncology	24.4	48.8	12500	100000	2.4	8	19
Q6NXT1	Oncology	97.7	195.3	100000	200000	2.7	9	15
P54727	Oncology	1562.5	3125.0	400000	800000	2.1	12	16
Q6BAA4	Oncology	97.7	195.3	50000	200000	2.4	11	17
Q92982	Oncology	97.7	195.3	100000	800000	2.7	11	20
Q8NBZ7	Oncology	1562.5	3125.0	800000	800000	2.4	8	22
P41586	Oncology	24.4	48.8	6250	800000	2.1	8	9
O75787	Oncology						12	14
Q15797	Oncology	48.8	195.3	400000	800000	3.3	14	24
Q96NB1	Oncology	24.4	48.8	100000	800000	3.3	10	23
Q07960	Oncology	24.4	97.7	12500	50000	2.1	8	15

P50749	Oncology	195.3	390.6	100000	800000	2.4	10	18
Q6PGN9	Oncology						9	16
P06731	Oncology						15	23
Q8IWL1	Oncology	195.3	781.3	100000	200000	2.1	12	15
O14662	Oncology						10	20
Q7Z6M1	Oncology	1562.5	3125.0	800000	6400000	2.4	10	11
Q9UQQ2	Oncology	781.3	1562.5	100000	800000	1.8	9	16
P25786	Oncology	6250.0	12500.0	400000	800000	1.5	15	35
Q9H4P4	Oncology	312.5	625.0	320000	640000	2.7	10	11
O75493	Oncology	1562.5	3125.0	400000	800000	2.1	11	18
Q9NS15	Oncology	97.7	195.3	25000	100000	2.1	11	27
A4D1B5	Oncology						10	18
P49788	Oncology	97.7	781.3	100000	800000	2.1	10	14
P21810	Oncology	24.4	48.8	25000	100000	2.7	14	21
Q7LG56	Oncology						12	28
Q9P0J1	Oncology						8	16
Q9Y5V3	Oncology	3.1	3.1	1563	12500	2.7	9	54
Q8N5S9	Oncology	97.7	195.3	25000	200000	2.1	11	21
Q7Z434	Oncology	390.6	781.3	100000	800000	2.1	10	13
P07332	Oncology	6250.0	12500.0	400000	800000	1.5	5	
O15116	Oncology	48.8	195.3	25000	100000	2.1	9	43
P43490	Oncology	3125.0	3125.0	800000	1600000	2.4	13	16
O75380	Oncology	48.8	97.7	25000	100000	2.4	11	20
O60907	Oncology	24.4	48.8	12500	100000	2.4	11	19
Q01543	Oncology	1.5	3.1	1563	3125	2.7	7	37
Q9UKS7	Oncology	6.1	6.1	6250	25000	3.0	8	21
Q06787	Oncology	97.7	97.7	200000	800000	3.3	8	18
P04637	Oncology	781.3	1562.5	400000	800000	2.4	10	23
Q8WUX2	Oncology	97.7	195.3	50000	200000	2.4	7	19
Q9Y6A5	Oncology	24.4	195.3	25000	800000	2.1	8	25
P34949	Oncology	195.3	390.6	200000	800000	2.7	9	12
Q8WYN0	Oncology	1562.5	3125.0	800000	800000	2.4	8	15
Q96PQ0	Oncology	1562.5	1562.5	200000	800000	2.1	10	24

P15121	Oncology	1562.5	1562.5	400000	800000	2.4	11	18
P36888	Oncology	48.8	97.7	25000	100000	2.4	10	11
Q9Y662	Oncology	390.6	781.3	400000	800000	2.7	8	12
Q8TE58	Oncology	195.3	781.3	200000	800000	2.4	9	14
Q9BYE9	Oncology	3125.0	6250.0	800000	800000	2.1	7	12
P05231	Oncology	0.8	1.5	3125	12500	3.3	9	8
Q7L5N7	Oncology						8	15
P55008	Oncology						11	20
P40198	Oncology						16	31
Q9Y223	Oncology	781.3	1562.5	400000	800000	2.4	8	11
Q9Y5L3	Oncology	6.1	12.2	6250	25000	2.7	9	27
P05783	Oncology	6.1	12.2	12500	100000	3.0	7	11
Q8TD06	Oncology	50000.0	50000.0	6400000	12800000	2.1	11	13
Q9Y2Z0	Oncology						11	23
Q9P0V8	Oncology	6.1	48.8	6250	25000	2.1	10	18
P51580	Oncology	48.8	195.3	50000	200000	2.4	10	14
O43524	Oncology	1562.5	6250.0	200000	400000	1.5	13	13
O75695	Oncology	3125.0	6250.0	400000	800000	1.8	8	10
O00233	Oncology	48.8	195.3	100000	400000	2.7	9	9
Q9GZY6	Oncology	781.3	1562.5	400000	800000	2.4	7	16
Q5VIR6	Oncology	97.7	195.3	50000	200000	2.4	8	9
Q9UJ71	Oncology	48.8	48.8	50000	400000	3.0	10	13
Q86WD7	Oncology	6.1	12.2	50000	200000	3.6	7	16
Q15427	Oncology	97.7	195.3	25000	100000	2.1	9	14
P10606	Oncology	97.7	195.3	50000	100000	2.4	5	14
P51692	Oncology	24.4	48.8	200000	400000	3.6	14	20
P0CG37	Oncology	97.7	195.3	100000	200000	2.7	10	29
Q9H4A9	Oncology	97.7	390.6	50000	400000	2.1	10	11
P08473	Oncology	24.4	48.8	25000	100000	2.7	9	9
Q9NUY8	Oncology	48.8	97.7	12500	25000	2.1	10	26
P17948	Oncology	781.3	1562.5	200000	400000	2.1	8	9
P10747	Oncology	195.3	390.6	100000	800000	2.4	11	15
Q16772	Oncology	24.4	48.8	25000	100000	2.7	10	13

Q9BUE0	Oncology						10	14
O00186	Oncology						8	24
Q3B7J2	Oncology	100000.0	400000.0	12800000	12800000	1.5	8	16
Q6P2H3	Oncology	87.9	175.8	45000	180000	2.4	9	31
O00221	Oncology	24.4	48.8	25000	200000	2.7	10	15
Q9BQ51	Oncology	24.4	97.7	6250	400000	1.8	9	12
O94760	Oncology	97.7	390.6	100000	800000	2.4	11	11
Q9UHD8	Oncology						7	9
P30260	Oncology	6.1	12.2	50000	200000	3.6	10	29
Q9Y639	Oncology	390.6	781.3	50000	200000	1.8	12	15
O95831	Oncology	97.7	195.3	25000	800000	2.1	11	8
Q6UXD5	Oncology	48.8	195.3	100000	400000	2.7	7	7
O75054	Oncology	6.1	12.2	25000	200000	3.3	8	6
Q9Y570	Oncology	390.6	781.3	400000	800000	2.7	9	17
P07947	Oncology	24.4	48.8	12500	50000	2.4	11	16
P15848	Oncology	48.8	195.3	100000	800000	2.7	8	12
Q11201	Oncology	3125.0	6250.0	800000	800000	2.1	9	8
P55039	Oncology	3125.0	6250.0	800000	800000	2.1	7	12
Q8IX05	Oncology	195.3	390.6	25000	800000	1.8	10	9
Q12846	Oncology	195.3	390.6	100000	800000	2.4	9	10
Q96RT1	Oncology	97.7	195.3	12500	25000	1.8	7	13
O15357	Oncology	97.7	195.3	50000	100000	2.4	6	19
P23515	Oncology	6.1	12.2	12500	25000	3.0	12	12
P28907	Oncology	6.1	12.2	6250	25000	2.7	7	14
O60911	Oncology	24.4	48.8	6250	12500	2.1	4	6
Q7Z5A7	Oncology	48.8	97.7	6250	25000	1.8	9	14
P16870	Oncology	97.7	97.7	50000	800000	2.7	5	4
O60760	Oncology	781.3	1562.5	400000	800000	2.4	10	12
Q96EK5	Oncology	390.6	781.3	400000	800000	2.7	11	19
Q8N9I9	Oncology	97.7	195.3	400000	800000	3.3	10	9
O60825	Oncology	97.7	195.3	400000	800000	3.3	8	13
Q9UBM4	Oncology	12.2	24.4	25000	100000	3.0	10	12
O60763	Oncology	195.3	390.6	100000	400000	2.4	11	23

P07949	Oncology	24.4	48.8	25000	400000	2.7	8	10
Q8N386	Oncology	6.1	24.4	12500	50000	2.7	9	11
Q8NEZ2	Oncology						8	10
P15514	Oncology	1.5	3.1	6250	25000	3.3	8	10
P18627	Oncology						10	8
Q86SF2	Oncology	781.3	781.3	50000	800000	1.8	9	9
O00622	Oncology	48.8	97.7	25000	200000	2.4	9	9
O75144	Oncology	48.8	97.7	12500	800000	2.1	9	13
Q13576	Oncology						10	17
O00748	Oncology						10	13
P58499	Oncology	97.7	195.3	12500	800000	1.8	6	10
P26010	Oncology	12.2	48.8	50000	200000	3.0	4	6
Q9UKR3	Oncology	24.4	48.8	6250	50000	2.1	8	11
P49441	Oncology	1562.5	3125.0	400000	800000	2.1	11	29
O43570	Oncology	24.4	48.8	12500	100000	2.4	9	11
P37108	Oncology	390.6	781.3	50000	800000	1.8	9	11
P38936	Oncology	390.6	781.3	400000	800000	2.7	9	12
Q13561	Oncology						11	9
O14828	Oncology	195.3	195.3	50000	400000	2.4	10	9
P07948	Oncology	6.1	24.4	3125	6250	2.1	10	11
Q9NZT2	Oncology	48.8	97.7	200000	800000	3.3	10	10
P01275	Oncology						15	31
P50583	Oncology	97.7	195.3	12500	800000	1.8	8	13
Q9Y653	Oncology	195.3	390.6	100000	400000	2.4	7	14
Q8N129	Oncology	97.7	195.3	12500	800000	1.8	10	7
Q49AH0	Oncology	12.2	12.2	12500	50000	3.0	9	10
P29317	Oncology	12.2	24.4	25000	200000	3.0	7	9
Q9Y5K8	Oncology						2	8
O00451	Oncology						9	9
P29459_P29	Oncology	24.4	48.8	12500	100000	2.4	8	12
Q99795	Oncology	6.1	12.2	6250	12500	2.7	9	10
Q99536	Oncology						9	12
Q9GZV9	Oncology	97.7	195.3	50000	200000	2.4	5	6

Q9H156	Oncology	24.4	48.8	200000	800000	3.6	4	6
P98073	Oncology	48.8	195.3	25000	100000	2.1	9	9
Q9P0G3	Oncology						7	5
O43715	Oncology	48.8	97.7	25000	100000	2.4	4	3
Q9ULX7	Oncology	6.1	12.2	50000	400000	3.6	4	4
Q86SR1	Oncology	781.3	1562.5	400000	800000	2.4	11	15
Q9C005	Oncology	48.8	97.7	6250	25000	1.8	5	4
Q13421	Oncology						9	9
Q15116	Oncology	3.1	6.1	12500	25000	3.3	4	10
Q9UJM8	Oncology	97.7	195.3	400000	800000	3.3	4	7
P05187	Oncology	3.1	6.1	12500	50000	3.3	8	11
P25685	Oncology	3125.0	6250.0	800000	1600000	2.1	8	15
Q8WXI7	Oncology						14	22
P10145	Oncology	0.2	0.4	1563	12500	3.6	5	4
O43827	Oncology	48.8	97.7	25000	800000	2.4	3	5
P39900	Oncology	3.1	6.1	12500	100000	3.3	5	5
P09105	Oncology						4	9
P13521	Oncology	97.7	195.3	200000	400000	3.0	4	5
P50120	Oncology	48.8	195.3	12500	25000	1.8	8	8
P09960	Oncology	12500.0	25000.0	1600000	3200000	1.8	4	19
Q9HAV5	Oncology	0.4	0.8	3125	12500	3.6	4	5
P05089	Oncology	390.6	781.3	50000	200000	1.8	4	8
Q9H4F8	Oncology	781.3	1562.5	200000	800000	2.1	4	7
Q02742	Oncology	781.3	781.3	400000	800000	2.7	10	9
O14558	Oncology	48.8	97.7	25000	400000	2.4	3	6
Q14203	Oncology	195.3	781.3	400000	800000	2.7	4	9
Q9Y336	Oncology	12.2	24.4	6250	25000	2.4	5	3
P01303	Oncology	1562.5	3125.0	800000	800000	2.4	6	12
Q9H6B4	Oncology	24.4	48.8	12500	400000	2.4	3	8
P47929	Oncology	25000.0	25000.0	800000	1600000	1.5	6	13
Q6UWN8	Oncology	12.2	24.4	12500	100000	2.7	3	5
P40121	Oncology	781.3	781.3	400000	800000	2.7	3	8
Q16595	Oncology	3125.0	6250.0	800000	800000	2.1	4	5

Q8IXJ6	Oncology	24.4	48.8	50000	100000	3.0	10	12
P80511	Oncology	781.3	1562.5	400000	800000	2.4	9	17
Q86SJ2	Oncology	48.8	195.3	12500	50000	1.8	9	7
P98082	Oncology	195.3	390.6	50000	200000	2.1	4	12
Q9BXY4	Oncology	24.4	48.8	50000	200000	3.0	3	6
P06127	Oncology	0.8	1.5	3125	12500	3.3	5	6
P80303	Oncology	97.7	195.3	12500	400000	1.8	4	5
Q9NS68	Oncology	3.1	6.1	3125	12500	2.7	4	5
Q9H6S3	Oncology	97.7	195.3	100000	400000	2.7	4	4
O15263	Oncology						12	12
Q9Y5K2	Oncology	0.2	0.4	6250	25000	4.2	4	5
Q9P1Z2	Oncology	195.3	390.6	100000	400000	2.4	5	7
Q16653	Oncology	1.5	3.1	1563	3125	2.7	3	7
P08397	Oncology	390.6	781.3	200000	800000	2.4	5	8
Q7Z5L0	Oncology	1562.5	3125.0	200000	800000	1.8	13	11
Q96JA1	Oncology	48.8	97.7	100000	800000	3.0	4	5
Q16790	Oncology	6.1	6.1	6250	50000	3.0	4	6
P09758	Oncology	1.5	3.1	6250	25000	3.3	5	5
O60243	Oncology	390.6	781.3	200000	400000	2.4	8	8
Q9NPH0	Oncology	12.2	24.4	50000	400000	3.3	5	5
Q96I15	Oncology	97.7	195.3	100000	800000	2.7	3	5
P16562	Oncology	6.1	12.2	12500	200000	3.0	4	6
P27695	Oncology	6.1	12.2	6250	200000	2.7	3	5
Q02246	Oncology	24.4	48.8	12500	50000	2.4	4	5
Q9BZR6	Oncology	48.8	97.7	200000	800000	3.3	4	7
P62166	Oncology	12.2	24.4	25000	400000	3.0	4	5
Q10471	Oncology	195.3	390.6	50000	400000	2.1	3	6
Q8WWY7	Oncology	48.8	48.8	6250	12500	2.1	4	16
Q6PCB0	Oncology	48.8	195.3	400000	800000	3.3	4	4
P51858	Oncology	24.4	24.4	6250	25000	2.4	5	10
Q16775	Oncology	781.3	781.3	200000	800000	2.4	6	14
Q8TDQ1	Oncology	12.2	48.8	12500	25000	2.4	6	6
P02760	Oncology	1562.5	12500.0	12800000	12800000	3.0	5	6

Q9H3G5	Oncology	97.7	390.6	200000	800000	2.7	4	7
Q496F6	Oncology	390.6	781.3	400000	800000	2.7	3	4
P35052	Oncology	195.3	390.6	100000	400000	2.4	3	3
P56159	Oncology	24.4	48.8	50000	200000	3.0	3	4
P35475	Oncology	12.2	24.4	3125	12500	2.1	4	9
P32926	Oncology	3.1	6.1	6250	50000	3.0	6	5
Q96D42	Oncology	3.1	6.1	12500	50000	3.3	3	4
P20472	Oncology	12.2	12.2	6250	25000	2.7	9	9
O15123	Oncology	195.3	390.6	200000	800000	2.7	5	6
P29017	Oncology	97.7	195.3	25000	400000	2.1	8	12
Q14508	Oncology	12.2	24.4	25000	400000	3.0	7	6
O43895	Oncology	12.2	48.8	50000	200000	3.0	6	6
Q9UBX1	Oncology	97.7	195.3	50000	400000	2.4	7	8
P07237	Oncology	24.4	24.4	12500	50000	2.7	8	13
Q6FI81	Oncology	195.3	390.6	200000	400000	2.7	6	8
P41439	Oncology	0.2	0.4	12500	50000	4.5	6	10
Q5JTD0	Oncology	195.3	390.6	400000	800000	3.0	6	6
O14974	Oncology	97.7	195.3	25000	100000	2.1	9	22
P37173	Oncology	1.5	3.1	12500	25000	3.6	7	7
Q15303	Oncology	3.1	6.1	12500	50000	3.3	7	4
Q92832	Oncology	48.8	97.7	200000	800000	3.3	7	7
Q96NY8	Oncology	1.5	3.1	3125	12500	3.0	7	6
Q96J42	Oncology	48.8	97.7	25000	400000	2.4	8	10
Q9H8J5	Oncology	48.8	97.7	50000	400000	2.7	6	9
P21741	Oncology						8	14
Q9BYH1	Oncology	195.3	390.6	100000	400000	2.4	7	7
Q16543	Oncology	390.6	781.3	100000	400000	2.1	8	23
Q9NZ53	Oncology	48.8	97.7	25000	100000	2.4	8	13
P20851	Oncology	6.1	12.2	12500	100000	3.0	8	6
P41271	Oncology	24.4	48.8	12500	25000	2.4	7	8
P35916	Oncology	781.3	1562.5	400000	800000	2.4	7	5
P20138	Oncology	12.2	48.8	12500	50000	2.4	7	9
Q96SM3	Oncology	195.3	390.6	200000	400000	2.7	8	10

P35968	Oncology	3.1	6.1	25000	50000	3.6	6	10
Q02763	Oncology	48.8	97.7	100000	400000	3.0	7	9
P21589	Oncology	6.1	12.2	25000	100000	3.3	7	8
O95721	Oncology	48.8	97.7	100000	400000	3.0	12	13
P09486	Oncology	195.3	390.6	200000	800000	2.7	8	10
Q9UP79	Oncology	48.8	97.7	50000	200000	2.7	7	8
P32004	Oncology	97.7	195.3	100000	400000	2.7	6	8
O43464	Oncology	12.2	24.4	12500	25000	2.7	10	10
Q7Z4W1	Oncology	195.3	390.6	100000	800000	2.4	5	8
Q9HAT2	Oncology	195.3	390.6	50000	400000	2.1	6	5
Q8NCC3	Oncology	24.4	48.8	50000	400000	3.0	7	6
P21802	Oncology	12.2	24.4	25000	400000	3.0	6	6
Q14512	Oncology						7	8
Q9NP84	Oncology	97.7	195.3	200000	800000	3.0	9	11
O00244	Oncology	48.8	97.7	12500	800000	2.1	9	15
Q96PD2	Oncology	6.1	12.2	12500	50000	3.0	8	7
P78552	Oncology	24.4	48.8	12500	25000	2.4	6	9
P01298	Oncology	97.7	97.7	12500	50000	2.1	7	12
P13688	Oncology	6.1	12.2	25000	400000	3.3	8	8
P26447	Oncology						8	12
O75629	Oncology	97.7	195.3	25000	400000	2.1	6	12
P09958	Oncology	48.8	97.7	50000	100000	2.7	7	7
P48307	Oncology	0.8	1.5	6250	12500	3.6	7	8
P18084	Oncology	97.7	195.3	100000	400000	2.7	8	14
P15328	Oncology	0.8	1.5	3125	12500	3.3	8	9
O60259	Oncology	3.1	6.1	6250	25000	3.0	7	11
Q9UJ68	Oncology	0.8	1.5	391	1563	2.4	7	9
P26842	Oncology	24.4	48.8	25000	100000	2.7	7	8
P06870	Oncology	1.5	3.1	6250	25000	3.3	7	8
P12931	Oncology	24.4	48.8	12500	25000	2.4	7	14
O43240	Oncology	24.4	48.8	25000	50000	2.7	8	25
O95274	Oncology	6.1	12.2	6250	25000	2.7	6	15
O00548	Oncology	6.1	12.2	50000	100000	3.6	7	9

P49767	Oncology	97.7	97.7	12500	400000	2.1	11	13
P04626	Oncology	1.5	3.1	12500	50000	3.6	7	8
Q16674	Oncology	31.7	63.5	65000	130000	3.0	6	6
Q9UBX7	Oncology	48.8	97.7	25000	100000	2.4	7	40
Q92876	Oncology	1.5	12.2	3125	12500	2.4	7	5
Q6NT46	Oncology II	97.7	195.3	12500	400000	1.8	10	4
Q7Z460	Oncology II							
Q7Z4W2	Oncology II	6.1	12.2	1563	25000	2.1	9	23
Q9UPY8	Oncology II						2	3
O00337	Oncology II						5	11
P23771	Oncology II	100000.0	200000.0	6400000	12800000	1.5		
Q5VSG8	Oncology II						16	28
P43630	Oncology II	390.6	781.3	100000	400000	2.1	8	19
A7E2Y1	Oncology II	390.6	781.3	50000	400000	1.8	15	4
P30304	Oncology II	390.6	781.3	50000	100000	1.8	6	
Q9P2D8	Oncology II	781.3	781.3	100000	400000	2.1	14	20
Q14123	Oncology II						9	21
P12004	Oncology II	6250.0	6250.0	400000	3200000	1.8	3	
O60941	Oncology II	97.7	97.7	12500	50000	2.1	12	13
O43504	Oncology II						9	13
P19075	Oncology II	3.1	12.2	6250	25000	2.7	4	25
O60502	Oncology II	1562.5	3125.0	200000	800000	1.8	8	15
Q13443	Oncology II	25000.0	25000.0	1600000	3200000	1.8	9	19
Q93033	Oncology II	390.6	781.3	200000	800000	2.4	6	15
Q8NHZ8	Oncology II	1562.5	1562.5	50000	800000	1.5	6	40
Q92499	Oncology II						9	15
O75781	Oncology II	195.3	195.3	12500	50000	1.8	6	6
Q6UWK7	Oncology II						10	18
Q9UMS0	Oncology II	48.8	97.7	100000	400000	3.0		
Q96KB5	Oncology II	97.7	195.3	100000	400000	2.7	8	8
Q8ND71	Oncology II	195.3	390.6	25000	200000	1.8	16	21
O75330	Oncology II	3125.0	6250.0	800000	800000	2.1	9	13
Q14677	Oncology II	6250.0	12500.0	800000	800000	1.8	9	21

Q9GZT3	Oncology II						12	22
Q00994	Oncology II	195.3	390.6	25000	100000	1.8		
Q86SQ0	Oncology II						13	21
Q9NPI5	Oncology II							
Q9H9E1	Oncology II							
O43889	Oncology II	195.3	390.6	25000	800000	1.8	12	7
P48165	Oncology II	3125.0	3125.0	200000	800000	1.8		
Q9UQE7	Oncology II						9	
O15264	Oncology II	6250.0	6250.0	800000	800000	2.1	0.2	
P06493	Oncology II						15	8
Q15652	Oncology II	1562.5	3125.0	200000	800000	1.8	4	14
P00167	Oncology II	195.3	390.6	50000	400000	2.1	11	18
Q8N130	Oncology II	1562.5	3125.0	200000	800000	1.8	11	
Q6PUV4	Oncology II	781.3	1562.5	100000	400000	1.8		
Q9H741	Oncology II						13	19
P78395	Oncology II						5	
Q3MIW9	Oncology II	390.6	390.6	25000	100000	1.8	10	18
Q9UJZ1	Oncology II						11	8
Q12836	Oncology II	24.4	97.7	12500	50000	2.1	1	
A6NGN9	Oncology II	781.3	1562.5	200000	800000	2.1	8	14
A8MVZ5	Oncology II	3125.0	3125.0	200000	800000	1.8		
Q99259	Oncology II	6250.0	12500.0	800000	800000	1.8		
Q8N4E4	Oncology II	195.3	390.6	50000	200000	2.1	11	8
Q8WZ55	Oncology II	195.3	390.6	25000	200000	1.8		
P18848	Oncology II	48.8	48.8	6250	400000	2.1	12	
O00534	Oncology II	781.3	1562.5	100000	400000	1.8	10	17
P31689	Oncology II						0.3	
P31371	Oncology II						1	
Q8IYV9	Oncology II	97.7	195.3	200000	800000	3.0		
Q13017	Oncology II						9	14
Q12899	Oncology II							
Q15014	Oncology II	781.3	781.3	50000	400000	1.8	11	11
Q9H867	Oncology II						8	23

Q14674	Oncology II	390.6	781.3	50000	400000	1.8	11	18
P17980	Oncology II							
Q9NS37	Oncology II	6250.0	6250.0	800000	800000	2.1		
O43739	Oncology II	781.3	781.3	50000	400000	1.8		
Q9H293	Oncology II	195.3	390.6	100000	400000	2.4	4	
Q587J8	Oncology II						12	21
Q4VC05	Oncology II	781.3	781.3	50000	800000	1.8		
Q9NZQ9	Oncology II	1562.5	3125.0	200000	800000	1.8	7	
O75794	Oncology II	1562.5	1562.5	100000	800000	1.8	16	
Q8NDC4	Oncology II	390.6	390.6	100000	400000	2.4	10	20
Q8TE77	Oncology II	6250.0	12500.0	800000	3200000	1.8	9	3
Q13127	Oncology II						8	28
O94986	Oncology II	97.7	195.3	25000	50000	2.1	16	36
Q9GZP4	Oncology II	1562.5	1562.5	100000	400000	1.8	7	20
Q8TDX7	Oncology II	6250.0	6250.0	800000	800000	2.1	9	15
A8MTB9	Oncology II	195.3	390.6	50000	200000	2.1	10	12
Q9Y6I3	Oncology II						11	20
Q5VT06	Oncology II						18	
P01100	Oncology II	48.8	97.7	50000	200000	2.7		
P21781	Oncology II	195.3	195.3	12500	800000	1.8	14	13
P29353	Oncology II							
P08700	Oncology II	195.3	781.3	200000	800000	2.4		
P34910	Oncology II	781.3	1562.5	100000	400000	1.8	8	16
Q6P996	Oncology II	3125.0	3125.0	200000	400000	1.8		
Q9UN42	Oncology II						14	12
Q6PH85	Oncology II	1562.5	1562.5	100000	800000	1.8	2	
Q9UI15	Oncology II						15	
O75409	Oncology II	97.7	195.3	25000	50000	2.1		
Q969P6	Oncology II	3125.0	6250.0	400000	800000	1.8	9	10
Q9Y3C4	Oncology II	3125.0	6250.0	400000	3200000	1.8	11	
Q12849	Oncology II	390.6	781.3	50000	100000	1.8	16	21
A8MYV0	Oncology II	48.8	97.7	25000	100000	2.4		
P49756	Oncology II	3125.0	3125.0	200000	800000	1.8	4	

Q7Z6A9	Oncology II	195.3	195.3	12500	50000	1.8	18	33
O43422	Oncology II	48.8	97.7	25000	100000	2.4	14	26
Q86TS9	Oncology II						8	
P07992	Oncology II	781.3	3125.0	200000	800000	1.8		
Q96SD1	Oncology II						12	
Q13087	Oncology II	781.3	1562.5	200000	800000	2.1	13	18
Q9H0R8	Oncology II							
Q9ULR5	Oncology II						19	18
Q13972	Oncology II	97.7	195.3	25000	50000	2.1		
Q9NQP4	Oncology II						14	22
Q06609	Oncology II	24.4	97.7	50000	200000	2.7	7	
Q9UBU8	Oncology II							
Q14554	Oncology II						14	31
P27701	Oncology II	195.3	390.6	25000	50000	1.8	13	15
Q03111	Oncology II	781.3	781.3	100000	800000	2.1	21	
O95793	Oncology II						9	17
Q14055	Oncology II	3125.0	6250.0	400000	800000	1.8	12	25
Q9UNP9	Oncology II	1562.5	3125.0	400000	800000	2.1	14	6
Q00653	Oncology II	195.3	390.6	25000	200000	1.8		
Q9UHJ6	Oncology II	1562.5	3125.0	200000	800000	1.8	12	11
P42681	Oncology II	6250.0	12500.0	800000	800000	1.8	8	25
Q68DV7	Oncology II	390.6	781.3	100000	400000	2.1	12	18
O60603	Oncology II	195.3	390.6	100000	400000	2.4	16	30
P01112	Oncology II	97.7	195.3	25000	50000	2.1	6	9
Q03518	Oncology II						10	3
O15078	Oncology II	390.6	781.3	50000	100000	1.8	12	10
Q99487	Oncology II	3125.0	6250.0	400000	800000	1.8	14	17
Q9UHL9	Oncology II	781.3	1562.5	100000	400000	1.8	8	13
O43903	Oncology II	1562.5	1562.5	200000	800000	2.1	10	15
P47928	Oncology II	390.6	781.3	100000	200000	2.1	8	14
O14879	Oncology II	781.3	781.3	100000	200000	2.1	8	14
Q9H2G2	Oncology II						14	17
P49137	Oncology II	1562.5	1562.5	200000	800000	2.1	13	16

B0FP48	Oncology II	195.3	390.6	25000	50000	1.8	9	16
P43166	Oncology II	1562.5	3125.0	400000	800000	2.1		
Q13445	Oncology II	12500.0	25000.0	800000	6400000	1.5	20	
P11310	Oncology II						14	27
P49662	Oncology II	97.7	195.3	200000	400000	3.0	10	24
Q6N021	Oncology II	195.3	390.6	25000	50000	1.8	12	12
Q8TCU4	Oncology II	390.6	390.6	25000	50000	1.8	14	34
P59282	Oncology II						12	9
Q8WWU5	Oncology II	1562.5	1562.5	200000	800000	2.1	8	
Q9UK41	Oncology II	3125.0	6250.0	400000	800000	1.8	12	18
A6NLU5	Oncology II						5	21
O75665	Oncology II						7	11
O15164	Oncology II	195.3	390.6	50000	100000	2.1	14	21
O95777	Oncology II	6250.0	12500.0	800000	12800000	1.8	8	9
O43247	Oncology II							
P04183	Oncology II						10	3
Q16181	Oncology II						14	21
O15294	Oncology II	12500.0	25000.0	800000	800000	1.5	3	11
Q9HCM3	Oncology II						20	14
Q96F10	Oncology II	97.7	97.7	25000	50000	2.4	13	17
Q2M296	Oncology II						15	34
O60237	Oncology II	781.3	781.3	50000	200000	1.8	17	20
P51815	Oncology II	1562.5	3125.0	200000	800000	1.8	6	17
O95696	Oncology II	1562.5	1562.5	100000	800000	1.8	13	
P07766	Oncology II	97.7	195.3	100000	800000	2.7	15	
Q8TEW0	Oncology II						12	
P19526	Oncology II	3125.0	3125.0	200000	800000	1.8	11	14
P10398	Oncology II	1562.5	1562.5	100000	800000	1.8	12	7
P78358	Oncology II	48.8	97.7	25000	50000	2.4	17	
Q8WUY3	Oncology II	195.3	390.6	50000	200000	2.1	14	17
P22528	Oncology II						11	18
O15211	Oncology II	390.6	390.6	25000	50000	1.8	13	33
P15248	Oncology II	48.8	97.7	12500	50000	2.1	11	12

O75293	Oncology II	3125.0	3125.0	200000	3200000	1.8	9	16
Q6UY09	Oncology II	48.8	97.7	25000	100000	2.4	11	16
O00422	Oncology II	195.3	390.6	100000	400000	2.4	14	28
Q5VUJ9	Oncology II						13	13
Q9Y4G8	Oncology II						15	8
Q14204	Oncology II	195.3	390.6	100000	400000	2.4	6	11
P25092	Oncology II	390.6	390.6	50000	200000	2.1	14	
Q32MZ4	Oncology II						11	21
Q7Z6I6	Oncology II	3125.0	3125.0	200000	800000	1.8	16	
P50454	Oncology II	6250.0	6250.0	400000	800000	1.8		
Q96NB3	Oncology II	6250.0	6250.0	200000	800000	1.5	7	6
Q9UMX5	Oncology II	1562.5	3125.0	200000	800000	1.8	8	20
Q9ULD2	Oncology II	12.2	24.4	6250	50000	2.4	20	15
P43357	Oncology II	781.3	1562.5	100000	800000	1.8		
P78317	Oncology II							
Q9Y2J4	Oncology II	1562.5	3125.0	400000	800000	2.1	9	10
Q495A1	Oncology II	97.7	195.3	12500	200000	1.8	15	18
O14717	Oncology II	97.7	195.3	100000	200000	2.7	14	26
P30279	Oncology II	1562.5	3125.0	400000	800000	2.1	11	14
P26639	Oncology II	3125.0	6250.0	800000	800000	2.1	7	19
Q9UJ99	Oncology II	12500.0	12500.0	800000	3200000	1.8	10	36
Q8IV48	Oncology II						11	35
P33764	Oncology II	97.7	195.3	25000	50000	2.1	9	25
Q9NYJ8	Oncology II	3125.0	6250.0	400000	800000	1.8	14	22
P53420	Oncology II						19	
Q6UXC1	Oncology II	48.8	97.7	25000	50000	2.4	6	18
Q15796	Oncology II	25000.0	50000.0	3200000	12800000	1.8	8	13
Q92973	Oncology II						12	9
Q6ZVL6	Oncology II	1562.5	1562.5	100000	200000	1.8		
Q9HC77	Oncology II	195.3	195.3	25000	50000	2.1		
O43768	Oncology II	12500.0	12500.0	800000	1600000	1.8	12	25
Q8WWF5	Oncology II	781.3	781.3	100000	200000	2.1	7	23
P51948	Oncology II	390.6	781.3	200000	800000	2.4	7	8

P49366	Oncology II	781.3	781.3	25000	200000	1.5	13	20
Q92817	Oncology II	390.6	390.6	100000	200000	2.4	14	14
P78540	Oncology II	100000.0	100000.0	6400000	12800000	1.8	9	14
Q6P995	Oncology II	195.3	390.6	100000	400000	2.4	9	8
Q9NQ84	Oncology II	781.3	1562.5	400000	800000	2.4	11	20
P09914	Oncology II							
O95997	Oncology II	390.6	781.3	100000	800000	2.1	14	6
Q7Z6P3	Oncology II	1562.5	3125.0	200000	800000	1.8	12	19
Q9BW66	Oncology II	6250.0	12500.0	1600000	6400000	2.1	8	19
O43663	Oncology II	1562.5	3125.0	200000	800000	1.8	15	15
P18754	Oncology II	390.6	781.3	50000	200000	1.8	8	12
P20702	Oncology II	1562.5	1562.5	100000	800000	1.8	9	37
Q99963	Oncology II	6250.0	6250.0	400000	800000	1.8		
Q96AT9	Oncology II						12	18
P49454	Oncology II	390.6	390.6	50000	200000	2.1	13	26
Q6P5Z2	Oncology II	1562.5	1562.5	200000	800000	2.1	17	12
O75843	Oncology II	12.2	48.8	25000	200000	2.7	8	15
Q9UHY7	Oncology II						9	18
P21695	Oncology II	3125.0	3125.0	200000	800000	1.8	8	25
O15213	Oncology II	200000.0	200000.0	6400000	12800000	1.5	9	9
Q9P000	Oncology II	195.3	390.6	50000	200000	2.1	11	18
Q14641	Oncology II	97.7	195.3	25000	100000	2.1	14	17
O75460	Oncology II	195.3	390.6	100000	200000	2.4	9	13
Q8IYS2	Oncology II	12500.0	12500.0	800000	800000	1.8		
P52848	Oncology II	195.3	390.6	50000	200000	2.1	9	14
Q96IQ7	Oncology II	195.3	195.3	6250	50000	1.5	11	13
Q6B8I1	Oncology II	781.3	781.3	50000	400000	1.8	10	29
Q9GZZ8	Oncology II	6250.0	6250.0	400000	3200000	1.8	10	25
Q9HC57	Oncology II						11	12
Q16891	Oncology II	781.3	781.3	50000	800000	1.8	13	11
P56851	Oncology II	24.4	48.8	3125	12500	1.8	11	33
P63146	Oncology II						7	11
Q5VX71	Oncology II	48.8	48.8	25000	100000	2.7	14	13

Q9BZW2	Oncology II						9	12
Q92485	Oncology II	12500.0	12500.0	800000	3200000	1.8	9	16
P49354	Oncology II	24.4	48.8	12500	50000	2.4	9	16
P05091	Oncology II	781.3	1562.5	100000	400000	1.8	5	27
Q9UKM9	Oncology II	781.3	781.3	100000	800000	2.1	11	13
O95433	Oncology II						11	9
Q13287	Oncology II	195.3	390.6	200000	800000	2.7	12	22
Q06643	Oncology II	390.6	390.6	50000	200000	2.1	9	18
Q6PKG0	Oncology II	781.3	1562.5	200000	800000	2.1	13	21
Q9Y5E8	Oncology II	1562.5	3125.0	200000	800000	1.8	8	16
P35249	Oncology II	781.3	1562.5	200000	800000	2.1	7	9
Q8NEB7	Oncology II	195.3	390.6	25000	100000	1.8	12	12
Q9BU02	Oncology II	195.3	390.6	25000	200000	1.8	7	25
O60749	Oncology II						8	17
O75351	Oncology II	781.3	1562.5	100000	800000	1.8	7	32
Q6UW88	Oncology II	195.3	390.6	25000	100000	1.8	2	8
P20807	Oncology II	195.3	390.6	100000	800000	2.4	12	21
Q8N5J2	Oncology II							
Q96RE7	Oncology II	781.3	781.3	50000	200000	1.8	12	12
Q86TE4	Oncology II	195.3	390.6	25000	800000	1.8	11	10
Q8WUD1	Oncology II	3125.0	3125.0	200000	800000	1.8	12	21
Q9NUW8	Oncology II	195.3	390.6	25000	200000	1.8	19	36
A1KZ92	Oncology II	781.3	781.3	50000	400000	1.8	12	21
Q49A26	Oncology II						10	17
Q6UW15	Oncology II						18	13
P01266	Oncology II	6.1	12.2	3125	12500	2.4	8	16
Q9GZX6	Oncology II	12.2	24.4	6250	25000	2.4	13	15
Q8N0Z9	Oncology II	195.3	390.6	100000	400000	2.4	11	14
Q13410	Oncology II	3.1	6.1	3125	25000	2.7	13	14
P31785	Oncology II	390.6	781.3	50000	800000	1.8	12	13
Q8IY22	Oncology II	6250.0	12500.0	800000	3200000	1.8	8	16
Q9BW85	Oncology II	781.3	781.3	50000	200000	1.8	12	13
P36873	Oncology II	1562.5	3125.0	200000	800000	1.8	6	20

Q14160	Oncology II	195.3	390.6	50000	800000	2.1	6	20
P29536	Oncology II						14	24
Q765P7	Oncology II						14	32
Q7Z692	Oncology II	48.8	97.7	6250	50000	1.8	17	23
O95166	Oncology II	1562.5	1562.5	100000	400000	1.8	11	23
P19525	Oncology II						6	17
Q14BN4	Oncology II	97.7	195.3	12500	50000	1.8	7	21
A6NM11	Oncology II	781.3	1562.5	100000	400000	1.8	5	19
Q9Y3L3	Oncology II	1562.5	3125.0	200000	800000	1.8	6	29
O75528	Oncology II						13	21
Q9H8Y8	Oncology II	6250.0	6250.0	400000	1600000	1.8	7	25
Q96T91	Oncology II	97.7	97.7	6250	50000	1.8	5	7
P61812	Oncology II	12500.0	12500.0	400000	1600000	1.5	15	23
P21912	Oncology II	781.3	1562.5	200000	800000	2.1	7	35
Q96AQ6	Oncology II						11	9
Q8TBM8	Oncology II	48.8	97.7	25000	100000	2.4	8	13
Q9HD43	Oncology II	12.2	48.8	25000	200000	2.7	7	16
P84022	Oncology II	12500.0	12500.0	800000	800000	1.8	13	17
P43632	Oncology II	48.8	97.7	12500	200000	2.1	7	17
P43627	Oncology II	97.7	97.7	6250	50000	1.8	15	27
Q86UX2	Oncology II	781.3	1562.5	100000	400000	1.8	17	37
Q8NG06	Oncology II	390.6	390.6	25000	200000	1.8	7	11
Q9UHP3	Oncology II	97.7	195.3	12500	50000	1.8	7	14
P13051	Oncology II						12	28
Q9H2R5	Oncology II	97.7	97.7	12500	100000	2.1	13	20
Q9BRQ6	Oncology II	781.3	781.3	100000	400000	2.1	9	10
Q92783	Oncology II						10	14
P54652	Oncology II	195.3	781.3	50000	200000	1.8	11	13
Q6WCQ1	Oncology II						15	18
Q86SQ7	Oncology II	6.1	24.4	12500	50000	2.7	10	19
Q9H6E4	Oncology II	1562.5	3125.0	200000	800000	1.8	16	10
Q9ULC4	Oncology II						4	8
P51808	Oncology II						14	28

Q15599	Oncology II						9	20
Q05193	Oncology II	1562.5	3125.0	200000	800000	1.8	6	40
P33316	Oncology II	1562.5	3125.0	400000	800000	2.1	13	29
O60245	Oncology II	3125.0	3125.0	200000	800000	1.8	5	12
Q16549	Oncology II	1562.5	3125.0	200000	800000	1.8	18	22
P10415	Oncology II	195.3	195.3	50000	200000	2.4	11	19
O43805	Oncology II						6	18
Q96K21	Oncology II	1562.5	3125.0	400000	800000	2.1	5	29
Q6UW56	Oncology II	48.8	48.8	12500	50000	2.4	7	9
O60232	Oncology II	1562.5	3125.0	200000	800000	1.8	6	13
P11387	Oncology II						6	21
Q96A25	Oncology II	97.7	97.7	12500	50000	2.1	8	33
Q9BQE9	Oncology II	390.6	781.3	50000	800000	1.8	14	
Q9Y5Q6	Oncology II	97.7	97.7	12500	50000	2.1		
Q03169	Oncology II	48.8	48.8	12500	50000	2.4	5	7
P53384	Oncology II						5	
Q7Z4H3	Oncology II	195.3	390.6	50000	800000	2.1	6	8
P14902	Oncology II	195.3	390.6	100000	400000	2.4	7	14
Q16819	Oncology II	195.3	195.3	50000	800000	2.4	5	17
Q9P013	Oncology II	195.3	781.3	50000	200000	1.8	6	17
O00203	Oncology II	195.3	781.3	50000	800000	1.8	7	26
Q8IXQ3	Oncology II	195.3	390.6	25000	200000	1.8	5	19
O75940	Oncology II						8	14
Q68D85	Oncology II	195.3	390.6	100000	400000	2.4	6	12
Q9UH65	Oncology II	781.3	781.3	50000	800000	1.8	6	8
Q14011	Oncology II						7	37
P17181	Oncology II	195.3	195.3	25000	800000	2.1	5	7
Q676U5	Oncology II	781.3	1562.5	200000	800000	2.1	5	10
Q96RF0	Oncology II	12500.0	12500.0	800000	1600000	1.8	6	11
Q15172	Oncology II							
Q86UU1	Oncology II						21	22
O43312	Oncology II							
P20700	Oncology II						17	16

Q02750	Oncology II	195.3	390.6	200000	800000	2.7	4	28
Q99733	Oncology II	1562.5	1562.5	100000	800000	1.8	4	12
O43653	Oncology II						5	8
Q6NUJ1	Oncology II	12.2	97.7	6250	25000	1.8	5	12
P54577	Oncology II							
Q2WEN9	Oncology II	12.2	24.4	6250	50000	2.4	5	8
P42081	Oncology II	97.7	97.7	12500	200000	2.1	6	7
Q8WXX5	Oncology II						7	14
Q8IV16	Oncology II						23	25
Q63HQ2	Oncology II	781.3	1562.5	200000	800000	2.1	7	6
Q9UIM3	Oncology II	195.3	390.6	100000	400000	2.4	8	20
P21128	Oncology II	97.7	195.3	25000	50000	2.1	7	11
O75830	Oncology II	195.3	390.6	25000	200000	1.8	6	14
Q14246	Oncology II	1562.5	1562.5	100000	800000	1.8	8	10
Q96DE0	Oncology II						5	31
Q9Y5S2	Oncology II						6	14
O75071	Oncology II	25000.0	25000.0	1600000	3200000	1.8	7	6
Q8TF64	Oncology II	195.3	390.6	25000	200000	1.8	7	36
Q15262	Oncology II	3125.0	6250.0	800000	3200000	2.1	7	12
Q14258	Oncology II	781.3	781.3	50000	200000	1.8	6	16
P13284	Oncology II						5	11
Q674X7	Oncology II	781.3	1562.5	400000	800000	2.4	5	23
Q92890	Oncology II	781.3	781.3	200000	400000	2.4		
Q6PL24	Oncology II	97.7	195.3	25000	200000	2.1	5	32
Q7Z569	Oncology II	48.8	97.7	25000	800000	2.4	5	27
Q8N6M0	Oncology II						7	26
P53990	Oncology II							
O94988	Oncology II	97.7	390.6	25000	200000	1.8	6	25
Q17RW2	Oncology II						24	30
P49223	Oncology II	24.4	24.4	6250	25000	2.4		
Q99447	Oncology II	781.3	1562.5	100000	400000	1.8	6	22
Q96BQ1	Oncology II	48.8	97.7	12500	50000	2.1	4	18
Q9H910	Oncology II						8	39

P17568	Oncology II						9	33
Q9H2K0	Oncology II	781.3	3125.0	100000	800000	1.5		
Q9HC56	Oncology II	3125.0	6250.0	800000	800000	2.1	6	9
Q9NPJ3	Oncology II	781.3	781.3	25000	200000	1.5	8	37
Q6BCY4	Oncology II						6	9
P62330	Oncology II							
Q8IWZ8	Oncology II	3125.0	6250.0	200000	800000	1.5	11	17
P48060	Oncology II						14	19
Q96R05	Oncology II	781.3	1562.5	100000	200000	1.8	7	14
O15182	Oncology II	781.3	781.3	50000	400000	1.8	4	18

Table B. Diagnostic performance of multiprotein urine panels across disease groups

Disease	Optimal panel size	Mean AUC	95% CI (AUC)	Performance trend
**Bladder cancer**	9	0.98	0.93 – 0.99	Plateau from 5 proteins; 9-protein panel selected
**Cervical cancer**	7	0.95	0.86 – 0.98	Stable AUC from 7 proteins onward
**Endometrial cancer**	7	0.94	0.85 – 0.98	Plateau reached at 7 proteins
**Kidney cancer**	9	0.97	0.90 – 0.99	Plateau at 7 proteins; 9-protein panel selected
**Melanoma**	7	0.97	0.91 – 0.99	Narrow CI, early convergence
**Ovarian cancer**	15	0.97	0.93 – 0.99	Required larger panel for sensitivity
**Prostate cancer**	7	0.97	0.86 – 0.98	Balanced performance, low variance
**Multiple sclerosis**	5	0.88	0.73 – 0.96	Performance stable beyond 5 proteins
**MASH**	7	0.97	0.93 – 0.99	Consistent classification accuracy
